# Synthesis of dimeric analogs of adenophostin A that potently evoke Ca^2+^ release through IP_3_ receptors†
†Electronic supplementary information (ESI) available: NMR spectral data for all the new compounds. See DOI: 10.1039/c6ra19413c



**DOI:** 10.1039/c6ra19413c

**Published:** 2016-09-05

**Authors:** Amol M. Vibhute, Poornenth Pushpanandan, Maria Varghese, Vera Koniecnzy, Colin W. Taylor, Kana M. Sureshan

**Affiliations:** a School of Chemistry , Indian Institute of Science Education and Research Thiruvananthapuram , Kerala 695016 , India . Email: kms@iisertvm.ac.in ; http://kms514.wix.com/kmsgroup; b Department of Pharmacology , University of Cambridge , Tennis Court Road , Cambridge , CB2 1PD , UK

## Abstract

Inositol 1,4,5-trisphosphate receptors (IP_3_Rs) are tetrameric intracellular channels through which many extracellular stimuli initiate the Ca^2+^ signals that regulate diverse cellular responses. There is considerable interest in developing novel ligands of IP_3_R. Adenophostin A (AdA) is a potent agonist of IP_3_R and since some dimeric analogs of IP_3_R ligands are more potent than the corresponding monomer; we considered whether dimeric AdA analogs might provide agonists with increased potency. We previously synthesized traizolophostin, in which a simple triazole replaced the adenine of AdA, and showed it to be equipotent to AdA. Here, we used click chemistry to synthesize four homodimeric analogs of triazolophostin, connected by oligoethylene glycol chains of different lengths. We evaluated the potency of these analogs to release Ca^2+^ through type 1 IP_3_R and established that the newly synthesized dimers are equipotent to AdA and triazolophostin.

## Abstract 2

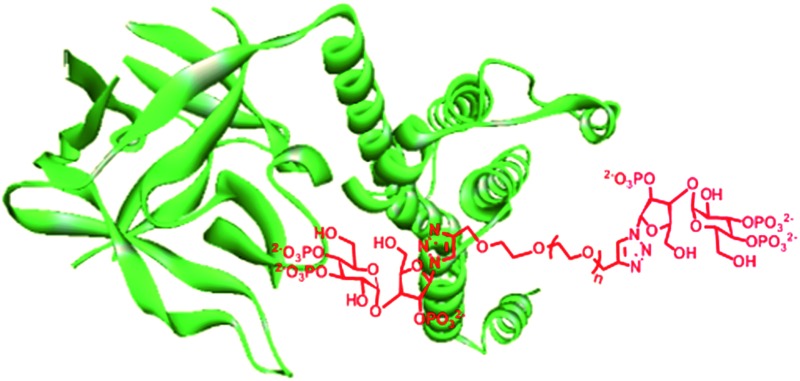
Syntheses and Ca^2+^ release potentials of four dimeric analogs of adenophostin A (AdA) through activation of type 1 IP_3_R are reported. These analogs are full agonists of IP_3_R and are equipotent to AdA, the most potent agonist of IP_3_R.

## Introduction

**Fig. 1:**
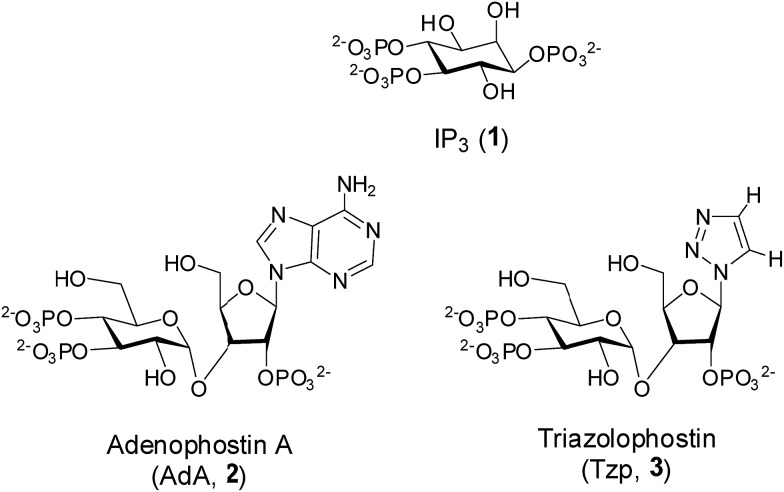
The structures of IP_3_ (**1**), adenophostin A (**2**) and triazolophostin (**3**).

Inositol 1,4,5-trisphosphate (IP_3_, **1**, Fig. 1) is an important secondary messenger that evokes Ca^2+^ release from intracellular stores through its interaction with IP_3_ receptors (IP_3_R) in the endoplasmic reticulum.^1^ IP_3_R are large tetrameric proteins, within which IP_3_ binding to each of the four subunits is required to initiate opening of the Ca^2+^-permeable channel.^2^ High-resolution structures of the IP_3_-binding core (IBC, residues 224–604) have defined the interactions of IP_3_ with IP_3_R.^3^ More recently, structures of the N-terminal region (residues 1–604)^4^ alongside a structure of the complete IP_3_R derived from cryo-electron microscopy have begun to suggest how IP_3_ binding might trigger the opening of the intrinsic pore of IP_3_R.^5^

There is continuing interest in the development of potent agonists and antagonists of IP_3_R.^6^ The fungal metabolite, adenophostin A (AdA, **2**, Fig. 1), binds to IP_3_R with greater affinity than IP_3_ and it is more potent than IP_3_ in evoking Ca^2+^ release.^7^ AdA analogs with a nucleobase or base-surrogate are also more potent than IP_3_.^8^ Molecular docking^8j,m,9^ and mutation studies^10^ suggest that a cation–π interaction between the adenine moiety of AdA and Arg504 within the IBC contributes to the increased affinity of AdA. We recently reported synthesis of a library of active AdA analogs, triazolophostins, by using a click chemistry approach.^11^ These potent analogs have substituted triazoles as adenine surrogates. The simplest analog, triazolophostin (**3**, Fig. 1) was equipotent with AdA.

Multimeric ligands often have greater affinity than monomeric ligands.^12^ This can be due to simultaneous binding to more than one binding site or a statistical effect arising from the local increase in ligand concentration.^13^ The former is unlikely for IP_3_R because the orientation of the IP_3_-binding sites within the tetrameric IP_3_R is unlikely to allow simultaneous binding of two ligands linked by a short tether.^4b,14^

**Fig. 2:**
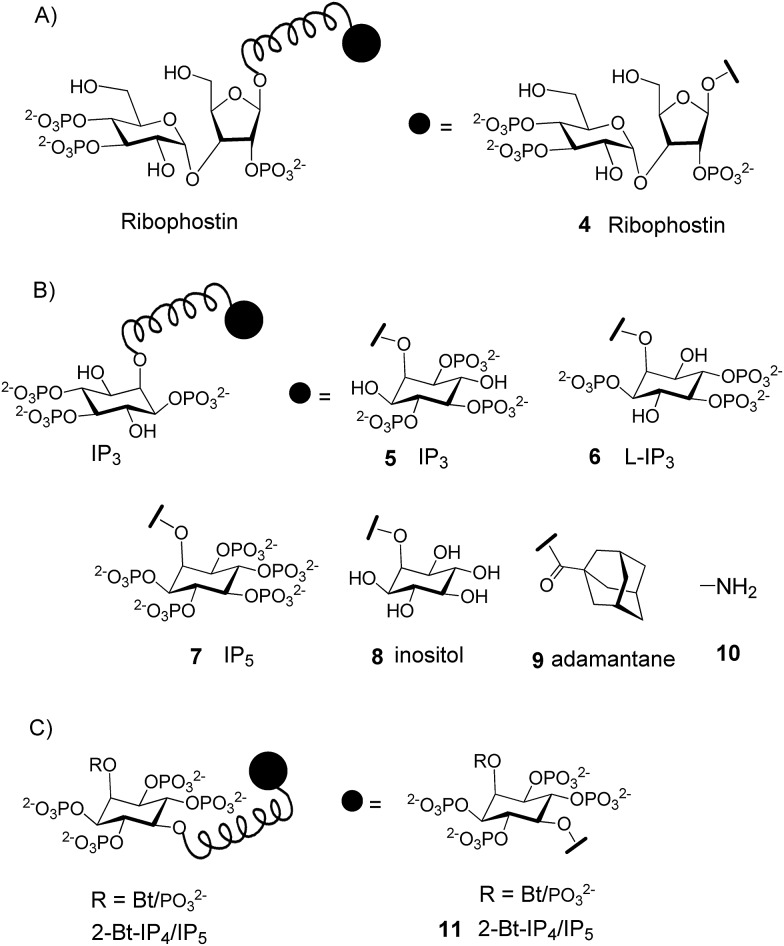
The representative structures of (A) ribophostin dimer **4**, (B) homo and hetero dimers of IP_3_ (**5–10**) and (C) dimers of 2-Bt-IP_4_/IP_5_ **11**.

A few multimeric ligands of IP_3_R have been reported. Before the location of the IP_3_-binding sites within IP_3_R was known, clustered bi- and tetra-dentate analogs of ribophostin (**4**, Fig. 2A) were synthesized, anticipating that if the spacing between the linked ligands was appropriate they might bind simultaneously to the four IP_3_-binding sites.^15^ However, the potencies of the monomeric and polymeric ligands were rather similar. Several homodimeric^16^ and heterodimeric^17^ ligands of IP_3_ (**5–10**, Fig. 2B), particularly those with short linkers, were shown to bind to IP_3_R with increased affinity.^13*d*^ Very recently, dimers of 2-*O*-Bt-IP_4_/IP_5_ (**11**, Fig. 2C) were shown to be antagonists of IP_3_Rs.^18^ These results demonstrate that dimeric IP_3_R ligands can provide useful tools, some of which have greater affinity than the monomeric ligands. We therefore considered whether dimers of AdA might be more potent than AdA.

## Results and discussion

**Fig. 3:**
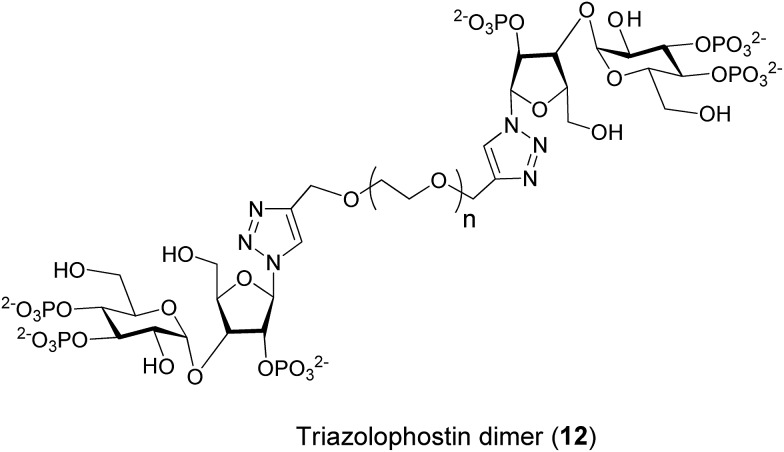
The structure of dimeric analogs of triazolophostin **12**.

**Scheme 1:**
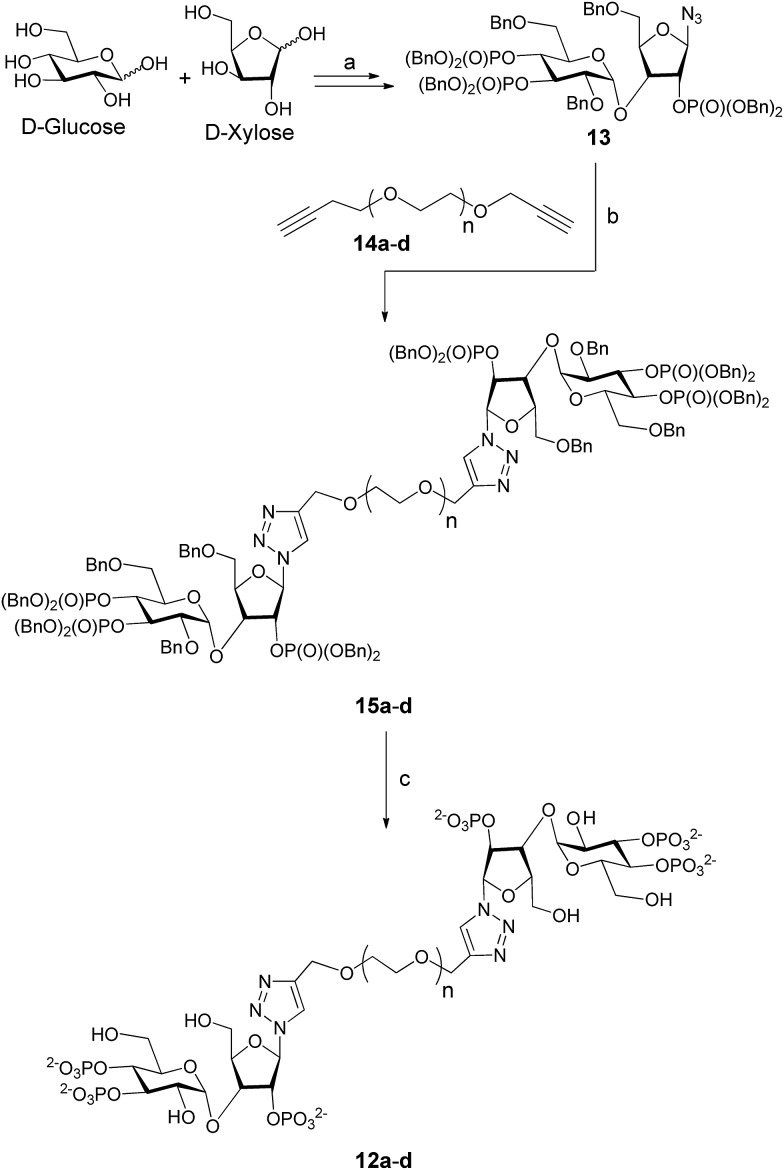
Synthesis of triazolophostin dimers. Reagents and conditions: (a) ref. 11; (b) Cu, CuSO_4_, H_2_O : ^*t*^BuOH (1 : 1, v/v), rt, 24 h; (c) Pd(OH)_2_/C, cyclohexene, MeOH : H_2_O (10 : 1, v/v), 80 °C. 4 h; (a), *n* = 2; (b), *n* = 3; (c), *n* = 4; (d), *n* = 6.

As the synthesis of AdA dimers is challenging, we decided to make oligoethylene glycol-tethered dimers of triazolophostin (Fig. 3). We envisaged that use of click reaction^19^ with a linker connected to alkyne at both termini would ensure both formation of triazole and link the two monomers in one step. Previous studies suggested that short linkers were most likely to improve the affinity of homodimers.^13*d*^ We therefore selected spacers smaller than hexaethylene glycol. The linkers **14a–d** were synthesized by slightly modifying previously reported procedures.^20^ The oligoethylene glycols were first co-evaporated with toluene and then treated with sodium hydride in the presence of excess propargyl bromide to get dipropargyl polyethylene glycols **14a–d** in good to excellent yields. The azide **13** was synthesized from glucose and xylose by several protection–deprotection reactions followed by phosphorylation as reported earlier.^11^ The azide **13** was then treated with dialkynyl polyethylene glycols **14a–d** in the presence of Cu(i) catalyst to get fully protected triazolophostin dimers **15a–d** in good yields. The debenzylation of protected triazolophostin dimers **15a–d** was carried out using transfer hydrogenolysis in the presence of palladium and cyclohexene under reflux condition and the products were purified by ion-exchange chromatography to yield dimers **12a–d**, in excellent yields (Scheme 1).

**Fig. 4:**
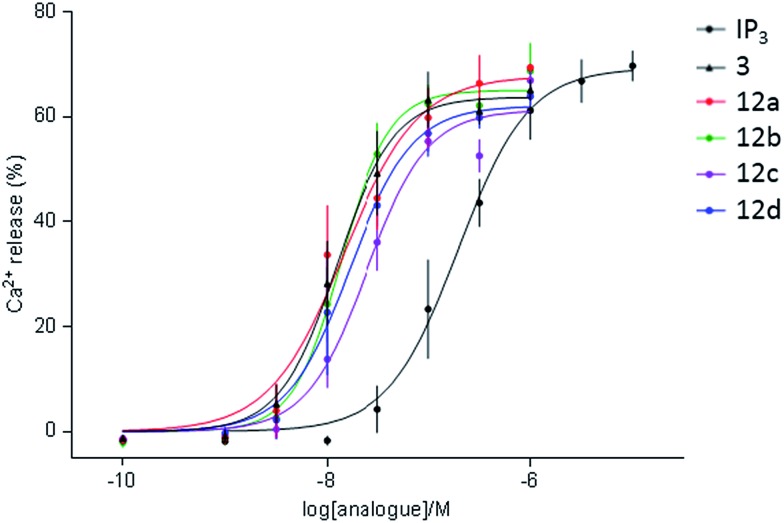
Summary of Ca^2+^ release from permeabilized DT40-IP_3_R1 cells evoked by IP_3_, monomer **3** and its dimeric analogs **12a–d**.

The dimeric ligands **12a–d** were screened for their abilities to evoke Ca^2+^ release through IP_3_R (Table 1, Fig. 4). All four dimers were full agonists of IP_3_R, more potent than IP_3_, but similar in their potency to AdA and the monomer, triazolophostin. The similar potencies of **12a–d** irrespective of their tether length suggest that these ligands might be interacting with IP_3_R1 in monodentate fashion.

**Table 1:** Responses of IP_3_R1 to IP_3_ (**1**), monomer (**3**) and its dimeric analogs **12a–d** ^*a*^

Ligand	pEC_50_	EC_50_ (nM)	EC_50_ w.r.t. **1** ^*b*^	Max. response (%)	*n*_H_
IP_3_ (**1**)	6.72 ± 0.12	190.5	1	69 ± 3	1.40 ± 0.16
Monomer (**3**)	7.86 ± 0.17	13.8	13.8	65 ± 1	1.66 ± 0.21
**12a**	7.83 ± 0.18	14.8	12.9	68 ± 2	1.33 ± 0.12
**12b**	7.85 ± 0.13	14.1	13.5	66 ± 1	1.89 ± 0.13
**12c**	7.62 ± 0.11	24.0	7.9	61 ± 3	1.60 ± 0.16
**12d**	7.84 ± 0.12	14.4	13.2	60 ± 1	1.94 ± 0.47

^*a*^Maximal Ca^2+^ release, the half-maximally effective ligand concentration (EC_50_), –log EC_50_ (pEC_50_) and Hill coefficient (*n*_H_) are shown as means ± SEM (*n* = 3).

^*b*^The EC_50_ value of each ligand is also shown relative to that for IP_3_ (**1**) (EC**1**50/ECanalog50).

## Conclusions

In conclusion, based on several previous reports that dimeric IP_3_R ligands can be more potent than the corresponding monomers, we anticipated that dimers of AdA might have increased potency. We used click chemistry to synthesize dimers of a potent analog of AdA (triazolophostin) linked by spacers of different length. In assays of Ca^2+^ release through IP_3_R, the dimeric ligands were no more potent than the corresponding monomer (**3**). This suggests that whereas dimeric derivatives of IP_3_ have reduced efficacy but improved affinity,^10,21^ dimerization of AdA analogs does not improve their affinity.

## Experimental section

### General methods

The chemicals were purchased from commercial sources and used as received. The TLC plates were visualized under UV light and by dipping plates into either phosphomolybdic acid in MeOH or sulphuric acid in ethanol, followed by heating. All NMR experiments were carried out on a 500 MHz NMR spectrometer and at room temperature. Tetramethylsilane (TMS, *δ* 0.0 ppm) or the solvent reference (CDCl_3_, *δ* 7.26 ppm; D_2_O, *δ* 4.79 ppm) relative to TMS were used as the internal standard. The data are reported as follows: chemical shift in ppm (*δ*) (multiplicity [singlet (s), doublet (d), doublet of doublet (dd), triplet (t), quartet (q), and multiplet (m)], coupling constants [Hz], integration and peak identification). All NMR signals were assigned on the basis of ^1^H NMR, ^13^C NMR, COSY and HMQC experiments. ^13^C NMR spectra were recorded with complete proton decoupling. Carbon chemical shifts are reported in ppm (*δ*) relative to TMS with the respective solvent resonance as the internal standard. The concentration of the compounds for ^1^H NMR was 5 mg per 0.5 mL and for ^13^C NMR it was 20 mg per 0.5 mL for protected compounds and 5–7 mg per 0.5 mL for final compounds in case of ^1^H and ^13^C NMR. Modified Brigg's phosphate assay^22^ was employed to quantify each triazolophostin **12a–d**. Silica gel 230–400 mesh was used to perform flash column chromatography.

### General procedure for syntheses of fully protected triazolophostin dimers

To a solution of azide **13** (0.144 mmol) and dialkynyl PEG **14a–d** (0.072) in H_2_O/^*t*^BuOH (1/1, v/v, 2 mL) was added Cu (0.036 g, 0.57 mmol) and CuSO_4_ (8 mg, 0.028 mmol) and stirred at room temperature for 24 h. The reaction was monitored by TLC. When the TLC showed complete disappearance of the azide **13**, the mixture was filtered through a Celite bed and was partitioned between ethyl acetate and water. The organic layer was washed with brine. The organic layer was dried over anhyd. sodium sulphate, filtered and concentrated under reduced pressure. The residue thus obtained was purified by flash column chromatography using a mixture of acetone, diethyl ether and petroleum ether (4 : 2 : 15 v/v/v) as eluent to get pure **15a–d** as a colourless gum.

#### Protected triazolophostin dimer **15a**

Click reaction of azide **13** (0.2 g, 0.144 mmol) with diyne **14a** (0.011 g, 0.072 mmol) gave the protected dimer **15a** (0.18 g, 85%) as a colourless gum. ^1^H NMR (500 MHz, CDCl_3_) *δ*: 3.47–3.57 (m, 18H, H-2′′, H-4′′, H-6′′_A_, and DEG-H), 3.73–3.75 (m, 2H, H-5′′), 4.20–4.23 (m, 2H, PhC*H*_2_), 4.27–4.32 (m, 8H, H-5′_A_ and PhC*H*_2_), 4.30–4.45 (m, 10H, H-3′, H-4′, H-5′_B_, H-6′′_B_ and PhC*H*_2_), 4.57–4.59 (m, 2H, PhC*H*_2_), 4.63–4.66 (m, 4H, PhC*H*_2_), 4.68–4.73 (m, 6H, PhC*H*_2_), 4.80–4.93 (m, 16H, H-3′′, H-4′′ and PhC*H*_2_), 5.11 (d, 2H, *J* = 3.2 Hz, H-1′′), 5.26–5.28 (m, 2H, H-2′) 6.24 (d, 2H, *J* = 5.0 Hz, H-1′), 7.00 (d, 4H, *J* = 7.0 Hz, Ar-*H*), 7.05–7.19 (m, 82H, Ar-*H*), 7.26 (d, 4H, *J* = 7.0 Hz, Ar-*H*), 7.60 (s, 2H, H-5); ^13^C NMR (125 MHz, CDCl_3_) *δ*: 64.2, 68.3, 69.1, 69.2, 69.3, 69.5, 69.6, 69.7, 69.9, 70.1, 70.4, 71.9, 73.3, 73.5, 74.1, 78.0, 78.5, 82.8, 90.1, 95.7, 121.6, 127.6, 127.7, 127.9, 128.0, 128.1, 128.3, 128.4, 128.5, 135.2, 135.7, 135.8, 136.1, 137.3, 137.5, 138.0, 145.2; ^31^P NMR (202.4 MHz, CDCl_3_) *δ*: –1.484, –1.928, –2.146; HRMS (ESI) mass calcd for C_158_H_166_N_6_O_39_P_6_ [M]^+^ 2956.9616, found 2956.9620.

#### Protected triazolophostin dimer **15b**

Click reaction of azide **13** (0.2 g, 0.144 mmol) with diyne **14b** (0.016 g, 0.072 mmol) gave the protected dimer **15b** (0.185 g, 86%) as a colourless gum. ^1^H NMR (500 MHz, CDCl_3_) *δ*: 3.44–3.57 (m, 22H, H-2′′, H-4′′, H-6′′_A_, and TEG-H), 3.75 (bs, 2H, H-5′′), 4.21–4.30 (m, 10H, H-5′_A_ and PhC*H*_2_), 4.42–4.43 (m, 10H, H-3′, H-4′, H-5′_B_, H-6′′_B_ and PhC*H*_2_), 4.57–4.59 (m, 2H, PhC*H*_2_), 4.64–4.66 (m, 6H, PhC*H*_2_), 4.68–4.73 (m, 4H, PhC*H*_2_), 4.84–4.92 (m, 16H, H-3′′, H-4′′ and PhC*H*_2_), 5.11 (bs, 2H, H-1′′), 5.27 (bs, 2H, H-2′) 6.24 (d, 2H, *J* = 5.0 Hz, H-1′), 7.00–7.25 (m, 90H, Ar-*H*), 7.61 (s, 2H, H-5); ^13^C NMR (125 MHz, CDCl_3_) *δ*: 63.2, 67.3, 68.0, 68.3, 68.4, 68.6, 68.7, 69.1, 69.4, 70.9, 72.3, 75.7, 75.8, 75.9, 76.9, 77.5, 81.7, 89.0, 94.7, 120.6, 126.7, 127.0, 127.2, 127.4, 134.2, 134.6, 135.1, 136.3, 136.5, 137.0, 144.2; ^31^P NMR (202.4 MHz, CDCl_3_) *δ*: –1.486, –1.935, –2.155; HRMS (ESI) mass calcd for C_160_H_170_N_6_O_40_P_6_ [M]^+^ 3000.9879, found 3000.9877.

#### Protected triazolophostin dimer **15c**

The reaction of azide **13** (0.2 g, 0.144 mmol) with diyne **14c** (0.019 g, 0.072 mmol) gave the protected dimer **15c** (0.175 g, 81%) as a colourless gum. ^1^H NMR (500 MHz, CDCl_3_) *δ*: 3.54–3.67 (m, 26H, H-2′′, H-4′′, H-6′′_A_, and TetraEG-H), 3.84 (bs, 2H, H-5′′), 4.30–4.32 (m, 2H, PhC*H*_2_), 4.37–4.39 (m, 8H, H-5′_A_ and PhC*H*_2_), 4.48–4.53 (m, 10H, H-3′, H-4′, H-5′_B_, H-6′′_B_ and PhC*H*_2_), 4.66–4.68 (m, 2H, PhC*H*_2_), 4.73–4.74 (m, 4H, PhC*H*_2_), 4.78–4.82 (m, 6H, PhC*H*_2_), 4.92–4.94 (m, 10H, H-3′′, H-4′′ and PhC*H*_2_), 4.97–5.03 (m, 6H, PhC*H*_2_), 5.20 (bs, 2H, H-1′′), 5.36 (bs, 2H, H-2′) 6.34 (d, 2H, *J* = 5.0 Hz, H-1′), 7.09–7.34 (m, 90H, Ar-*H*), 7.75 (s, 2H, H-5); ^13^C NMR (125 MHz, CDCl_3_) *δ*: 64.2, 68.3, 69.1, 69.15, 69.2, 69.3, 69.39, 69.5, 69.5, 69.6, 69.8, 69.9, 70.4, 70.5, 70.55, 71.9, 73.3, 73.5, 76.7, 82.8, 95.7, 121.6, 127.6, 127.7, 127.78, 127.8, 127.9, 128.0, 128.1, 128.3, 128.37, 128.4, 128.5, 128.55, 128.6, 135.2, 136.1, 136.2, 137.3, 137.5, 138.0; ^31^P NMR (202.4 MHz, CDCl_3_) *δ*: –1.468, –1.908, –2.138; HRMS (ESI) mass calcd for C_162_H_174_N_6_O_41_P_6_ [M]^+^ 3045.0141, found 3045.0131.

#### Protected triazolophostin dimer **15d**

The reaction of azide **13** (0.2 g, 0.144 mmol) with diyne **14d** (0.026 g, 0.072 mmol) gave the protected dimer **15d** (0.185 g, 82%) as a colourless gum. ^1^H NMR (500 MHz, CDCl_3_) *δ*: 3.53 (bs, 34H, H-2′′, H-4′′, H-6′′_A_, and HEG-H), 3.74 (bs, 2H, H-5′′), 4.23–4.28 (m, 10H, H-5′_A_ and PhC*H*_2_), 4.42 (bs, 10H, H-3′, H-4′, H-5′_B_, H-6′′_B_ and PhC*H*_2_), 4.56–4.58 (m, 2H, PhC*H*_2_), 4.65–4.71 (m, 10H, PhC*H*_2_), 4.83–4.91 (m, 16H, H-3′′, H-4′′ and PhC*H*_2_), 5.11 (bs, 2H, H-1′′), 5.27 (bs, 2H, H-2′) 6.24 (d, 2H, *J* = 5.0 Hz, H-1′), 6.99–7.24 (m, 90H, Ar-*H*), 7.62 (s, 2H, H-5); ^13^C NMR (125 MHz, CDCl_3_) *δ*: 64.2, 69.1, 69.16, 69.2, 69.3, 69.4, 69.5, 69.6, 69.7, 69.8, 69.9, 70.0, 70.4, 70.5, 71.9, 73.3, 73.5, 82.8, 95.7, 127.5, 127.8, 127.7, 127.75, 127.76, 127.8, 127.9, 128.0, 128.1, 128.2, 128.3, 128.4, 128.46, 128.49, 128.5, 128.6, 135.2, 136.1, 137.3, 137.5, 138.0; ^31^P NMR (202.4 MHz, CDCl_3_) *δ*: –1.482, –1.919, –2.168; HRMS (ESI) mass calcd for C_166_H_182_N_6_O_43_P_6_ [M]^+^ 3133.0665, found 3133.0669.

### General procedure for syntheses of triazolophostin dimers **12a–d**

The protected triazolophostin dimers **15a–d** (0.15–0.175 g, 0.05–0.055 mmol) were treated with cyclohexene (3 mL) and Pd(OH)_2_ (20% on carbon, 50 mg) in a mixture of methanol and water (9 : 1 v/v, 10 mL) at 80 °C for 4 h. The reaction mixture was then cooled, filtered through a membrane filter, washed successively with methanol and water. The combined filtrate was evaporated under reduced pressure. The crude product thus obtained was purified by ion-exchange column chromatography on Q-Sepharose matrix using 0–1.0 M TEAB as eluent to get pure triazolophostin dimers **12a–d**.

#### Triazolophostin dimer **12a**

The global debenzylation of **15a** (0.15 g, 0.05 mmol) gave 46 mg (69%) of triazolophostin dimer **12a** as a white hygroscopic solid: ^1^H NMR (500 MHz, D_2_O) *δ*: 3.63–3.65 (m, 8H, DEG-H), 3.70–3.83 (m, 12H, H-5′_A_, H-2′′, H-6′′ and DEG-H), 4.09–4.10 (m, 2H, H-5′′), 4.41 (bs, 2H, H-4′), 4.48 (bs, 2H, H-5′_B_), 4.62–4.65 (m, 6H, H-3′, H-3′′ and H-4′′), 5.16 (bs, 2H, H-2′), 5.24 (bs, 2H, H-1′′), 6.36 (bs, 2H, H-1′), 8.22 (s, 2H, H-5); ^13^C NMR (125 MHz, D_2_O) *δ*: 60.1, 60.7, 62.8, 68.8, 69.4, 70.5, 71.5, 72.8, 73.7, 76.4, 77.9, 83.8, 90.9, 97.9, 124.3, 144.1; ^31^P NMR (202.4 MHz, D_2_O) *δ*: 3.504, 3.583, 4.301; HRMS (ESI) mass calcd for C_32_H_58_N_6_O_39_P_6_ [M]^+^, 1336.1165, found: 1336.1169.

#### Triazolophostin dimer **12b**

The global debenzylation of **15b** (0.155 g, 0.051 mmol) gave 51 mg (72%) of triazolophostin dimer **12b** as a white hygroscopic solid: ^1^H NMR (500 MHz, D_2_O) *δ*: 3.56–3.60 (m, 12H, TEG-H), 3.69–3.74 (m, 12H, H-5′_A_, H-2′′, H-6′′ and TEG-H), 4.06 (bs, 2H, H-5′′), 4.36 (bs, 2H, H-4′), 4.44 (bs, 2H, H-5′_B_), 4.50–4.60 (m, 6H, H-3′, H-3′′ and H-4′′), 5.12 (bs, 2H, H-2′), 5.18 (bs, 2H, H-1′′), 6.31 (bs, 2H, H-1′), 8.18 (s, 2H, H-5); ^13^C NMR (125 MHz, D_2_O) *δ*: 60.1, 60.7, 62.8, 68.8, 69.4, 69.48, 70.4, 71.5, 72.8, 73.7, 76.4, 77.8, 83.8, 90.8, 97.9, 124.3, 144.1; ^31^P NMR (202.4 MHz, D_2_O) *δ*: 3.451 (2 × P), 4.224; HRMS (ESI) mass calcd for C_34_H_62_N_6_O_40_P_6_ [M]^+^, 1380.1427, found: 1380.1420.

#### Triazolophostin dimer **12c**

The global debenzylation of **15c** (0.16 g, 0.052 mmol) gave 64 mg (85%) of triazolophostin dimer **12c** as a white hygroscopic solid: ^1^H NMR (500 MHz, D_2_O) *δ*: 3.57–3.61 (m, 16H, TetraEG-H), 3.69–3.74 (m, 12H, H-5′_A_, H-2′′, H-6′′ and TetraEG-H), 4.05 (bs, 2H, H-5′′), 4.37 (bs, 2H, H-4′), 4.44 (bs, 2H, H-5′_B_), 4.58–4.61 (m, 6H, H-3′, H-3′′ and H-4′′), 5.12 (bs, 2H, H-2′), 5.19 (bs, 2H, H-1′′), 6.32 (bs, 2H, H-1′), 8.19 (s, 2H, H-5); ^13^C NMR (125 MHz, D_2_O) *δ*: 60.1, 60.7, 62.9, 68.9, 69.4, 69.5, 70.5, 71.5, 72.8, 73.7, 76.4, 77.9, 83.8, 90.8, 98.0, 124.3, 144.0; ^31^P NMR (202.4 MHz, D_2_O) *δ*: 3.478 (2 × P), 4.259; HRMS (ESI) mass calcd for C_36_H_66_N_6_O_41_P_6_ [M]^+^, 1424.1690, found: 1424.1699.

#### Triazolophostin dimer **12d**

The global debenzylation of **15d** (0.175 g, 0.055 mmol) gave 65 mg (77%) of triazolophostin dimer **12d** as a white hygroscopic solid: ^1^H NMR (500 MHz, D_2_O) *δ*: 3.58–3.72 (m, 24H, HEG-H), 3.77–3.81 (m, 12H, H-5′_A_, H-2′′, H-6′′ and HEG-H), 4.01 (bs, 2H, H-5′′), 4.38–4.48 (m, 4H, H-4′ and H-5′_B_), 4.58–4.63 (m, 6H, H-3′, H-3′′ and H-4′′), 5.12 (bs, 2H, H-2′), 5.20 (bs, 2H, H-1′′), 6.32 (bs, 2H, H-1′), 8.19 (s, 2H, H-5); ^13^C NMR (125 MHz, D_2_O) *δ*: 60.2, 60.8, 62.9, 68.9, 69.4, 69.5, 70.8, 71.7, 72.6, 73.7, 76.3, 77.4, 83.8, 90.9, 97.9, 124.2, 144.2; ^31^P NMR (202.4 MHz, D_2_O) *δ*: 3.482 (2 × P), 4.258; HRMS (ESI) mass calcd for C_40_H_74_N_6_O_43_P_6_ [M]^+^, 1512.2214, found: 1512.2210.

### Biological assay

Ca^2+^ release from the intracellular stores of saponin-permeabilized DT40 cells expressing only type 1 IP_3_Rs was measured using a low-affinity Ca^2+^ indicator (Mag-fluo-4) trapped within the endoplasmic reticulum as described previously.^11^ Briefly, Ca^2+^ uptake was initiated by addition of 1.5 mM MgATP in cytosol-like medium (140 mM KCl, 20 mM NaCl, 1 mM EGTA, 20 mM PIPES, pH 7.0, free [Ca^2+^] ∼220 nM after addition of ATP) containing *p*-trifluoromethoxyphenylhydrazone (FCCP) to inhibit mitochondria. After about 120 s, the triazolophostin analogs were added with cyclopiazonic acid (10 μM) to inhibit further Ca^2+^ uptake. Ca^2+^ release was assessed 10–20 s after addition of the analog, and expressed as a fraction of the ATP-dependent Ca^2+^ uptake.
