# Supplementary material for: Synthesis of dimeric analogs of adenophostin A that potently evoke Ca2+ release through IP3 receptors
Source: RSC Adv. 2016 Sep 5;6(89):86346–51. doi: 10.1039/c6ra19413c (PMC5171214; doi:10.1039/c6ra19413c)

Electronic Supplementary Material (ESI) for RSC Advances.
This journal is © The Royal Society of Chemistry 2016

Electronic Supplementary Information

Synthesis of Dimeric Analogs of Adenophostin A that Potently Evoke Ca^{2+} Release through IP_3 Receptors

Amol M. Vibhute,[a] Poornenth Pushpanandan,[a] Maria Varghese,[a] Vera Koniecznzy,[b] Colin W. Taylor[b] and Kana M. Sureshan[a]*

[a]*School of Chemistry, Indian Institute of Science Education and Research Thiruvananthapuram, KERALA-695016, India. E-mail:* kms@iisertvm.ac.in *Home page:* http://kms514.wix.com/kmsgroup

[b]*Department of Pharmacology, Tennis Court Road, University of Cambridge, Cambridge, CB2, 1PD, UK.*

Index

1. NMR data of protected dimer **15a** S2- S7
2. NMR data of protected dimer **15b** S8- S13
3. NMR data of protected dimer **15c** S14- S19
4. NMR data of protected dimer **15d** S20- S25
5. NMR data of dimer **12a** S26- S30
6. NMR data of dimer **12b** S31- S35
7. NMR data of dimer **12c** S36- S40
8. NMR data of dimer **12d** S41- S46

^{1}H NMR of 15a in $CDCl_3$

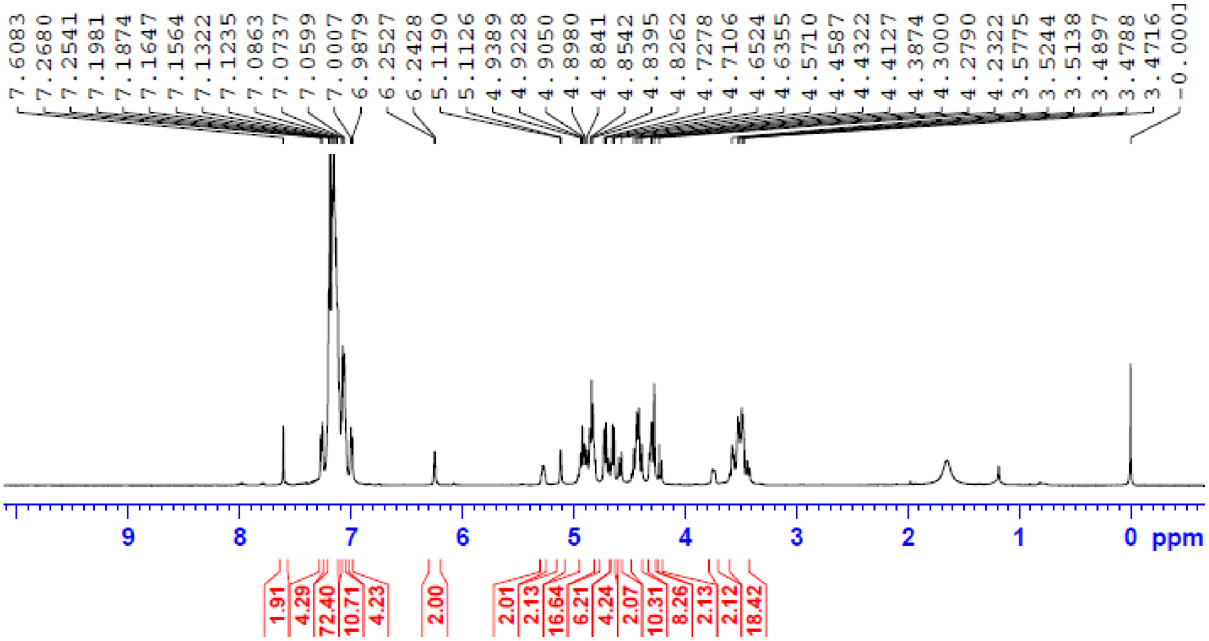

zoom

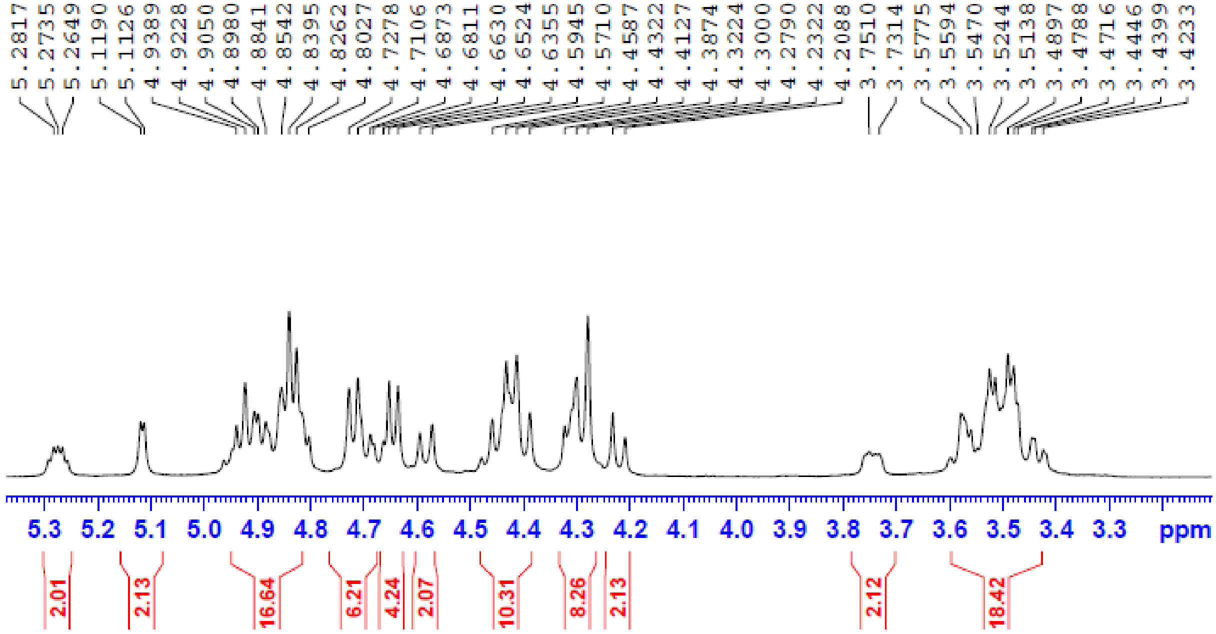

COSY NMR of 15a in $CDCl_3$

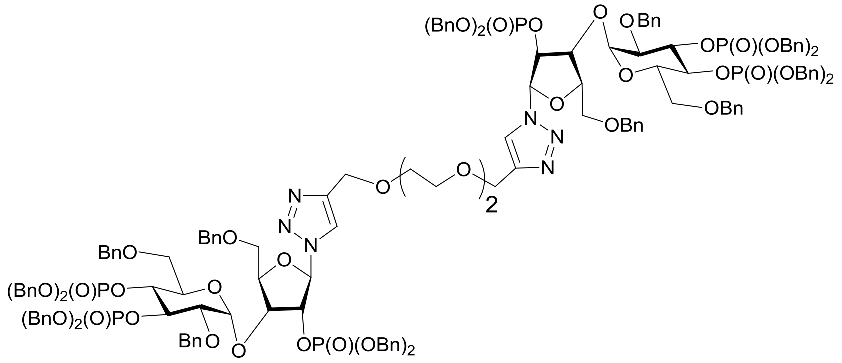

15a

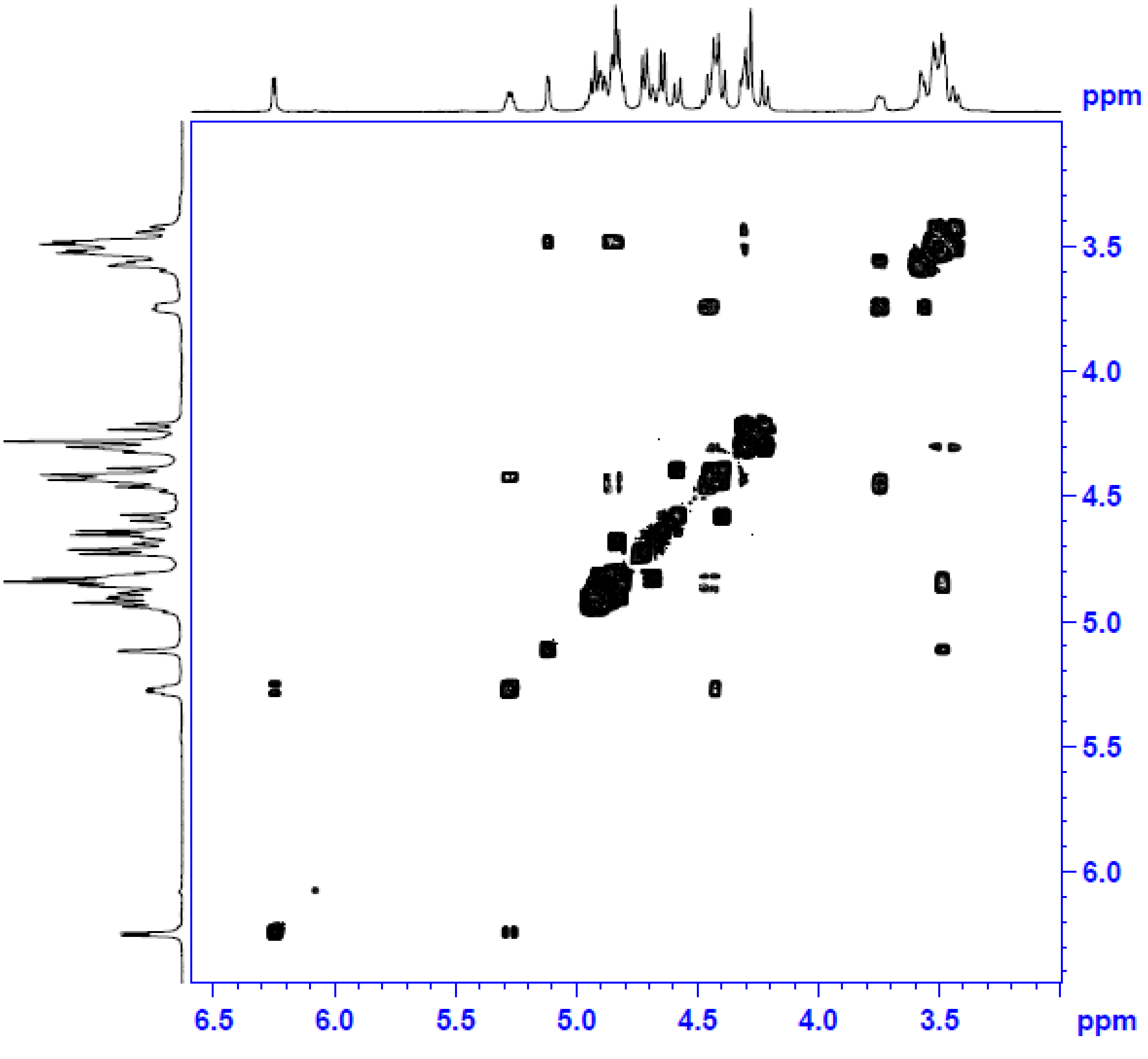

^{13}C NMR of 15a in $CDCl_3$

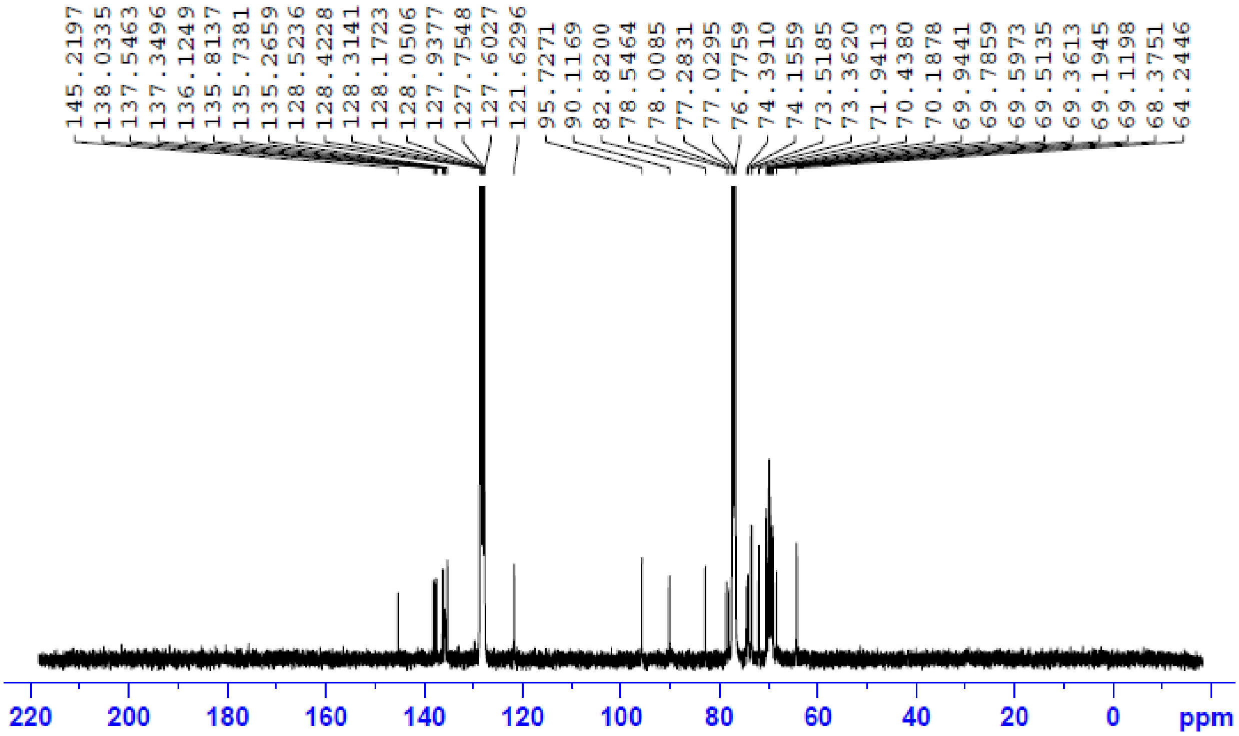

zoom

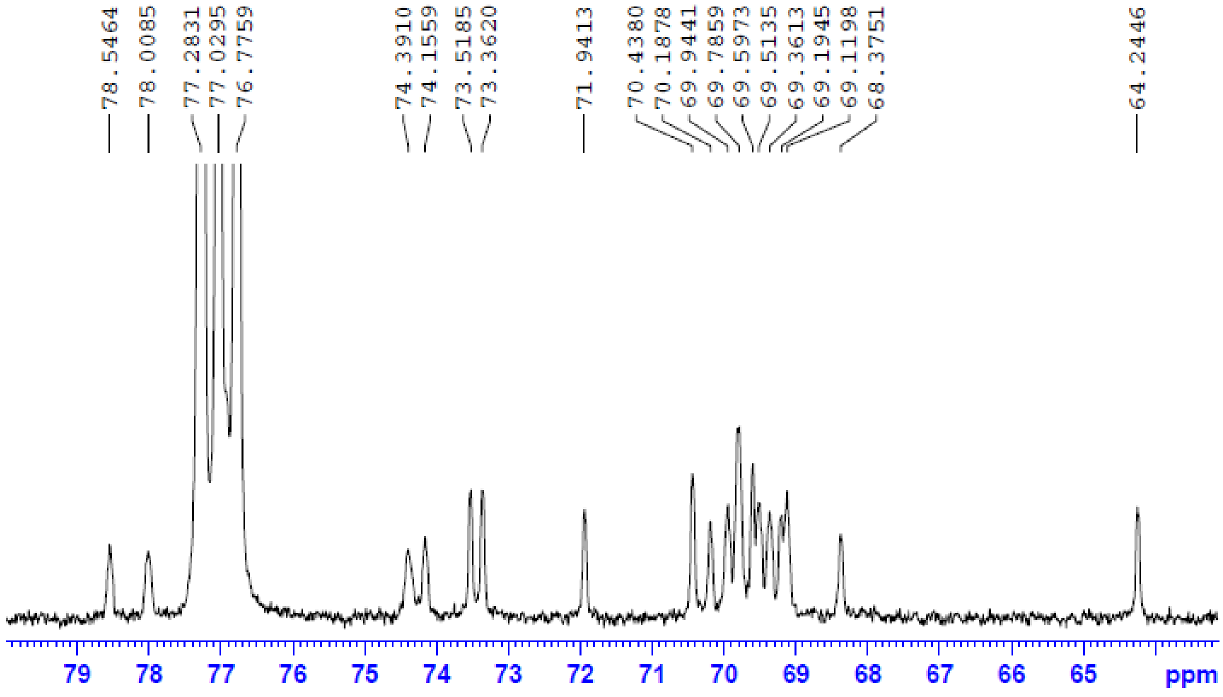

DEPT NMR of 15a in $CDCl_3$

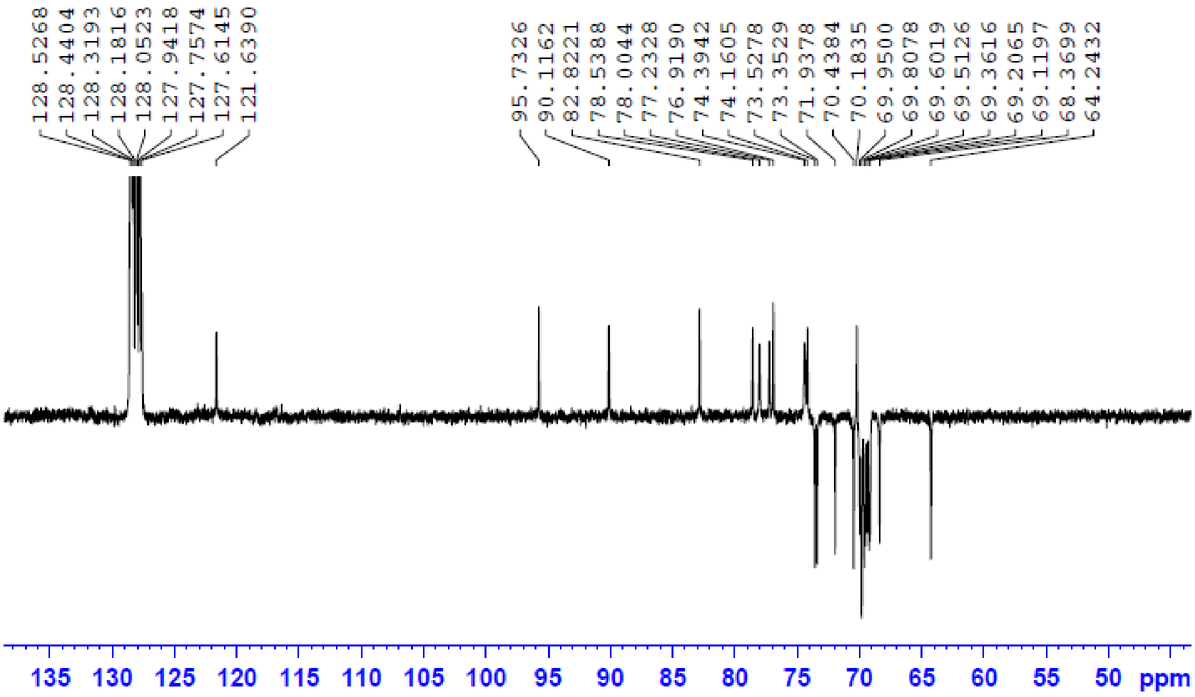

^{31}P NMR of 15a in $CDCl_3$

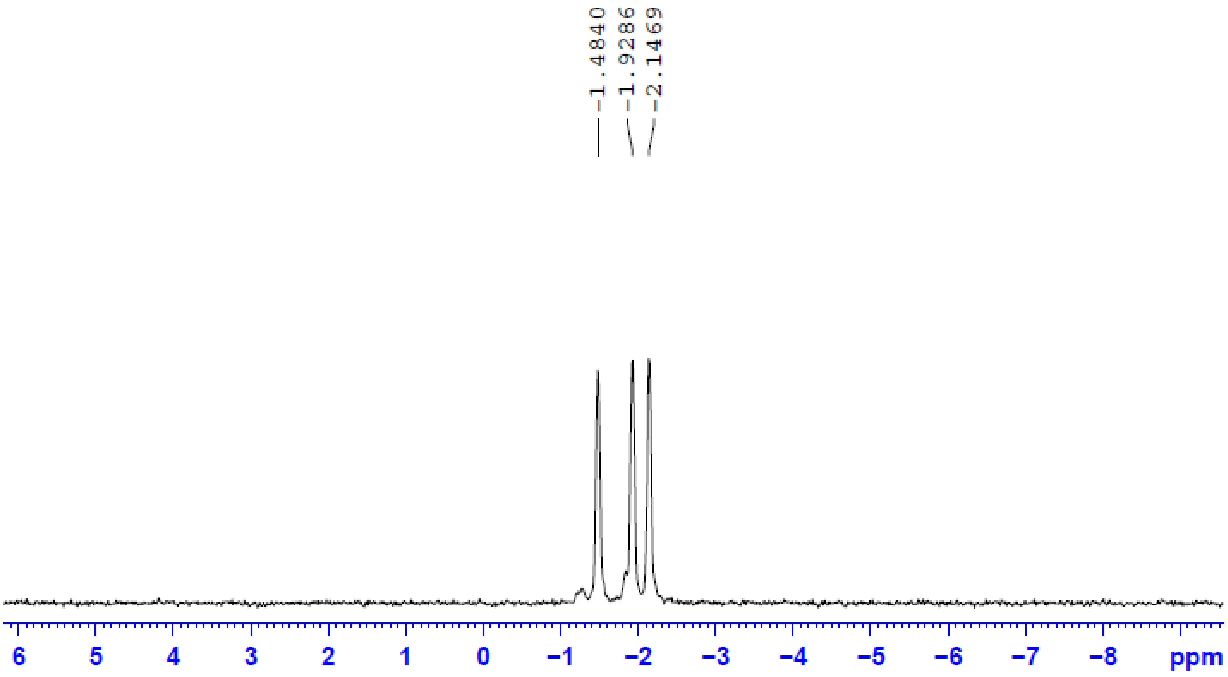

HMBC NMR of 15a in $CDCl_3$

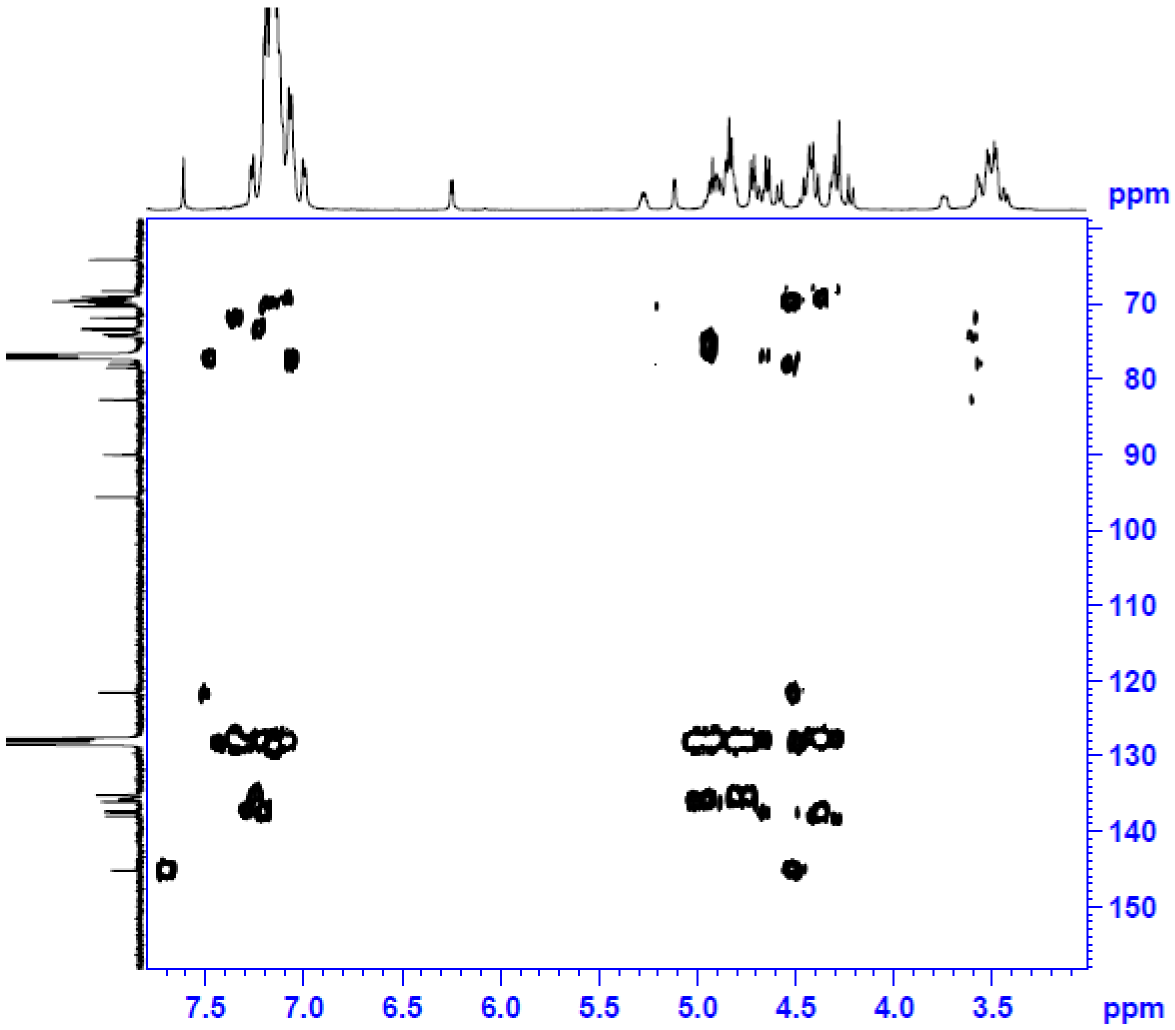

HMQC NMR of 15a in $CDCl_3$

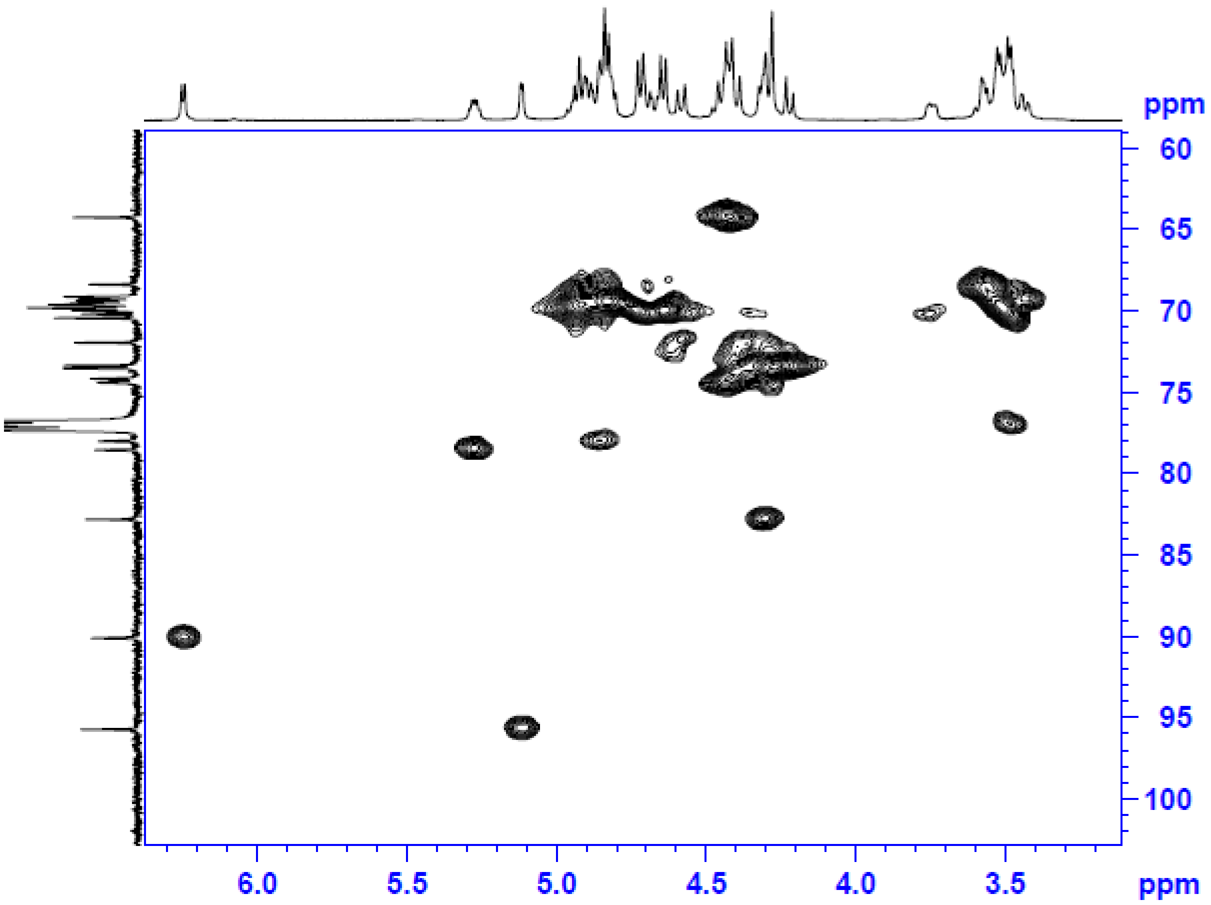

^{1}H NMR of 15b in $CDCl_3$

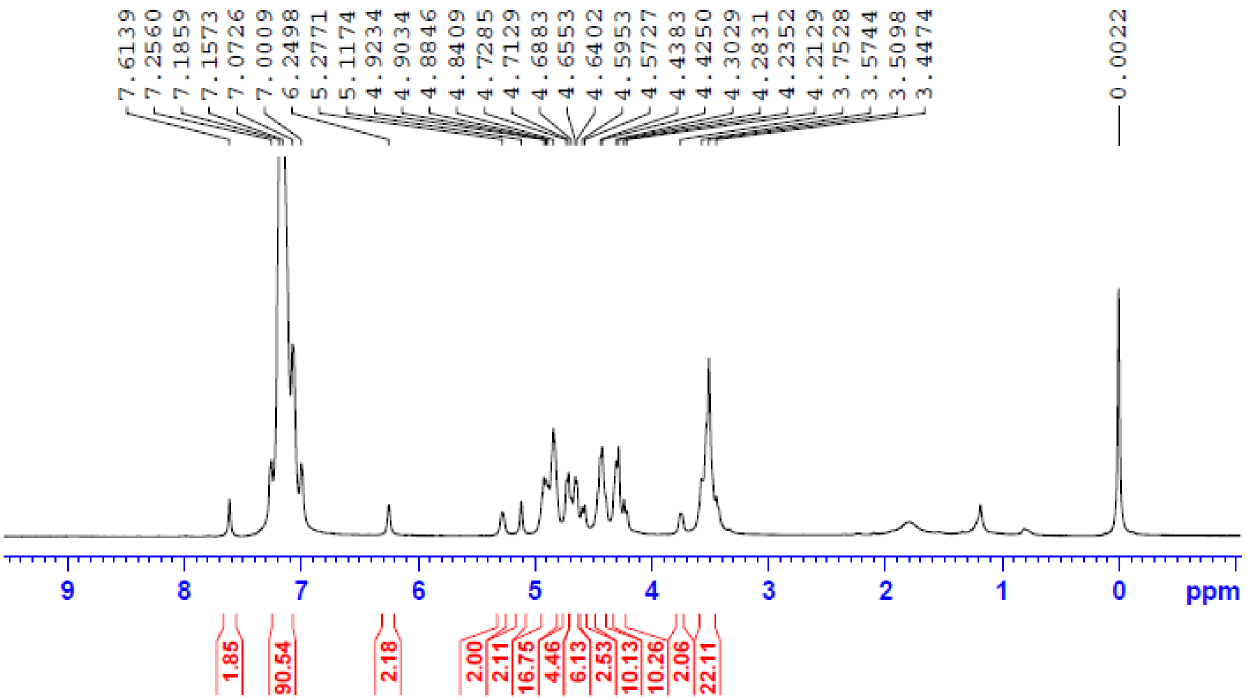

zoom

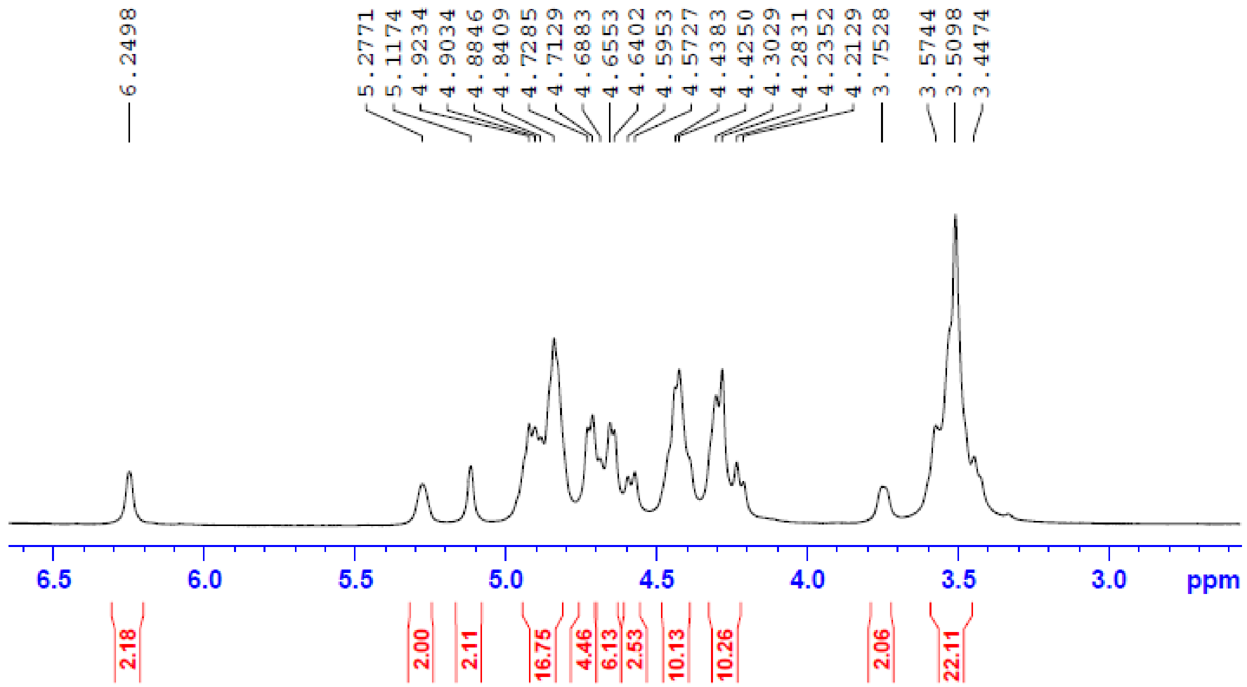

COSY NMR of 15b in $CDCl_3$

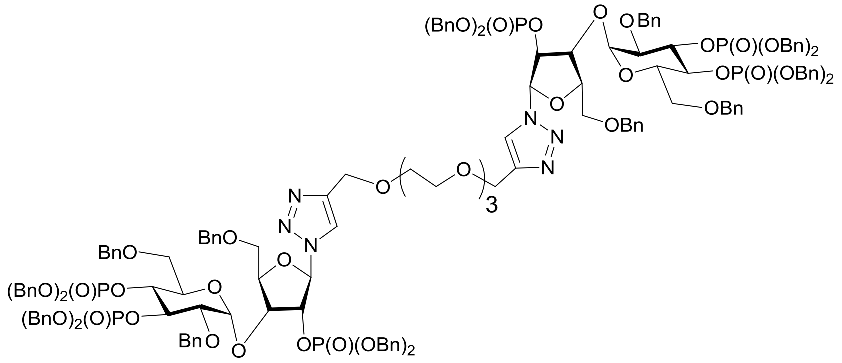

15b

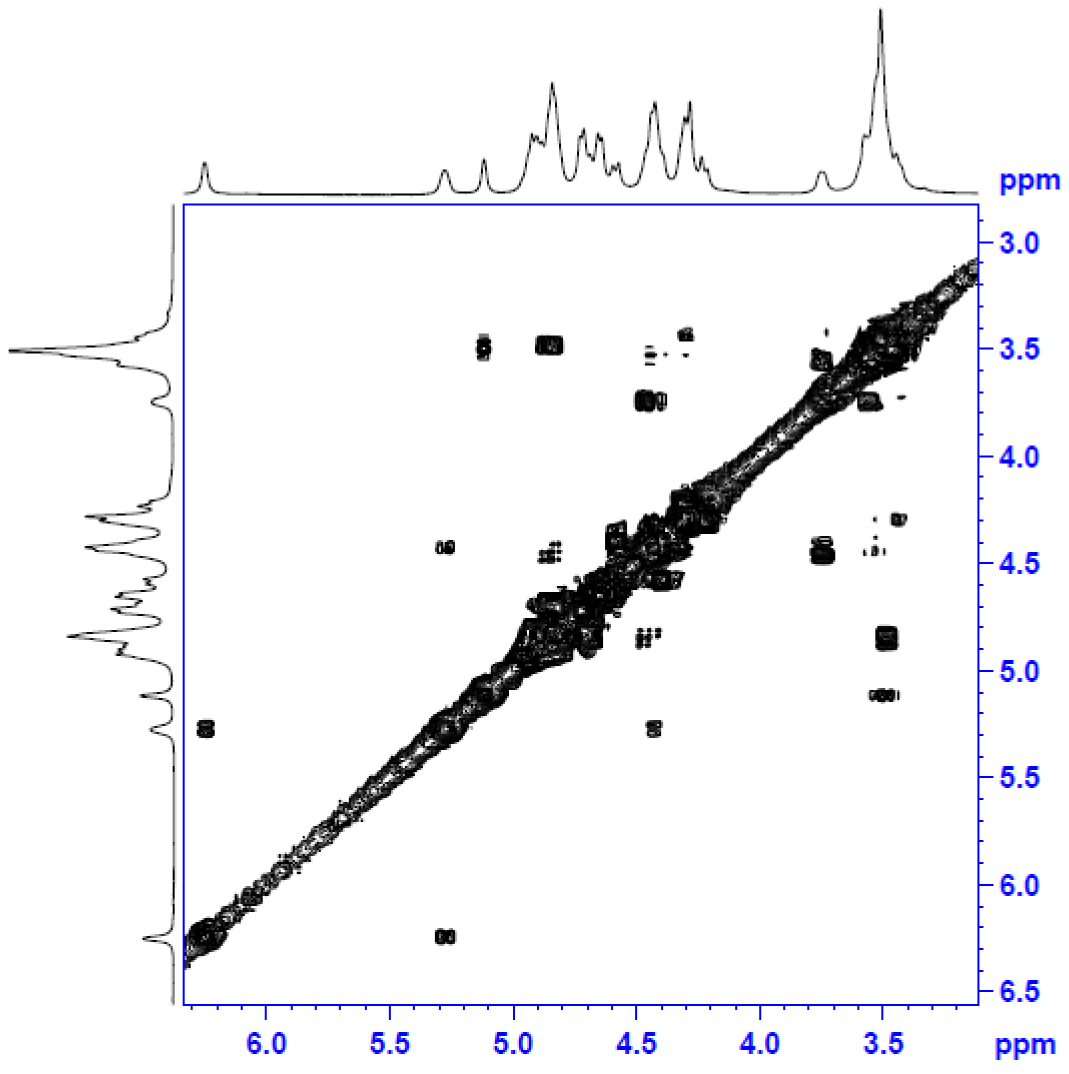

^{13}C NMR of 15b in $CDCl_3$

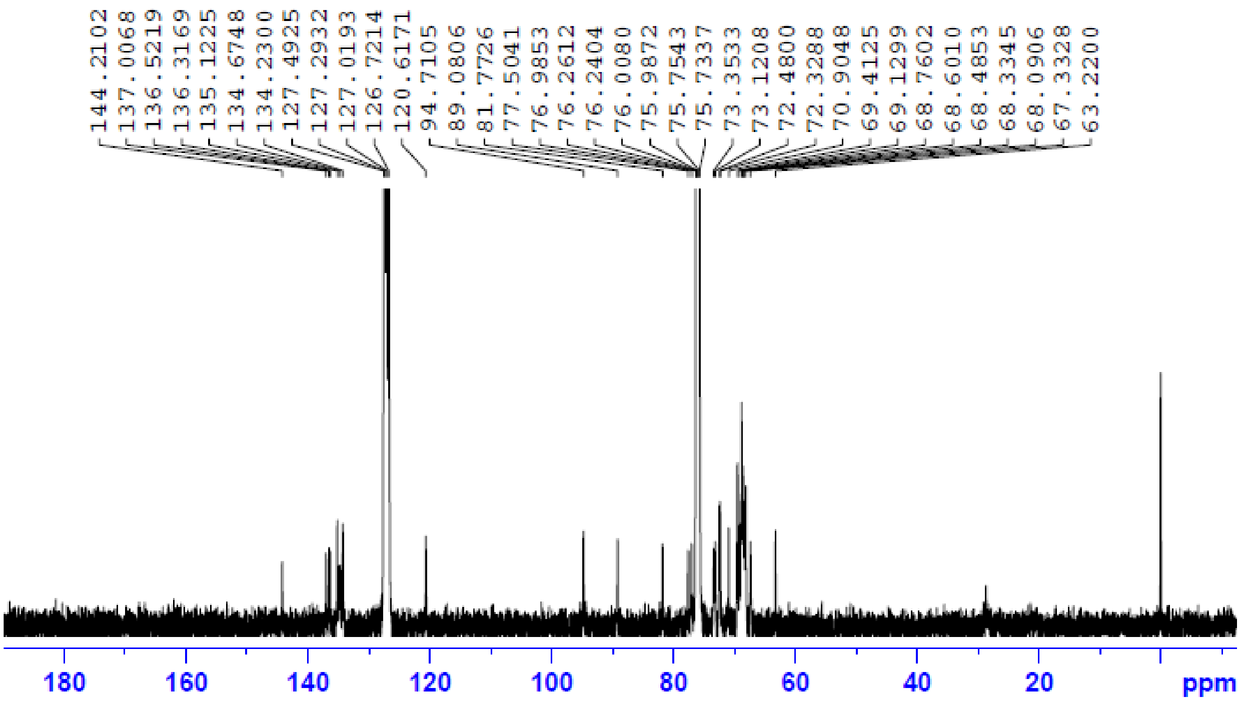

zoom

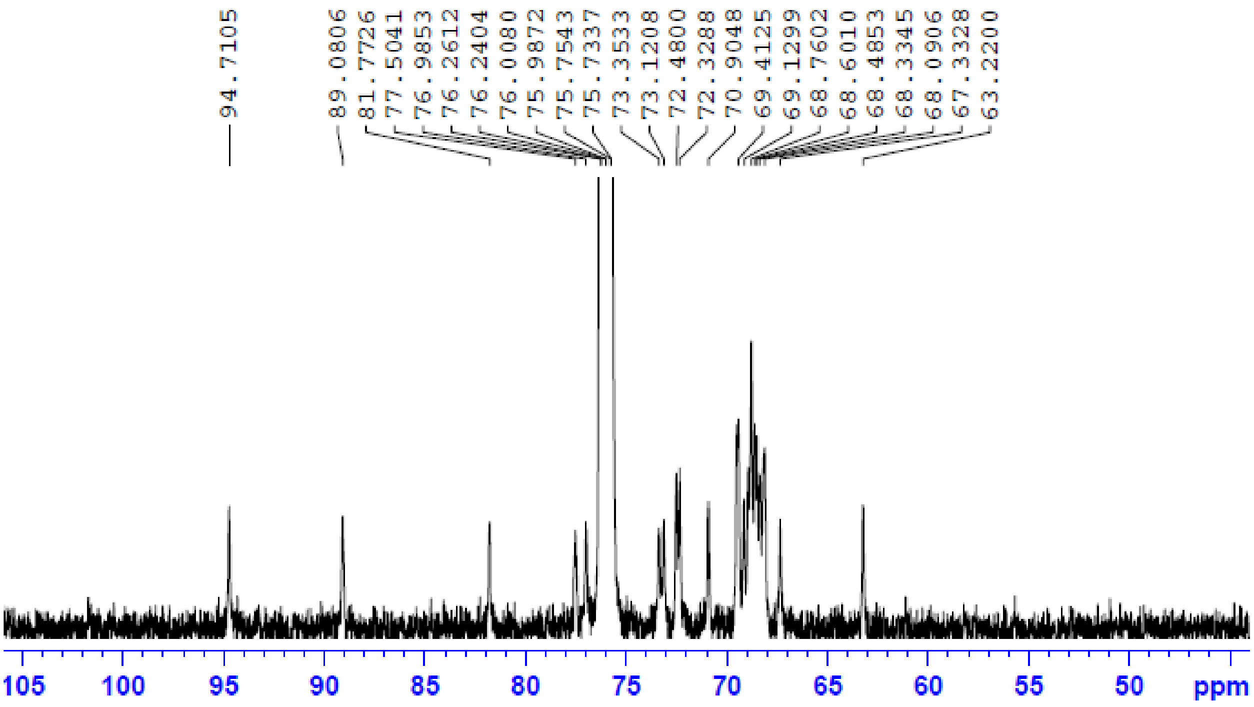

DEPT NMR of 15b in $CDCl_3$

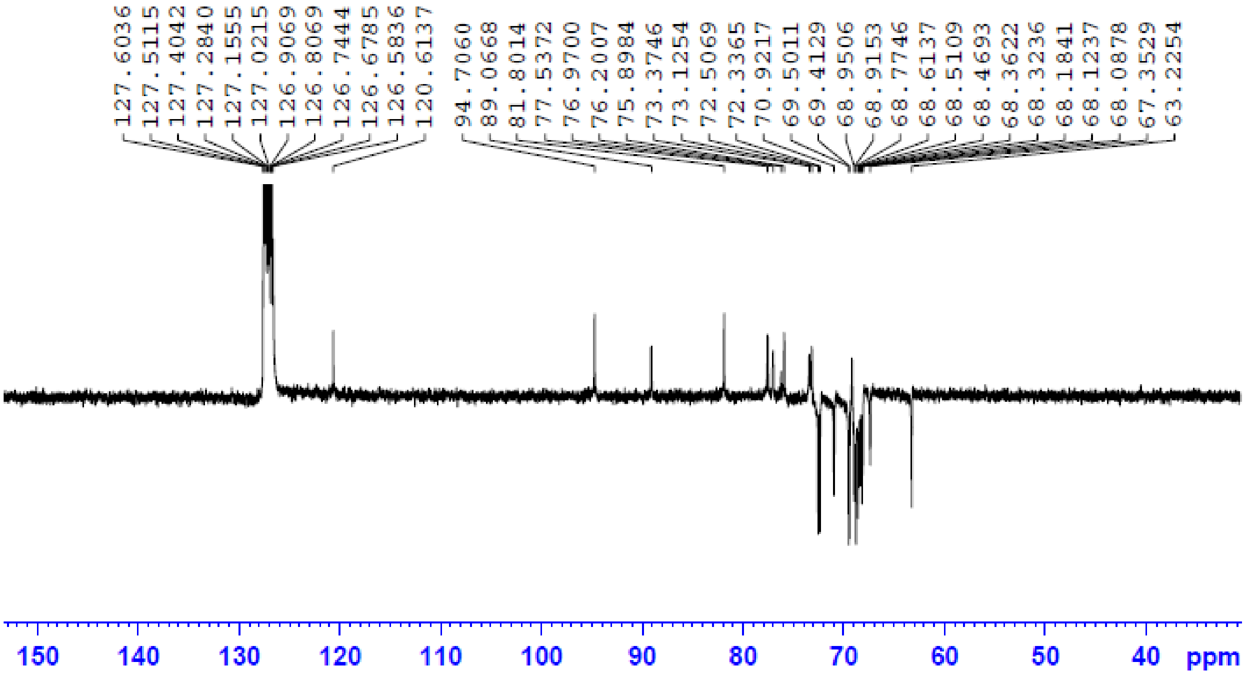

^{31}P NMR of 15b in $CDCl_3$

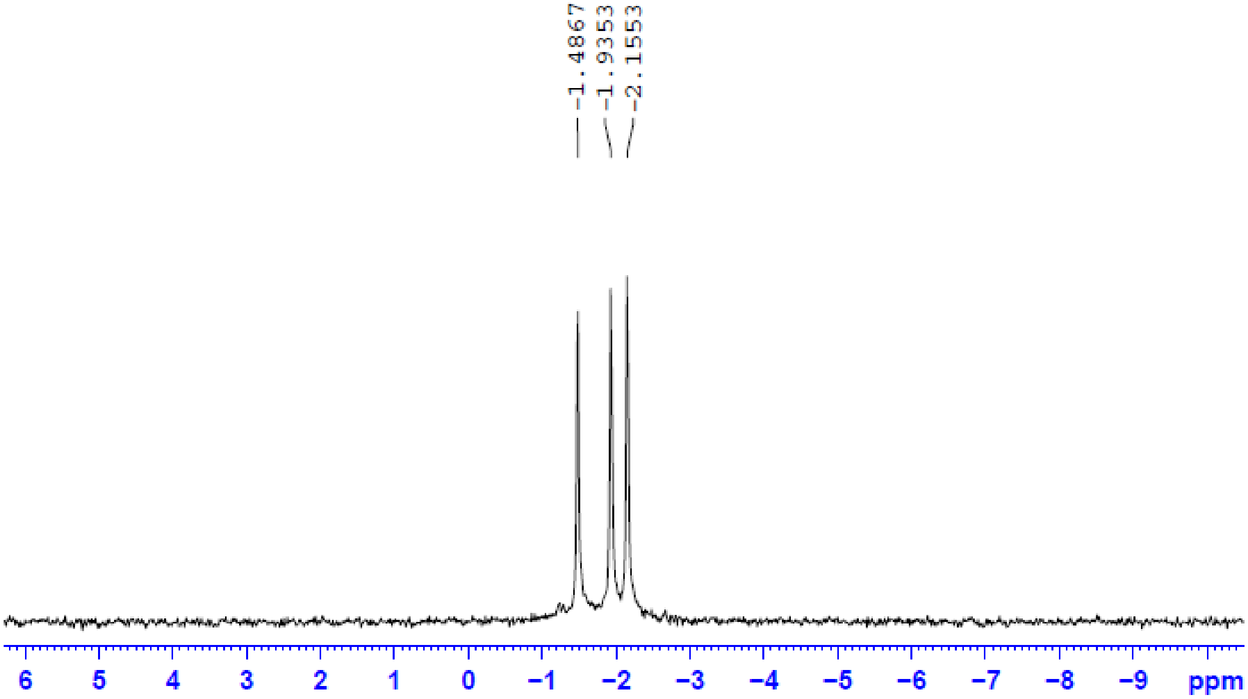

HMBC NMR of 15b in $CDCl_3$

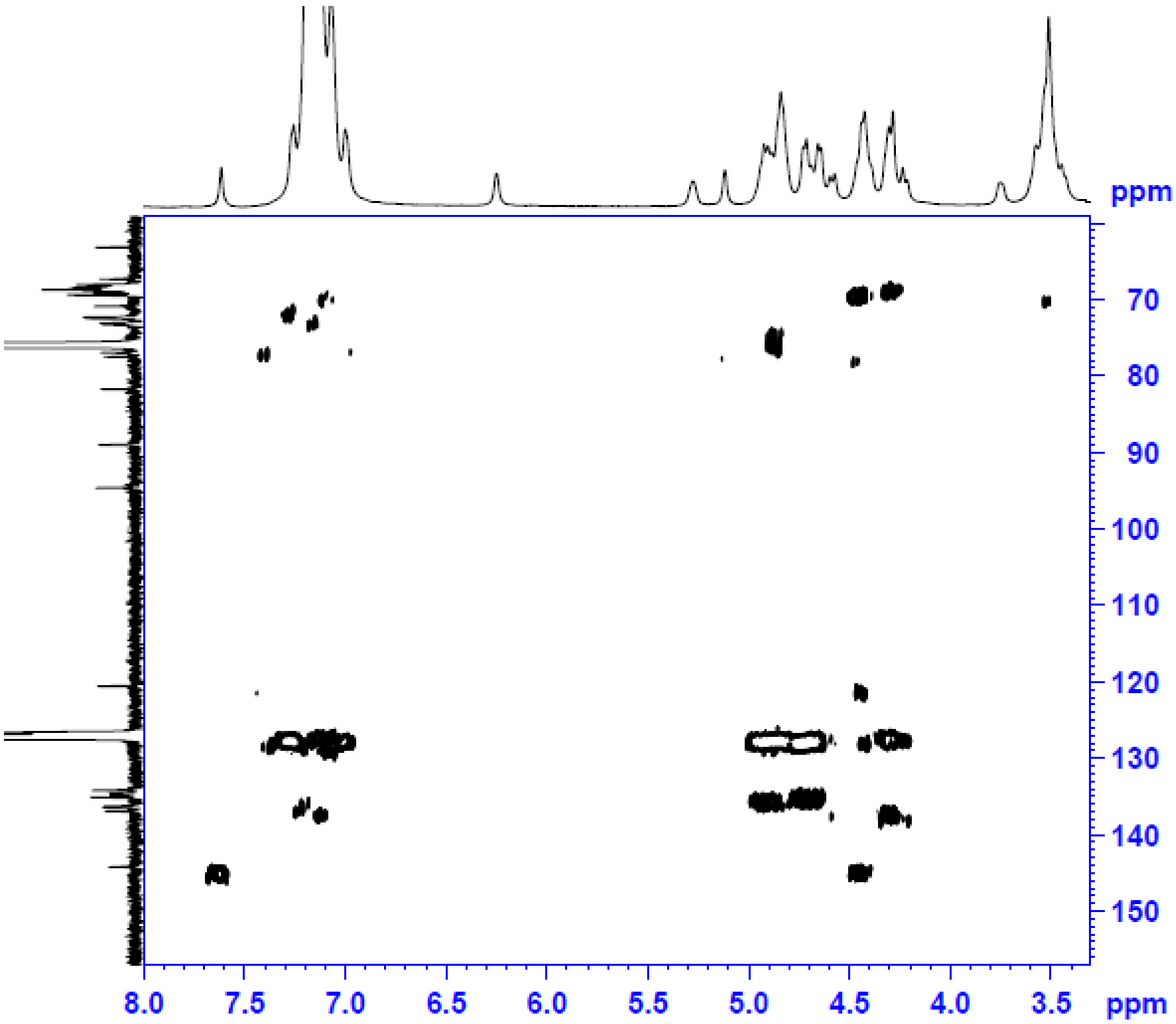

HMQC NMR of 15b in $CDCl_3$

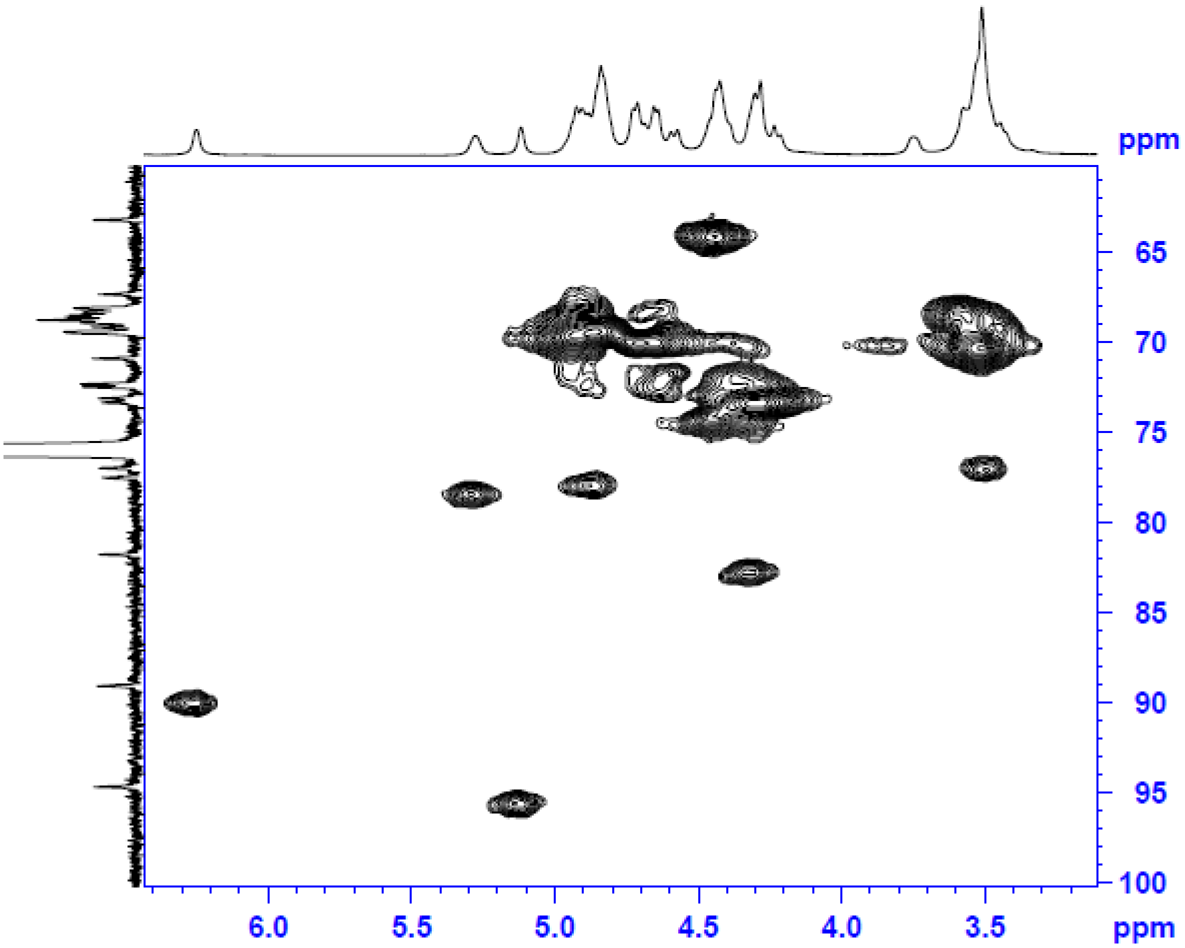

^{1}H NMR of 15c in $CDCl_3$

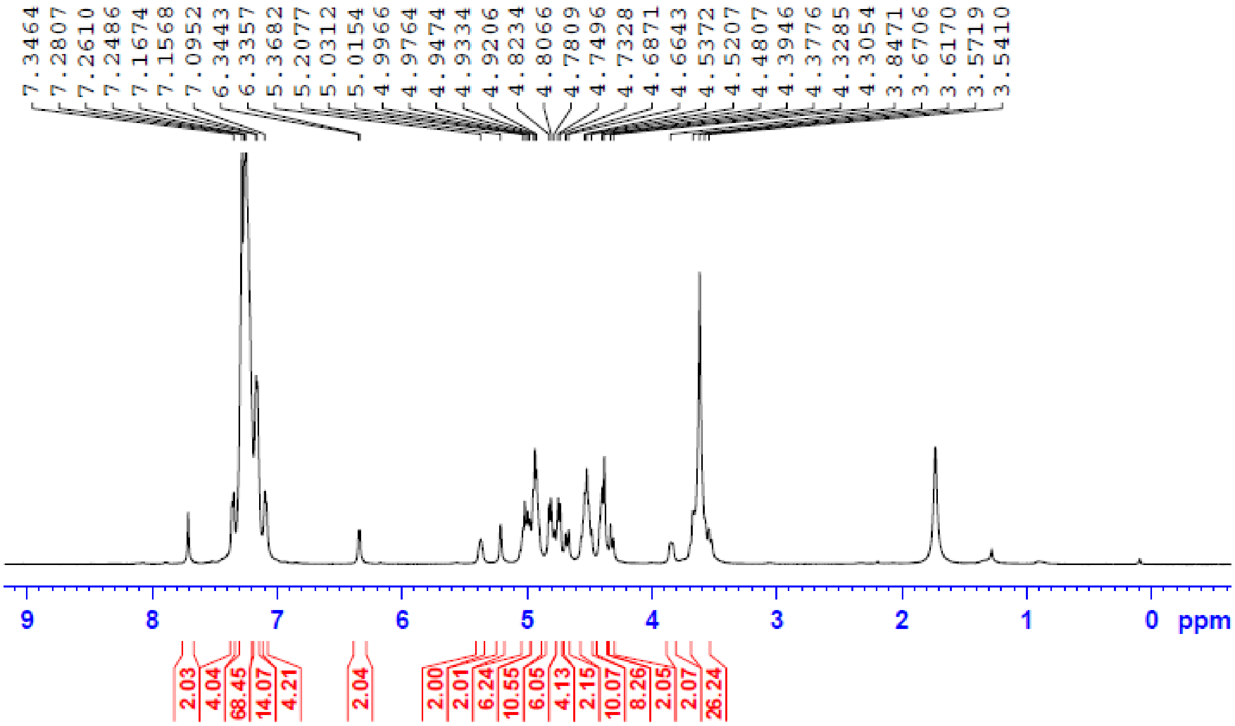

zoom

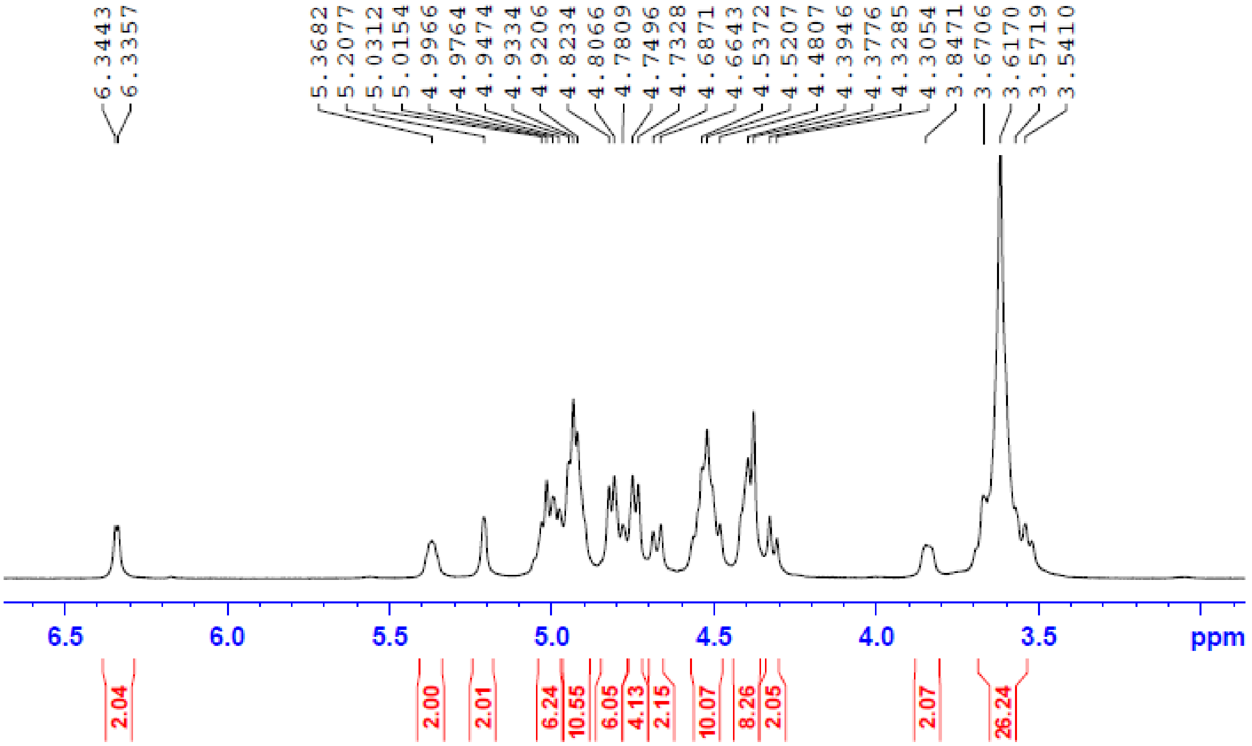

COSY NMR of 15c in $CDCl_3$

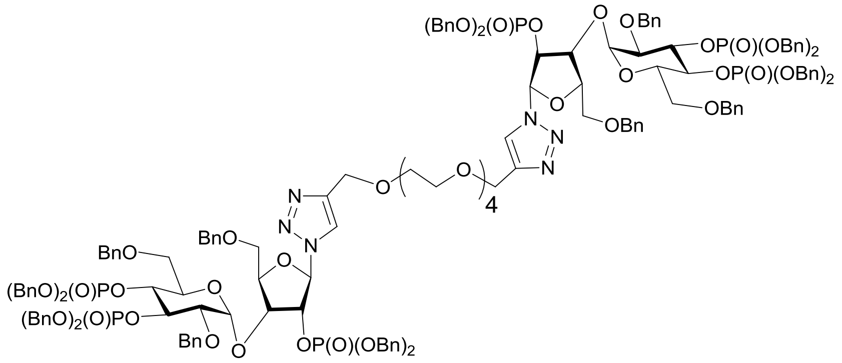

15c

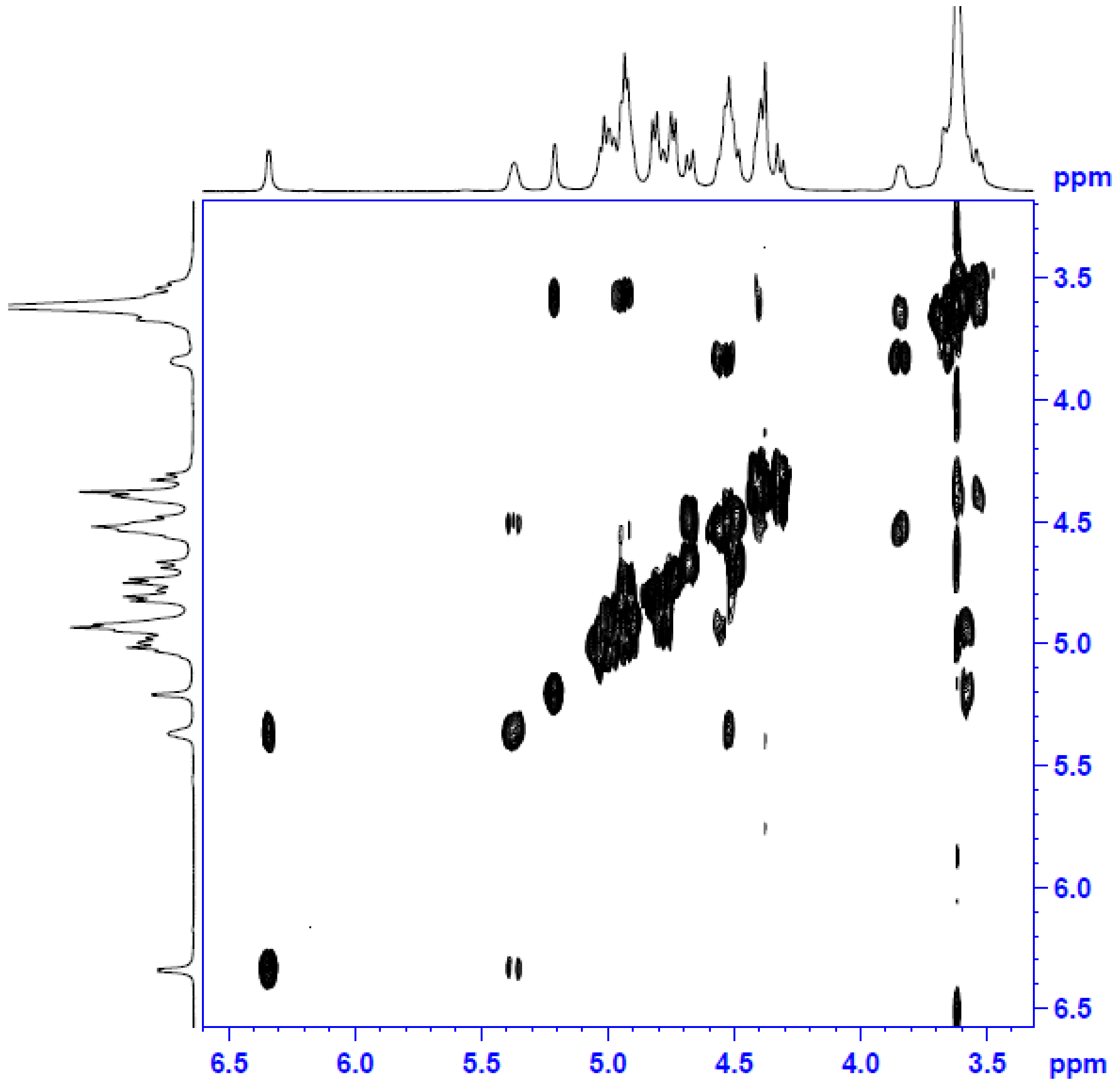

^{13}C NMR of 15c in $CDCl_3$

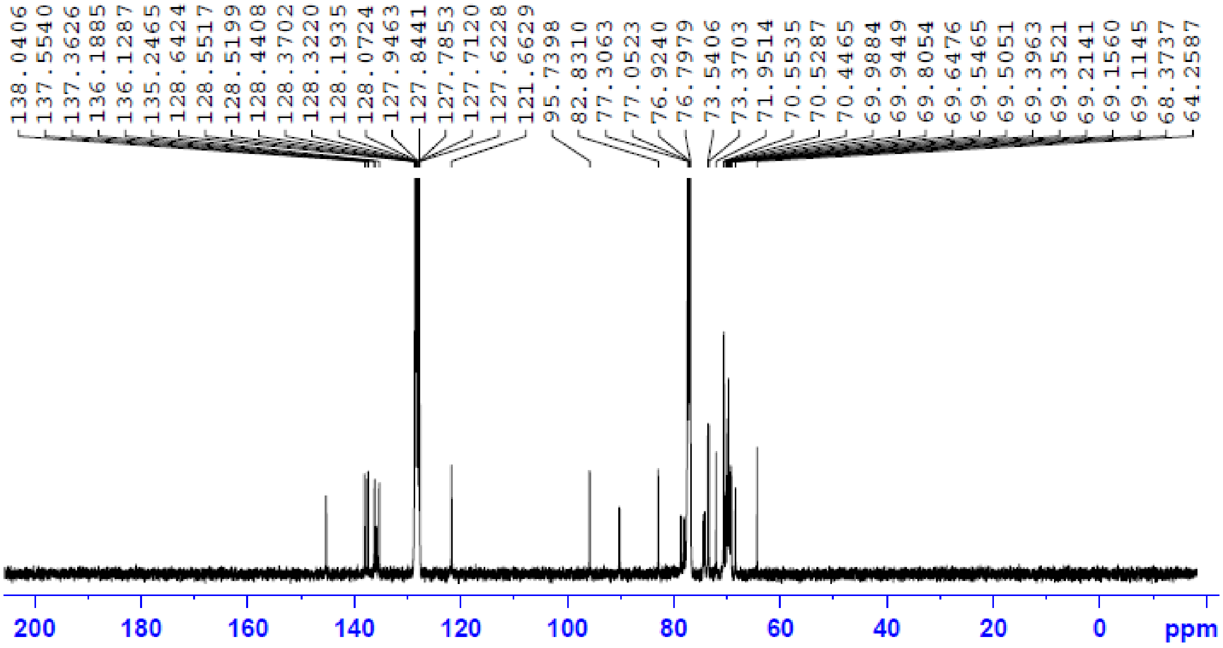

zoom

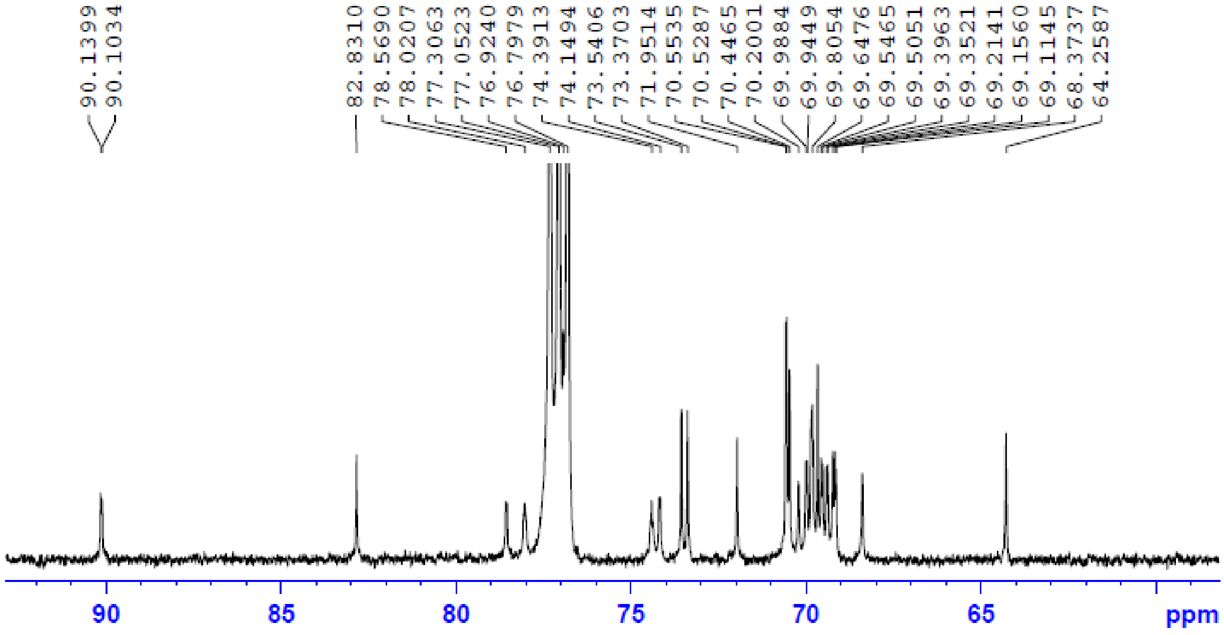

DEPT NMR of 15c in $CDCl_3$

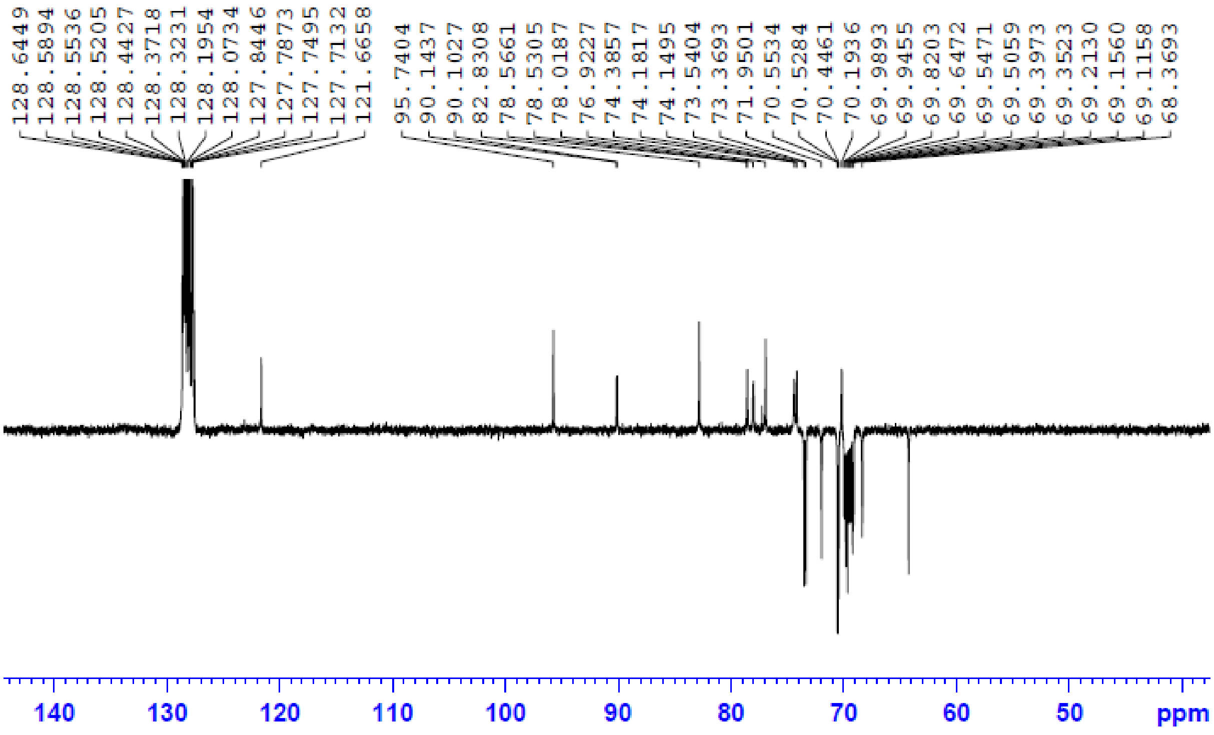

^{31}P NMR of 15c in $CDCl_3$

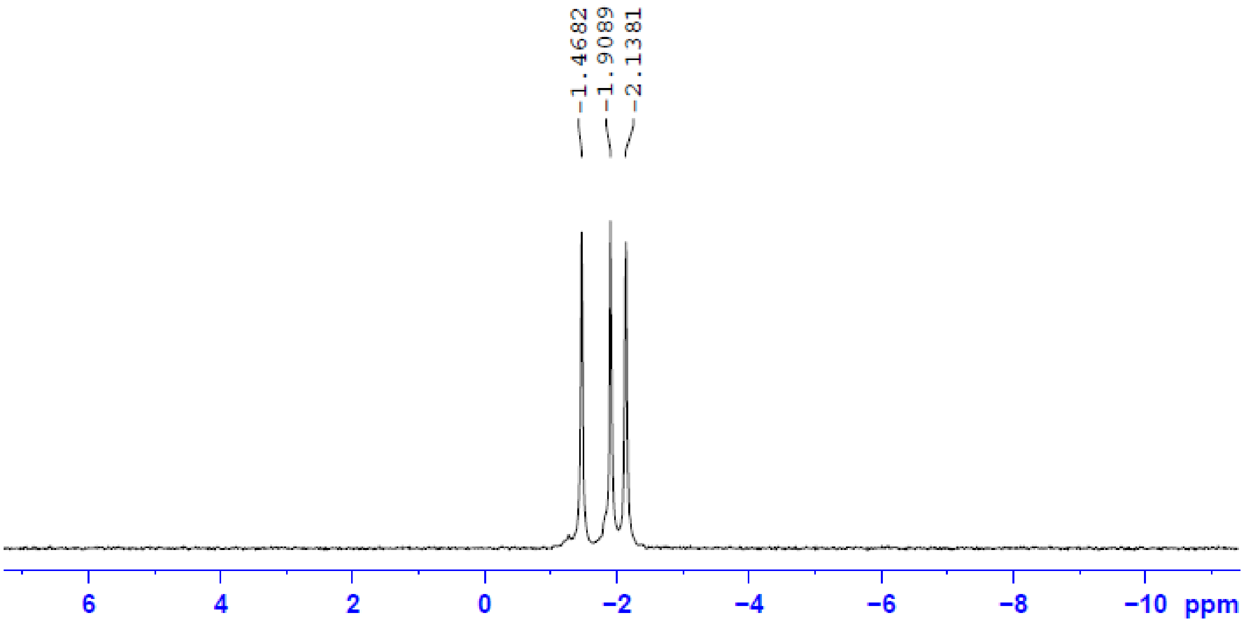

HMBC NMR of 15c in $CDCl_3$

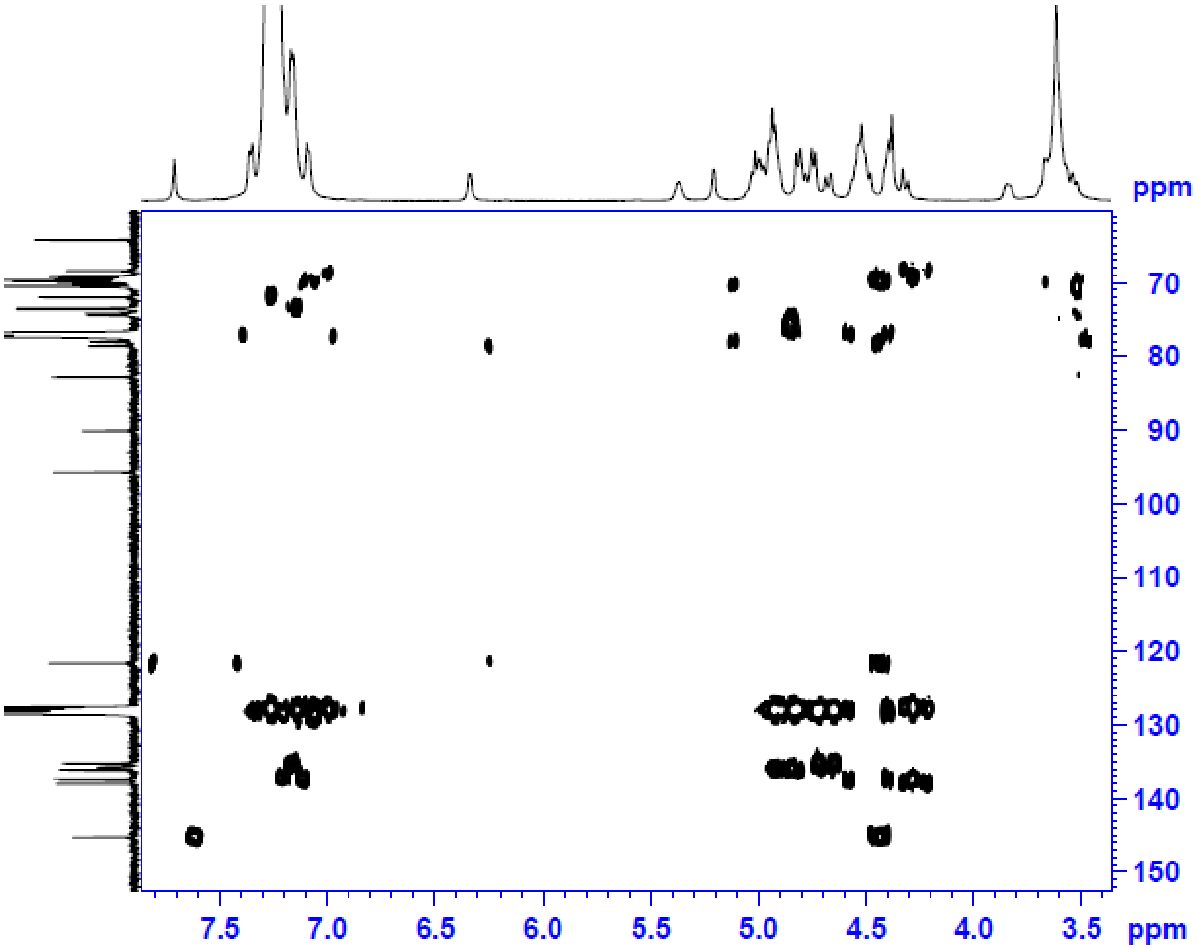

HMQC NMR of 15c in $CDCl_3$

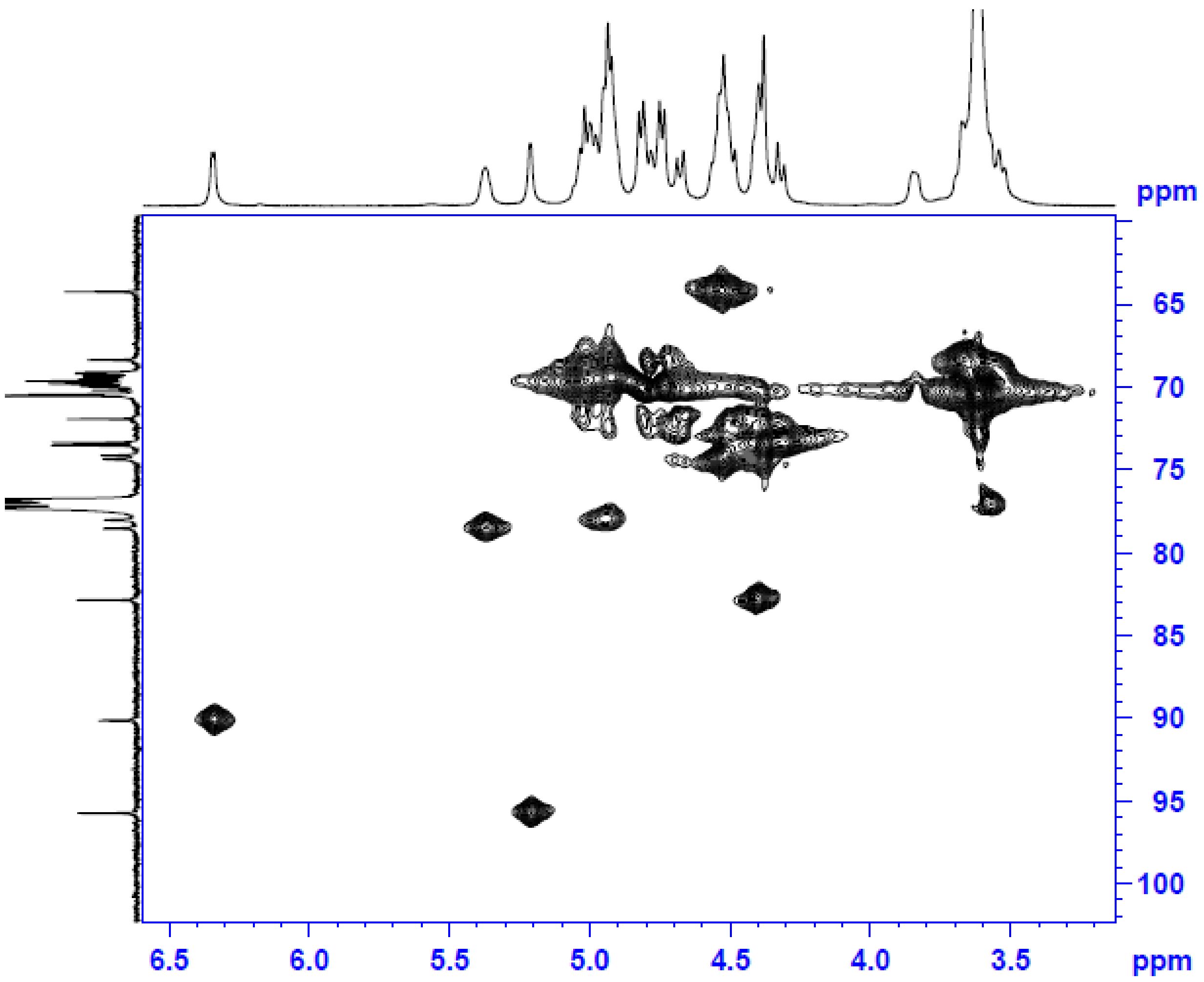

^1H NMR of 15d in $CDCl_3$

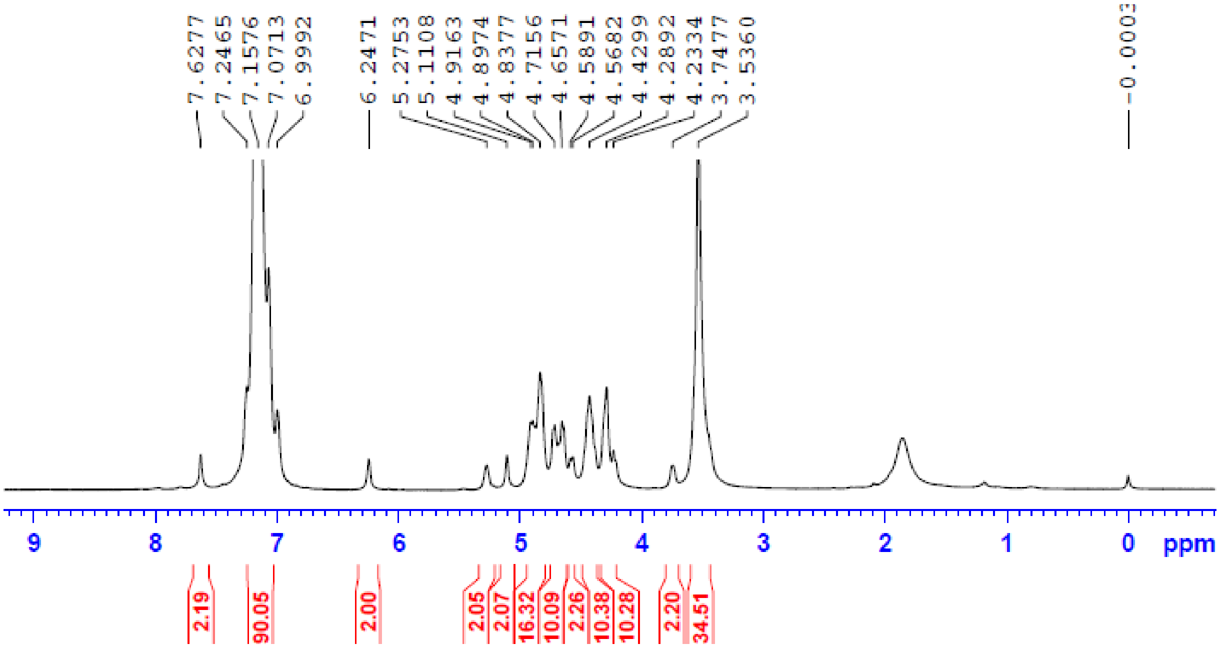

^{31}P NMR of 15d in $CDCl_3$

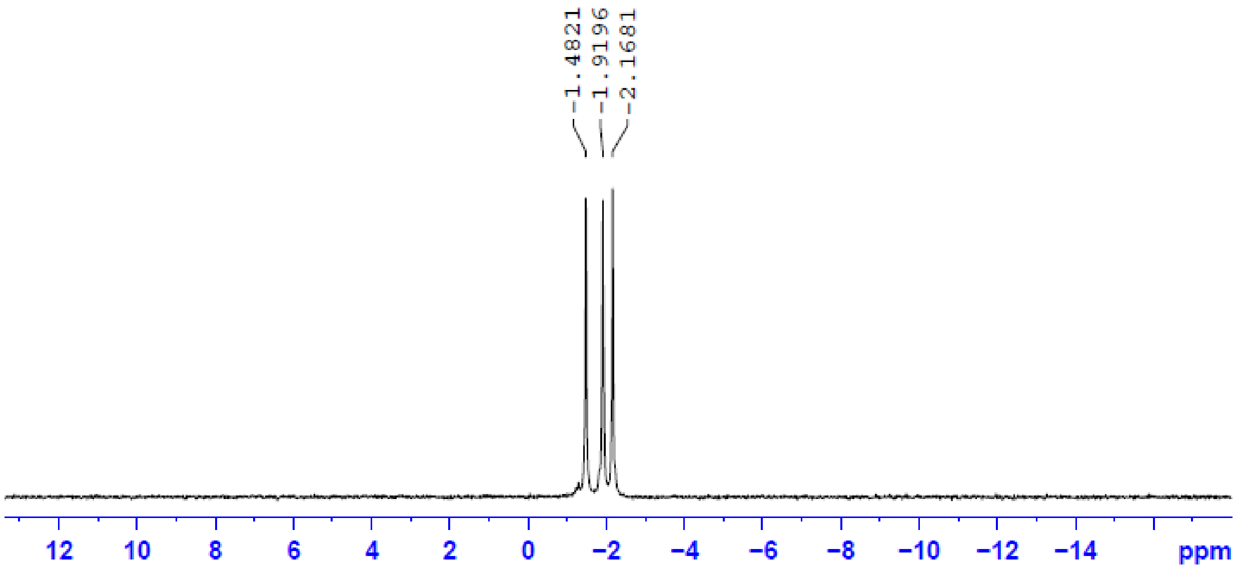

COSY NMR of 15d in $CDCl_3$

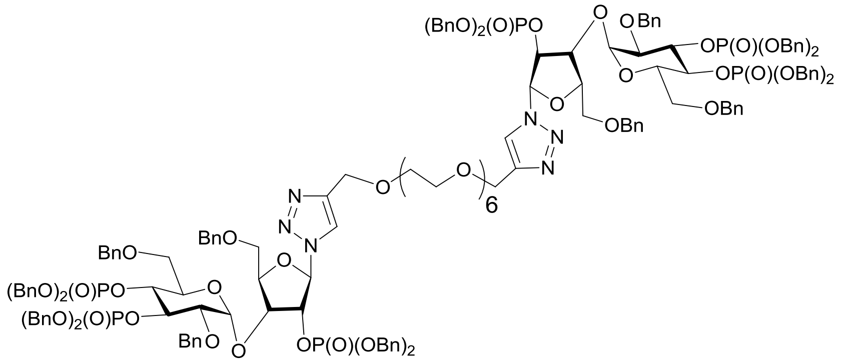

15d

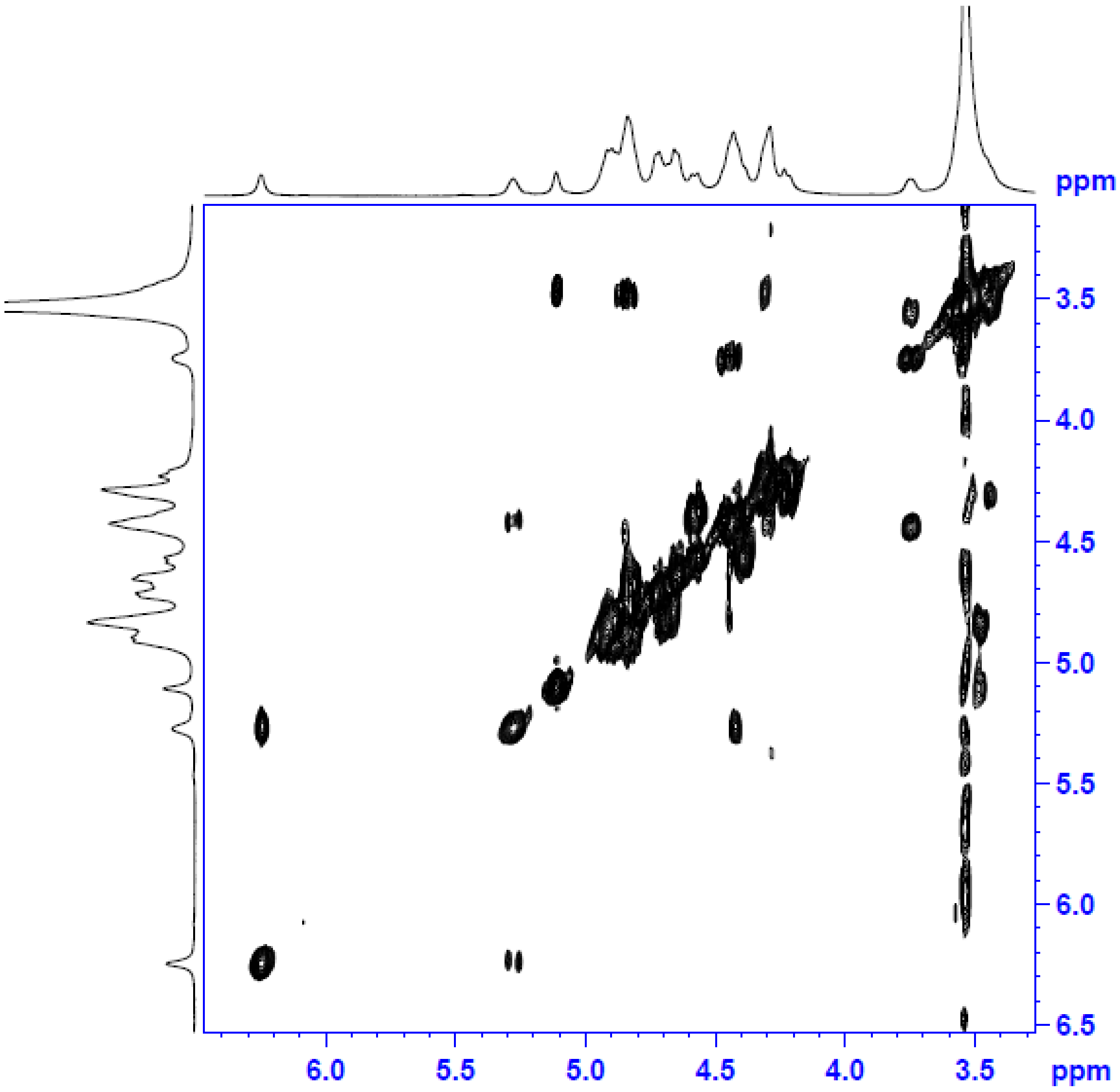

^{13}C NMR of 15d in $CDCl_3$

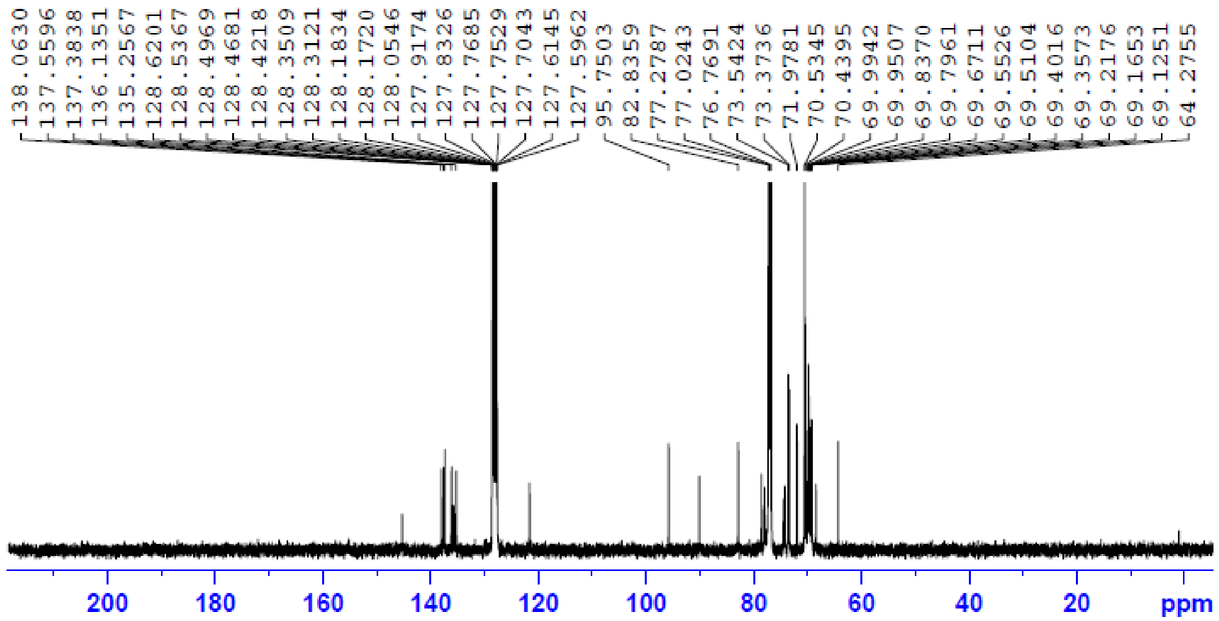

zoom

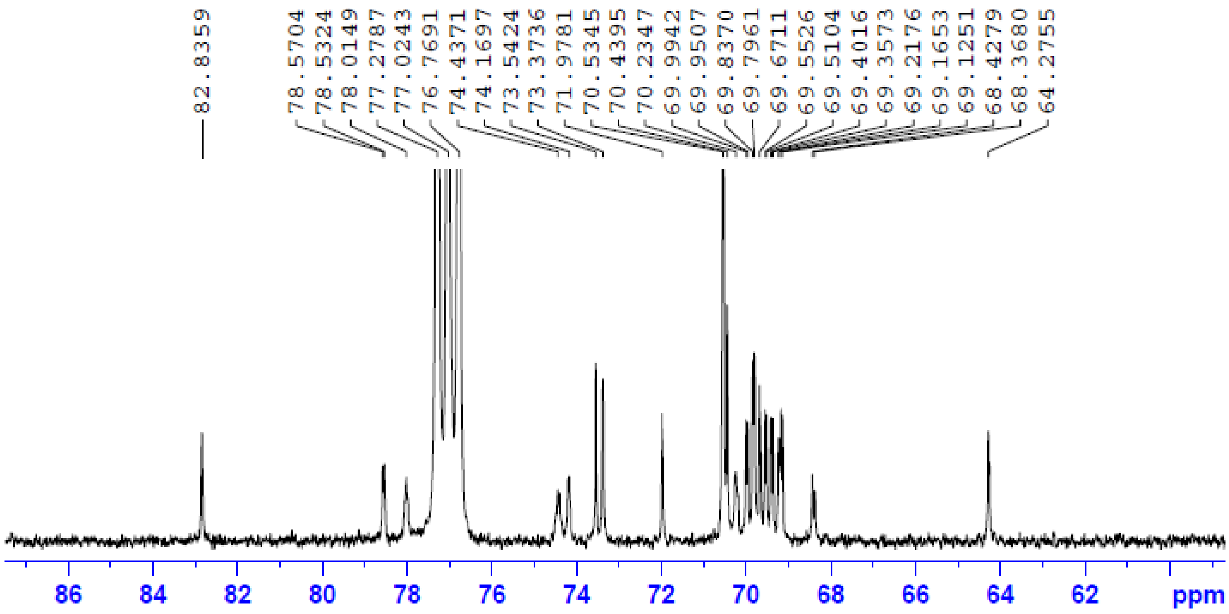

DEPT NMR of 15d in $CDCl_3$

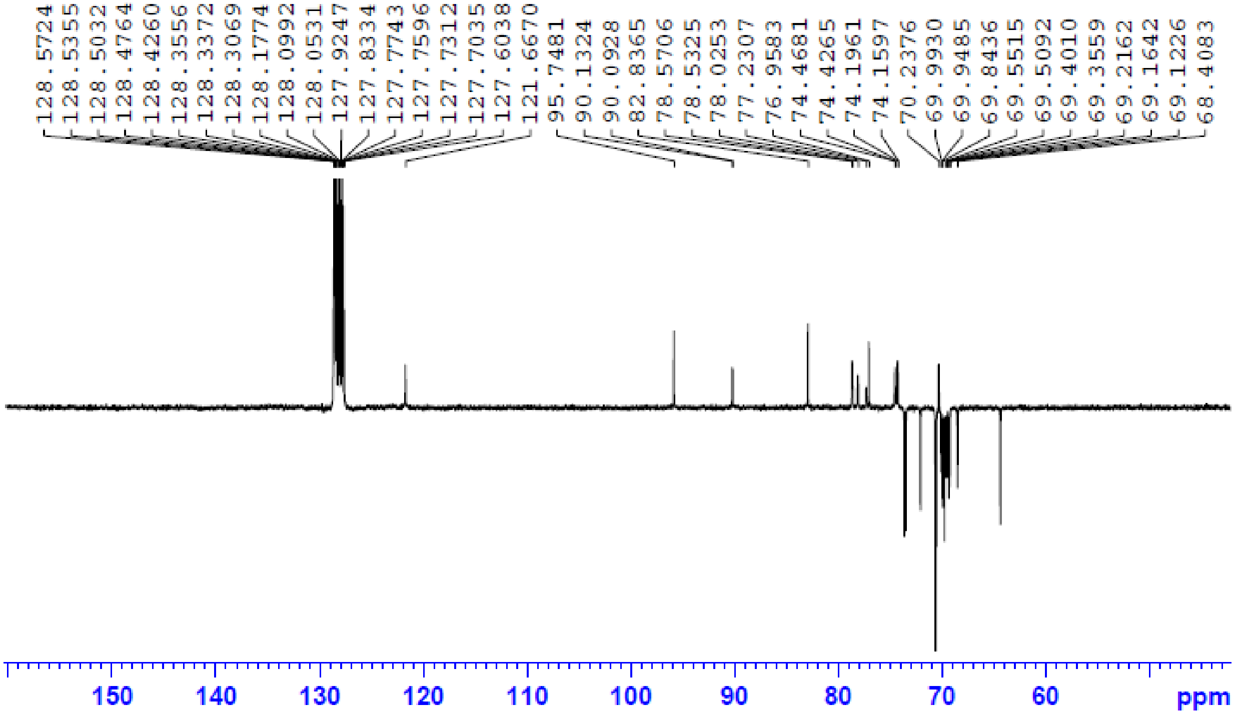

HMBC NMR of 15d in $CDCl_3$

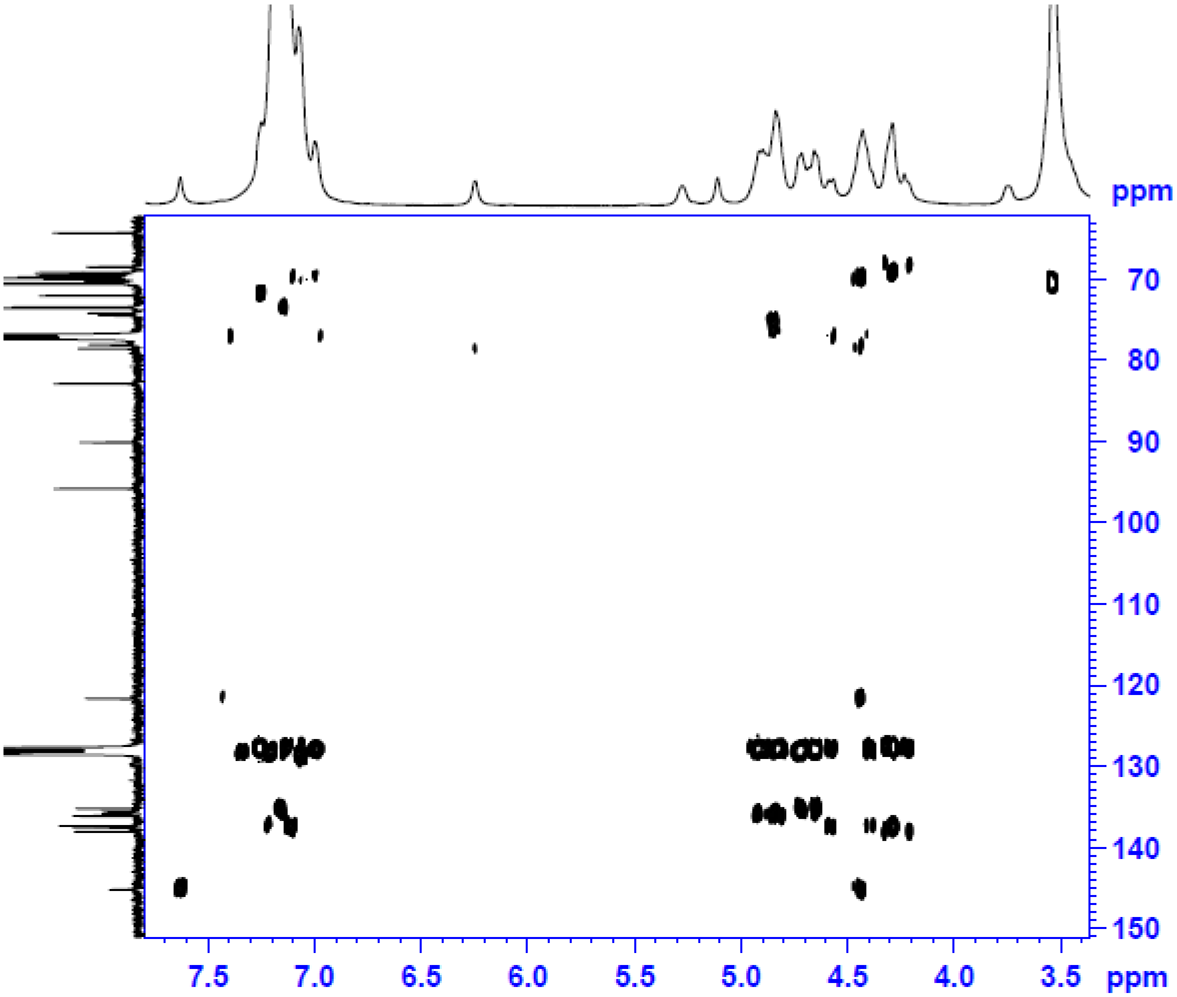

HMQC NMR of 15d in $CDCl_3$

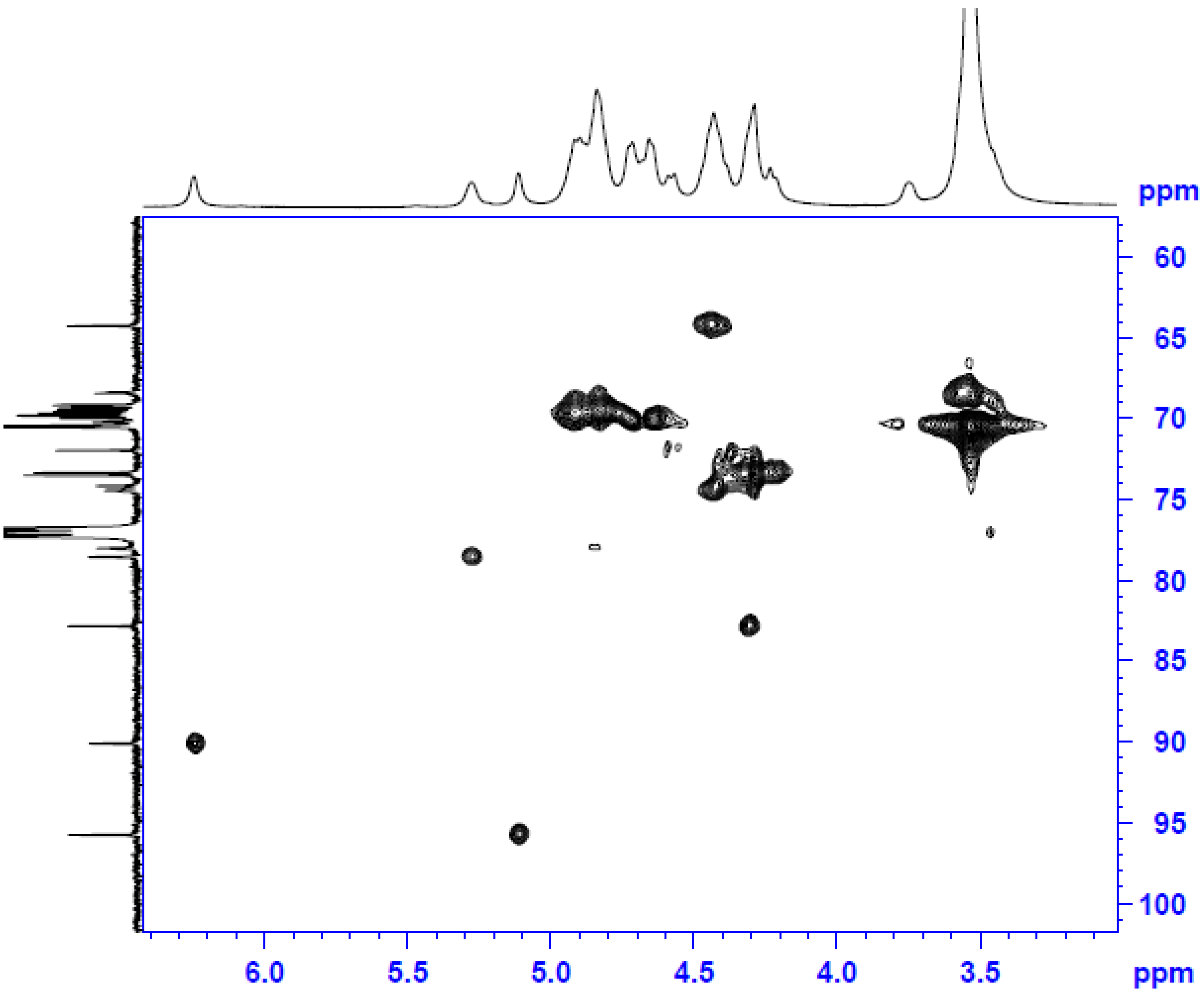

^1H NMR of 12a in D_2O

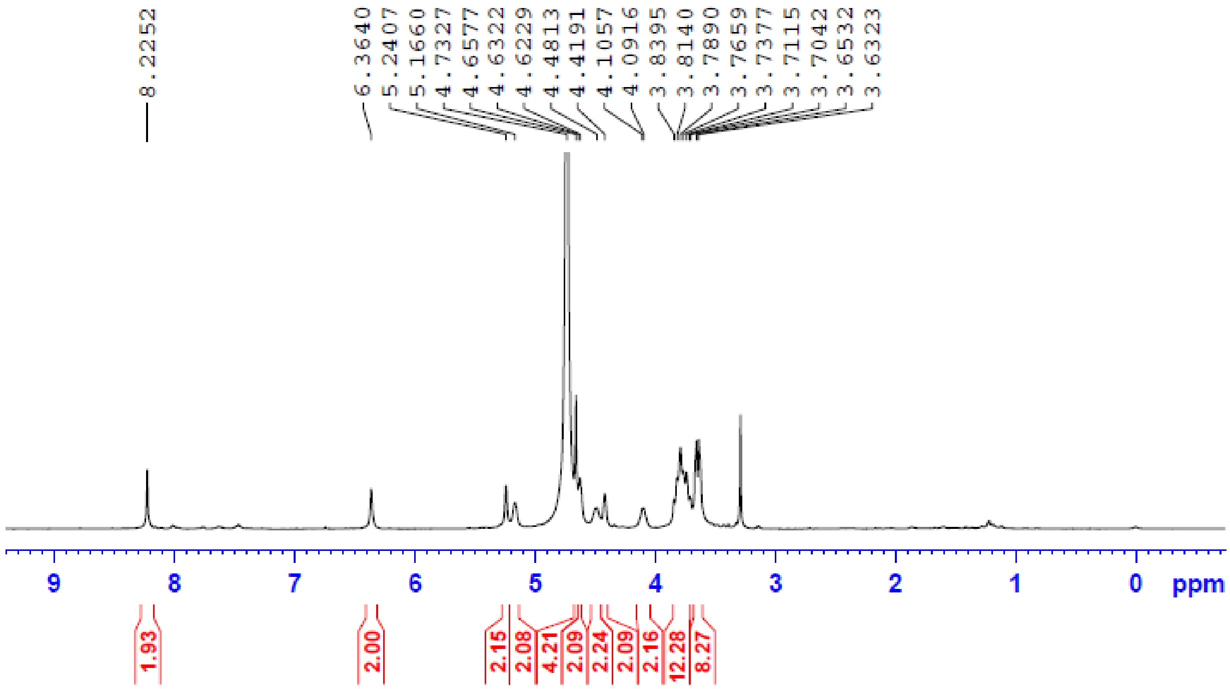

zoom

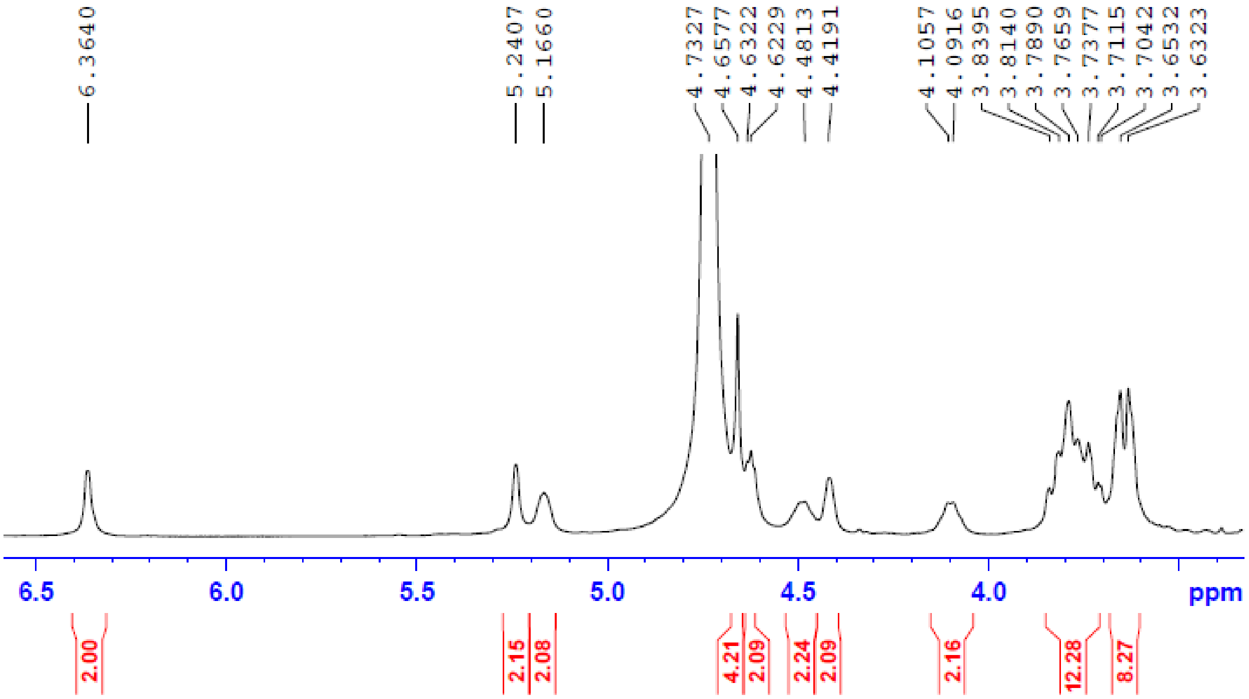

COSY NMR of 12a in D_2O

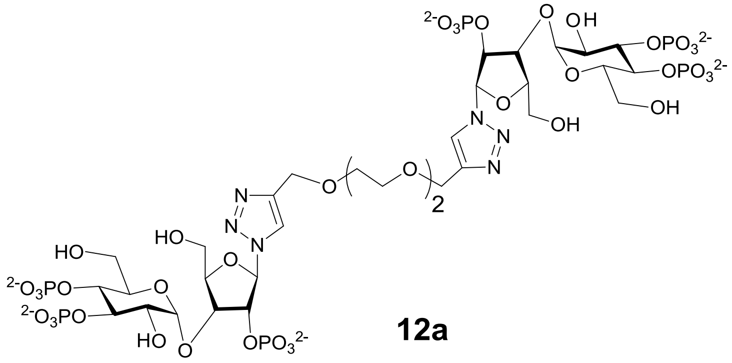

12a

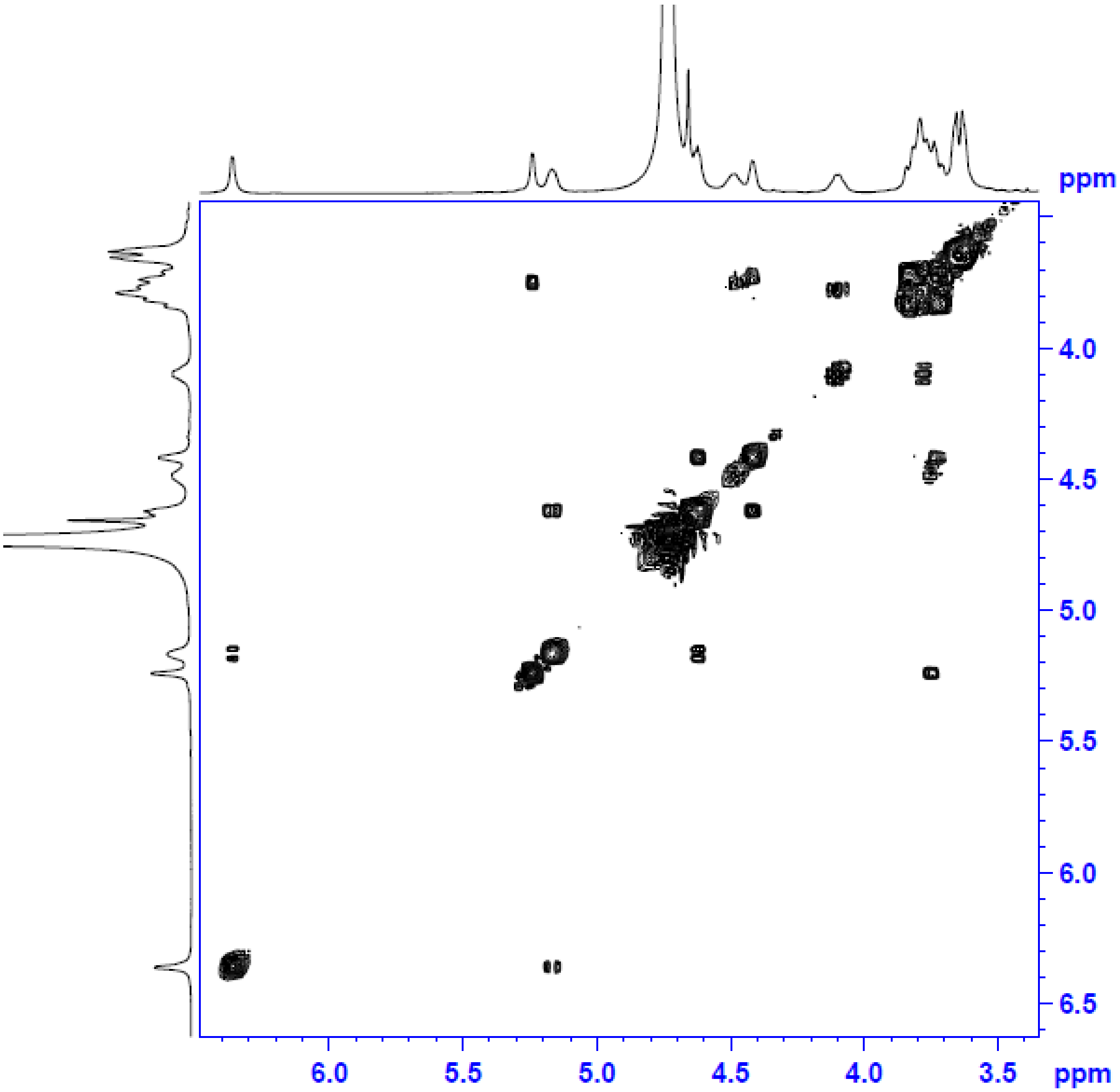

^{13}C NMR of 12a in D_2O

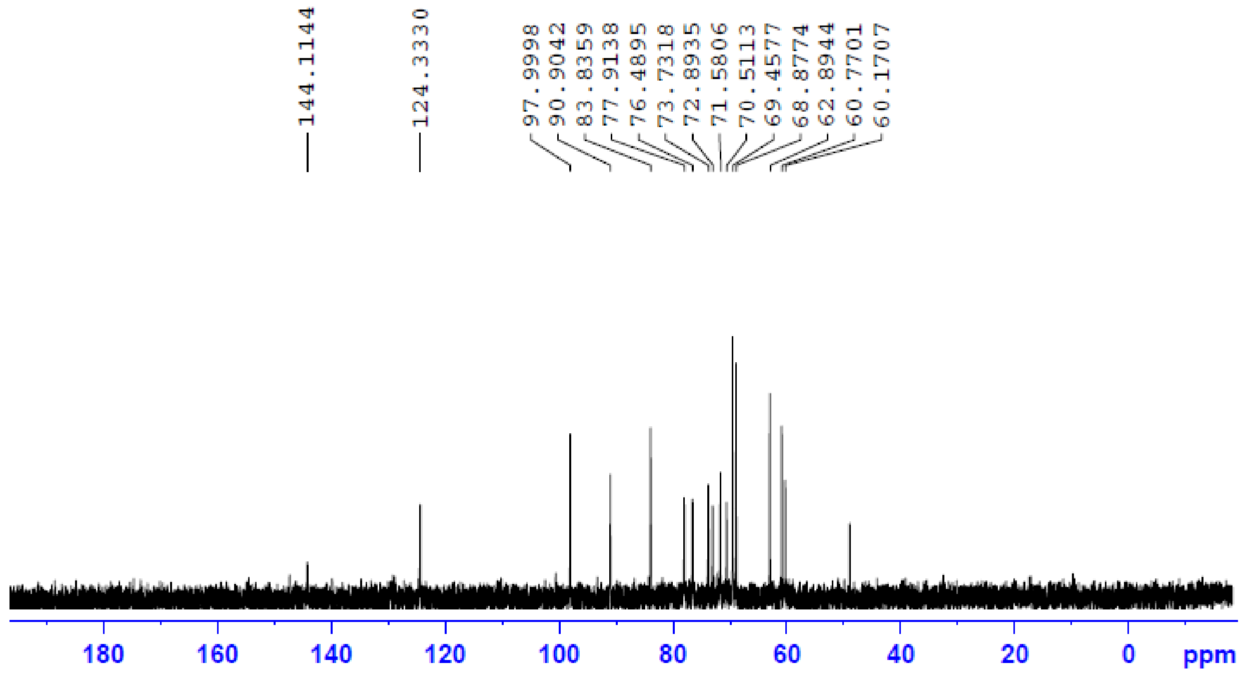

zoom

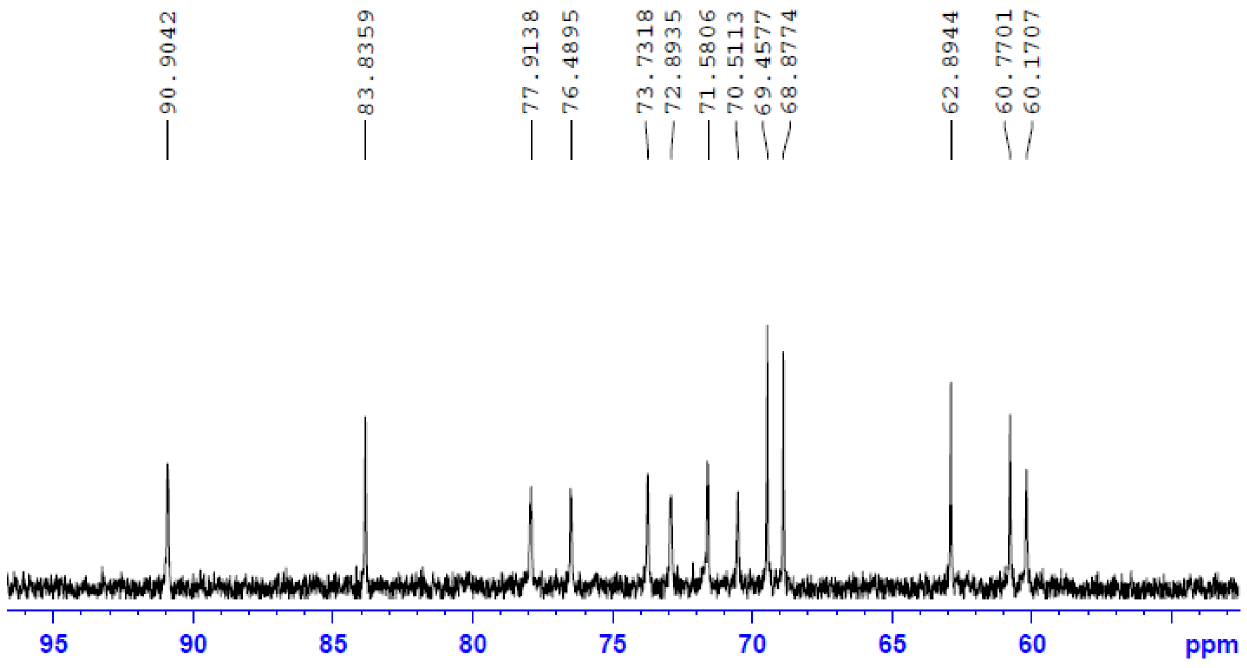

DEPT NMR of 12a in D_2O

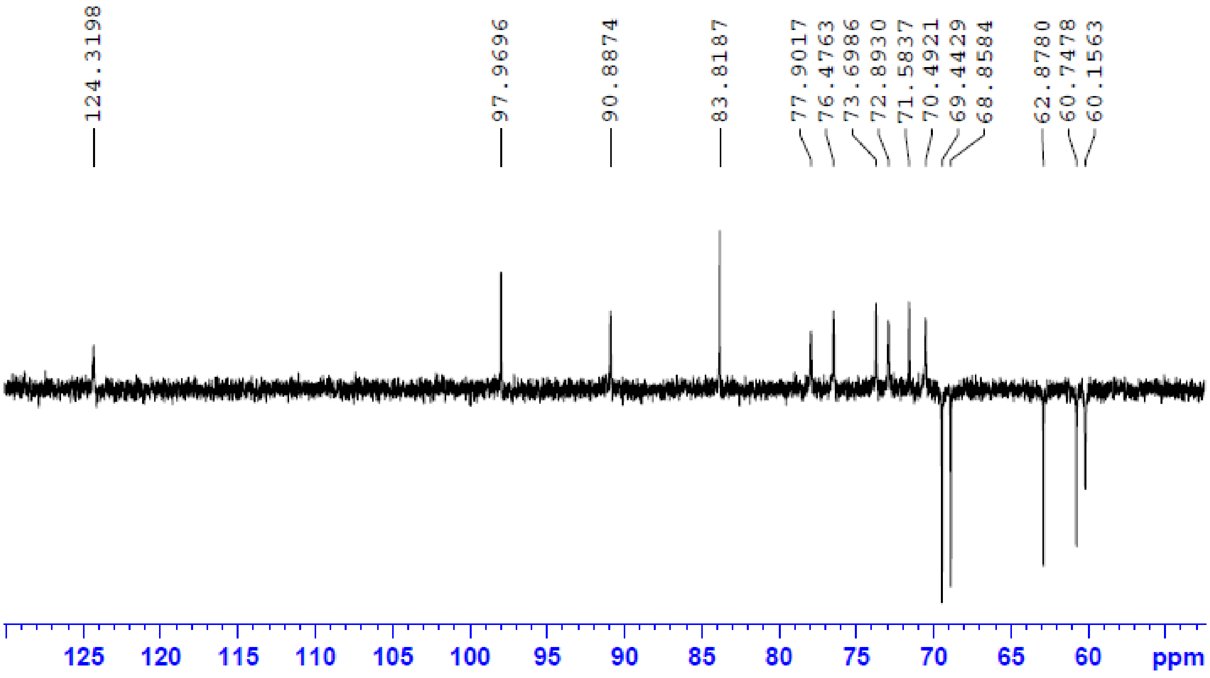

^{31}P NMR of 12a in D_2O

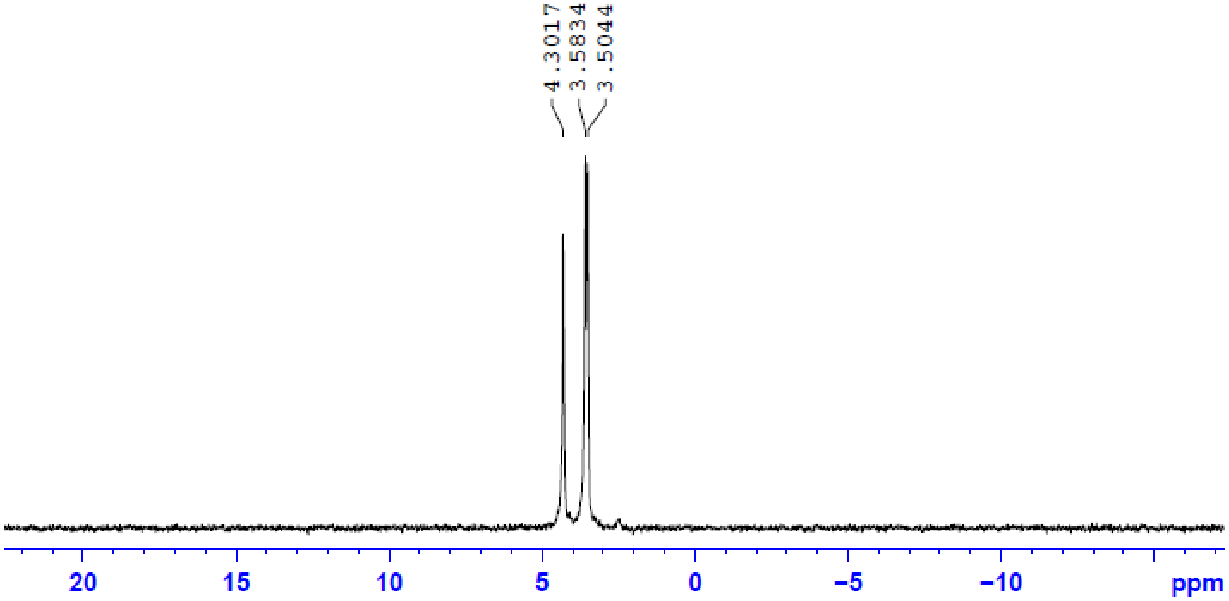

HMQC NMR of 12a in D_2O

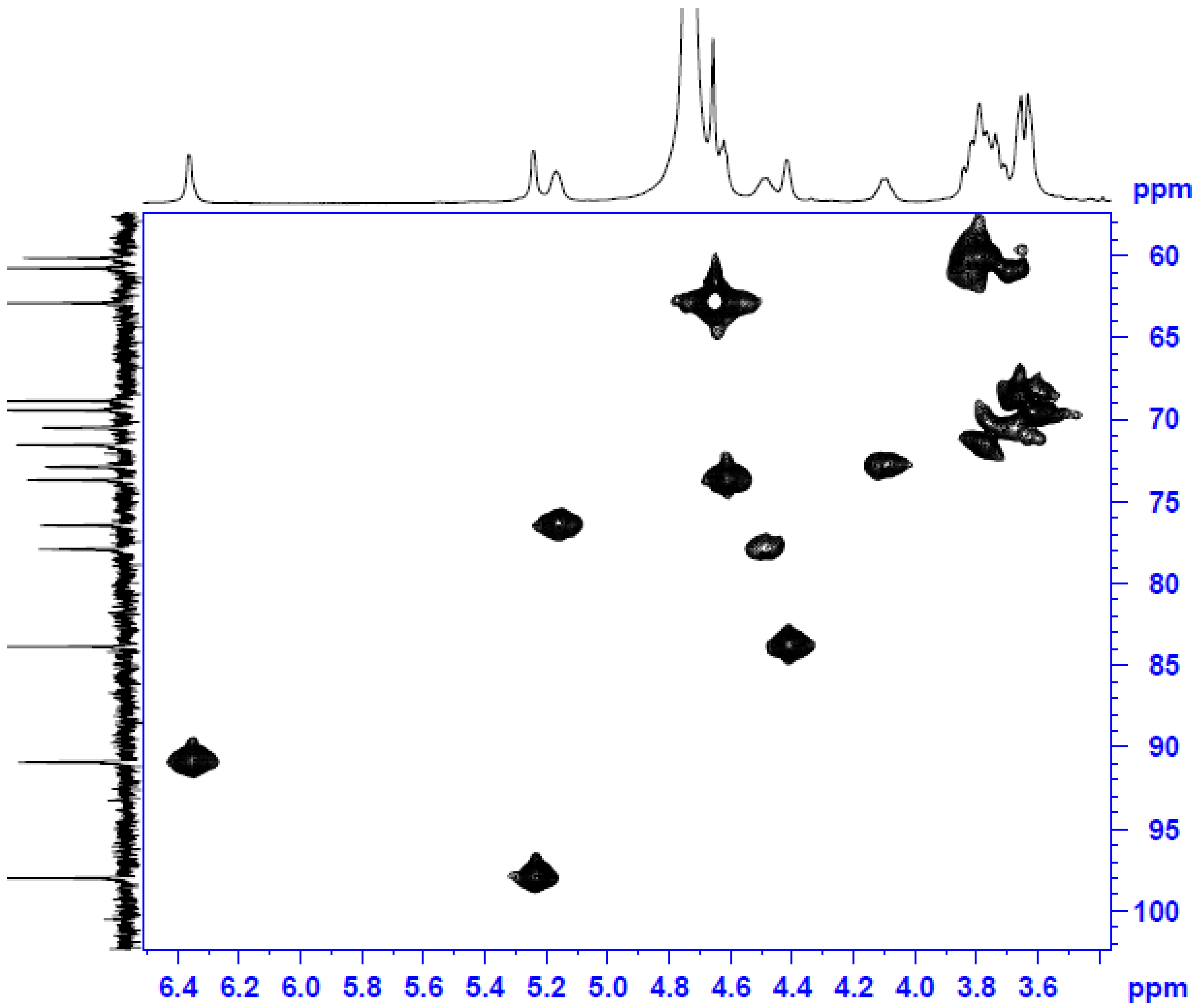

^{1}H NMR of 12b in D_2O

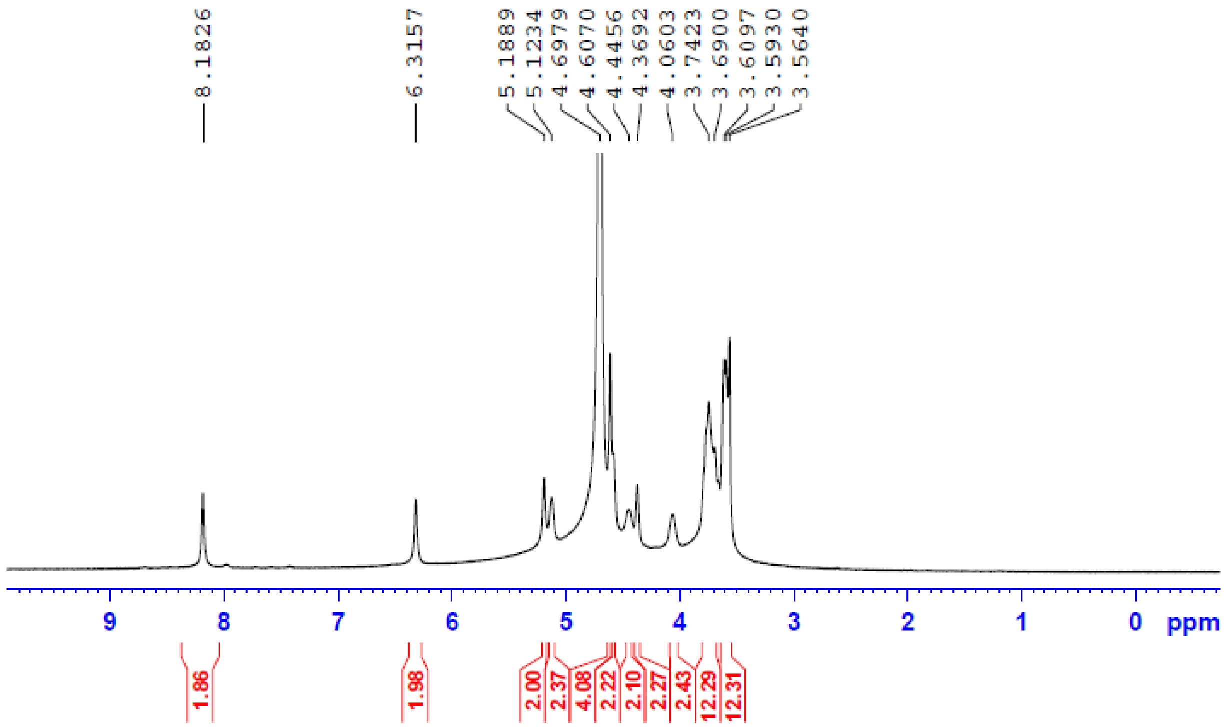

^{31}P NMR of 12b in D_2O

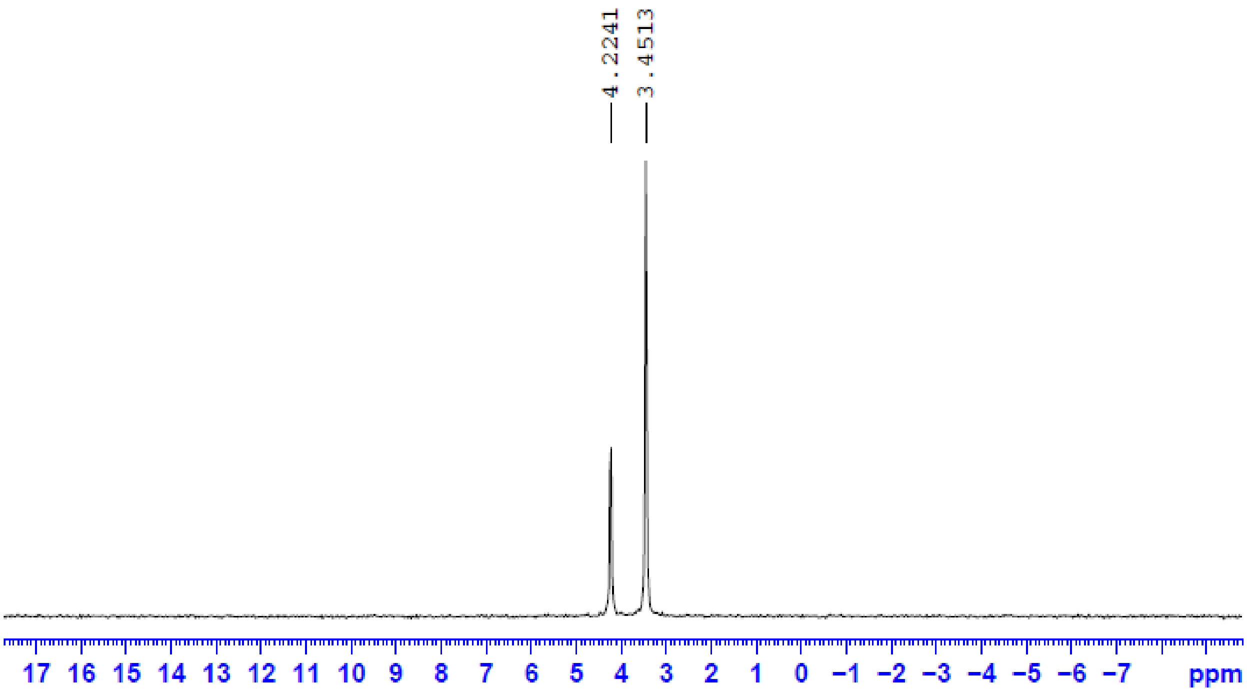

COSY NMR of 12b in D_2O

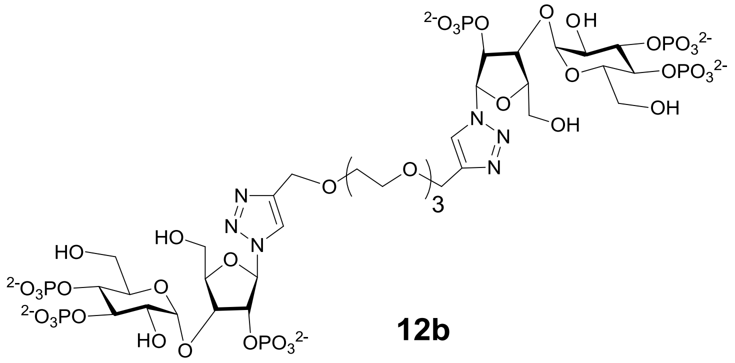

12b

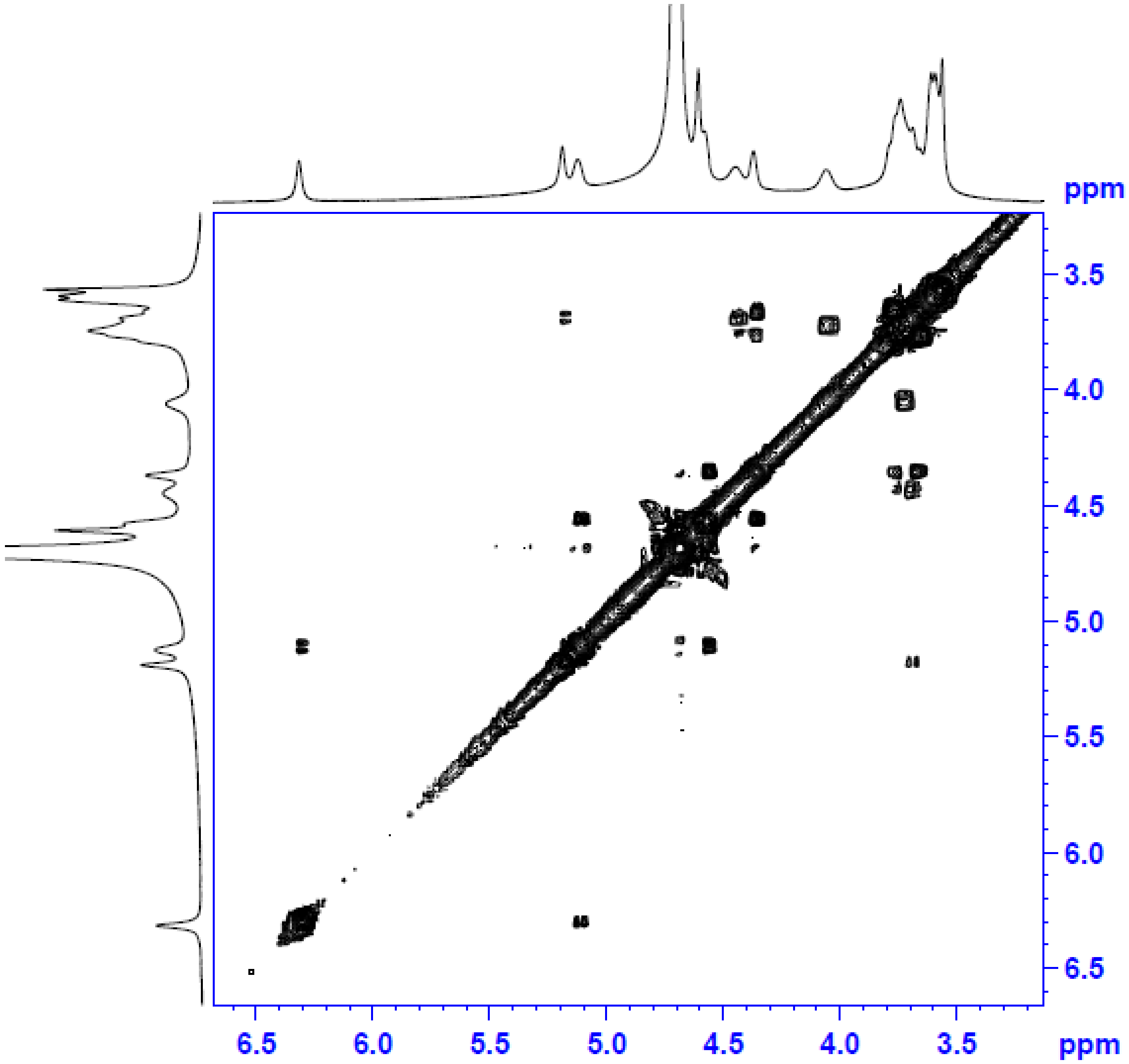

^{13}C NMR of 12b in D_2O

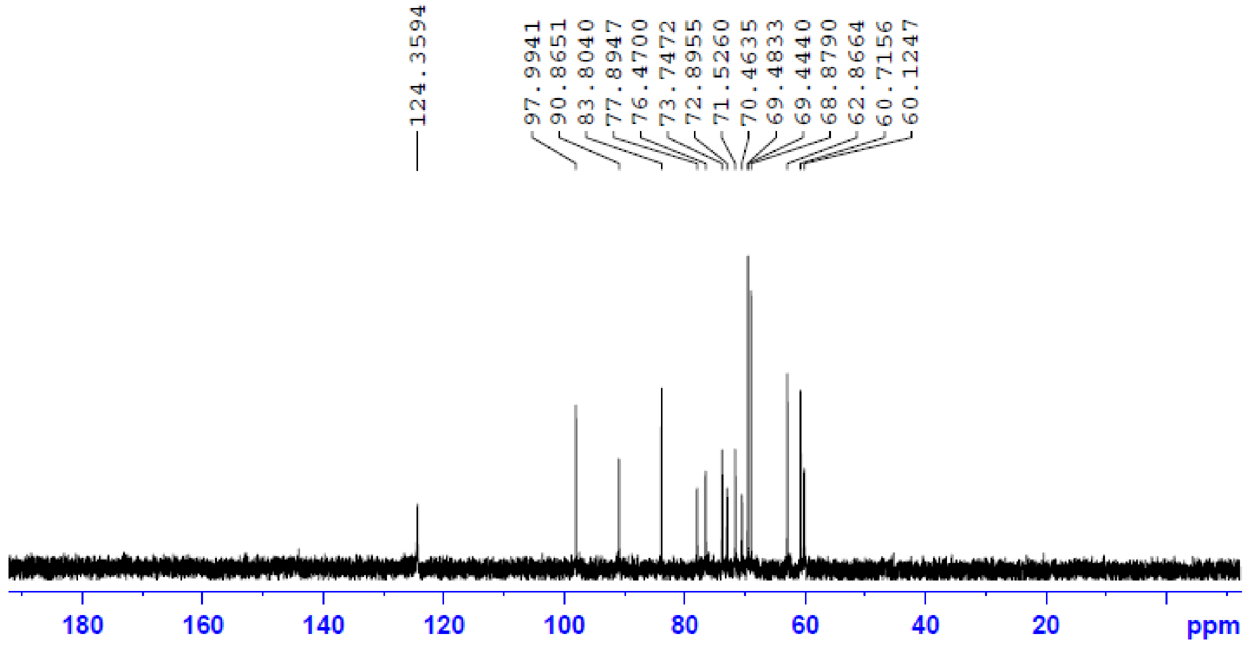

zoom

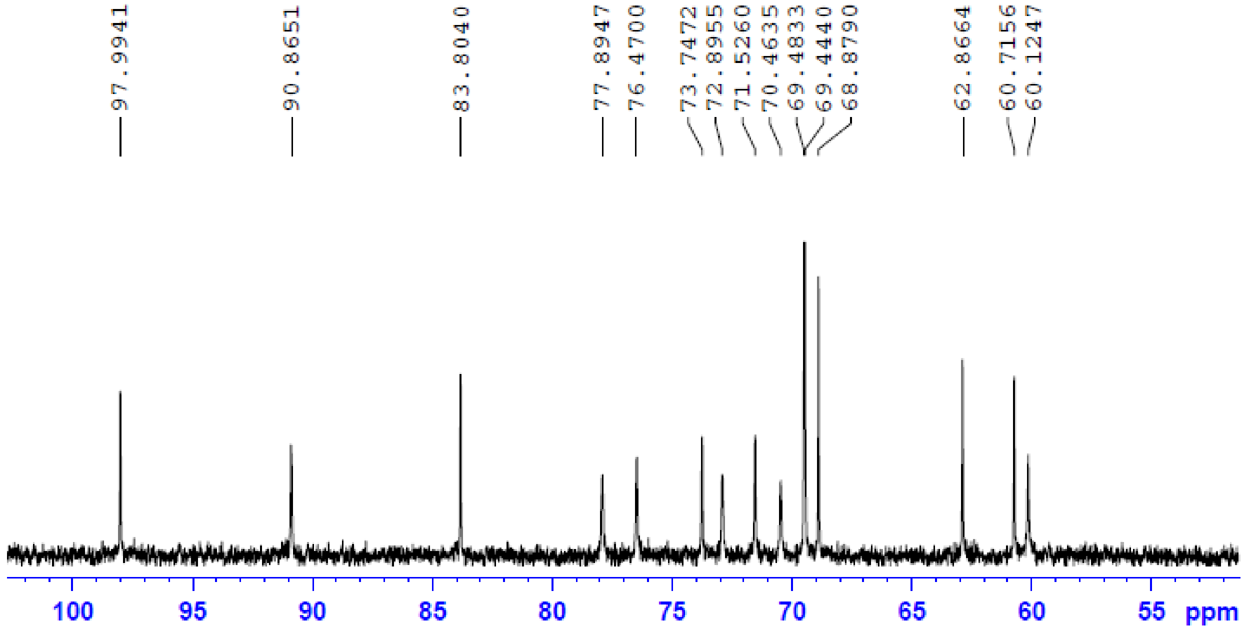

DEPT NMR of 12b in D_2O

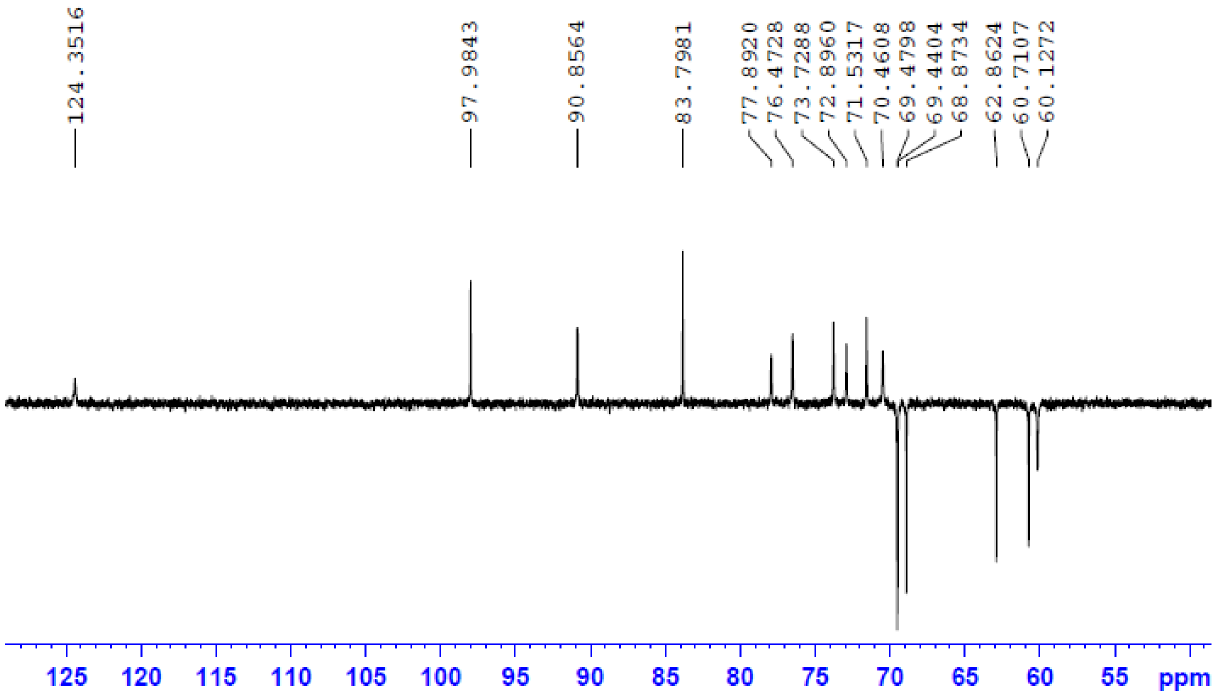

HMQC NMR of 12b in D_2O

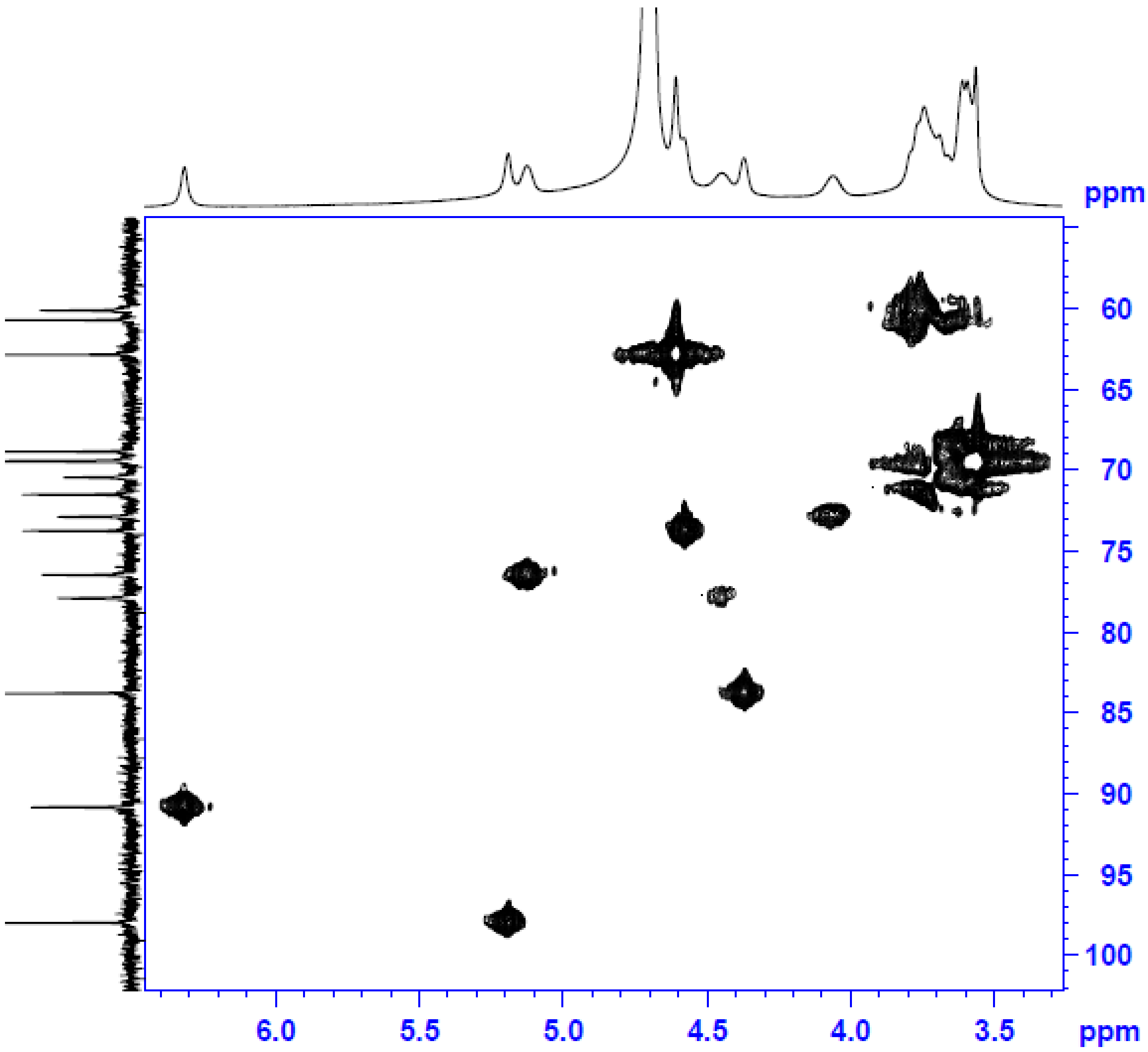

^{1}H NMR of 12c in D_2O

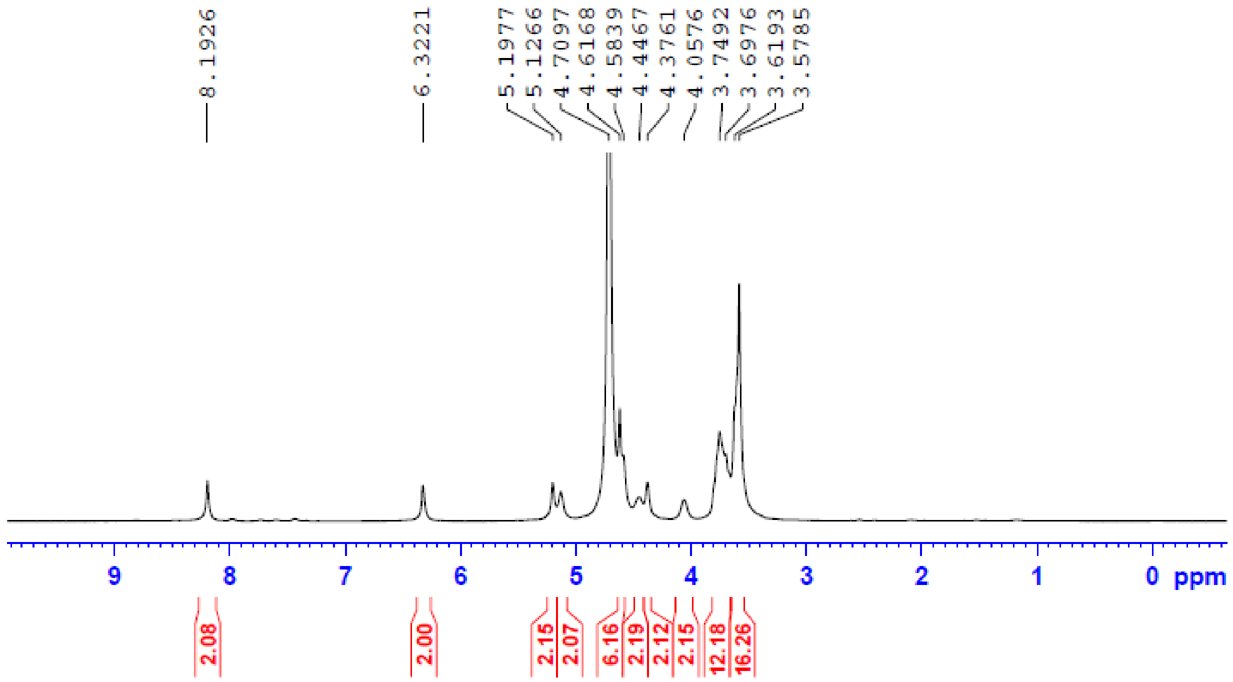

^{31}P NMR of 12c in D_2O

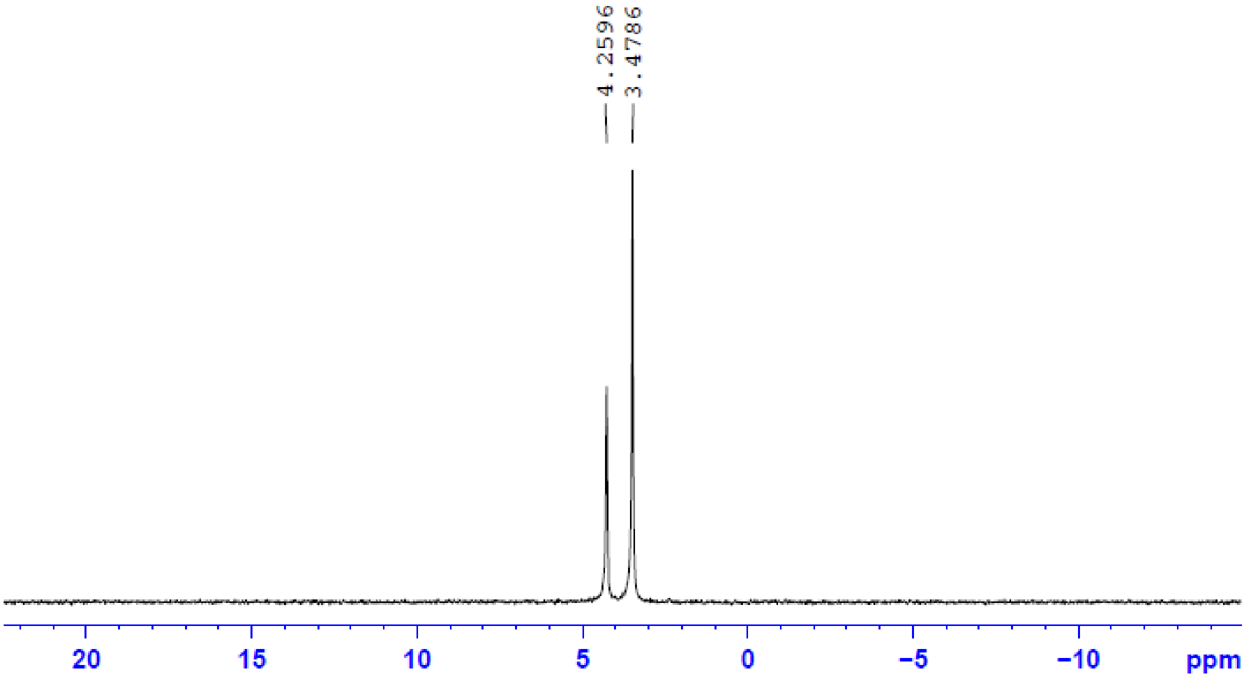

COSY NMR of 12c in D_2O

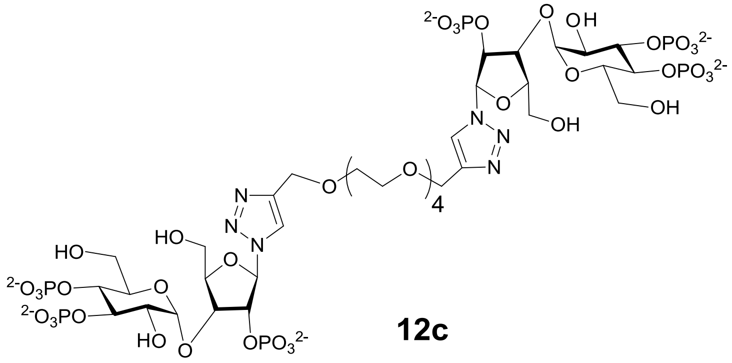

12c

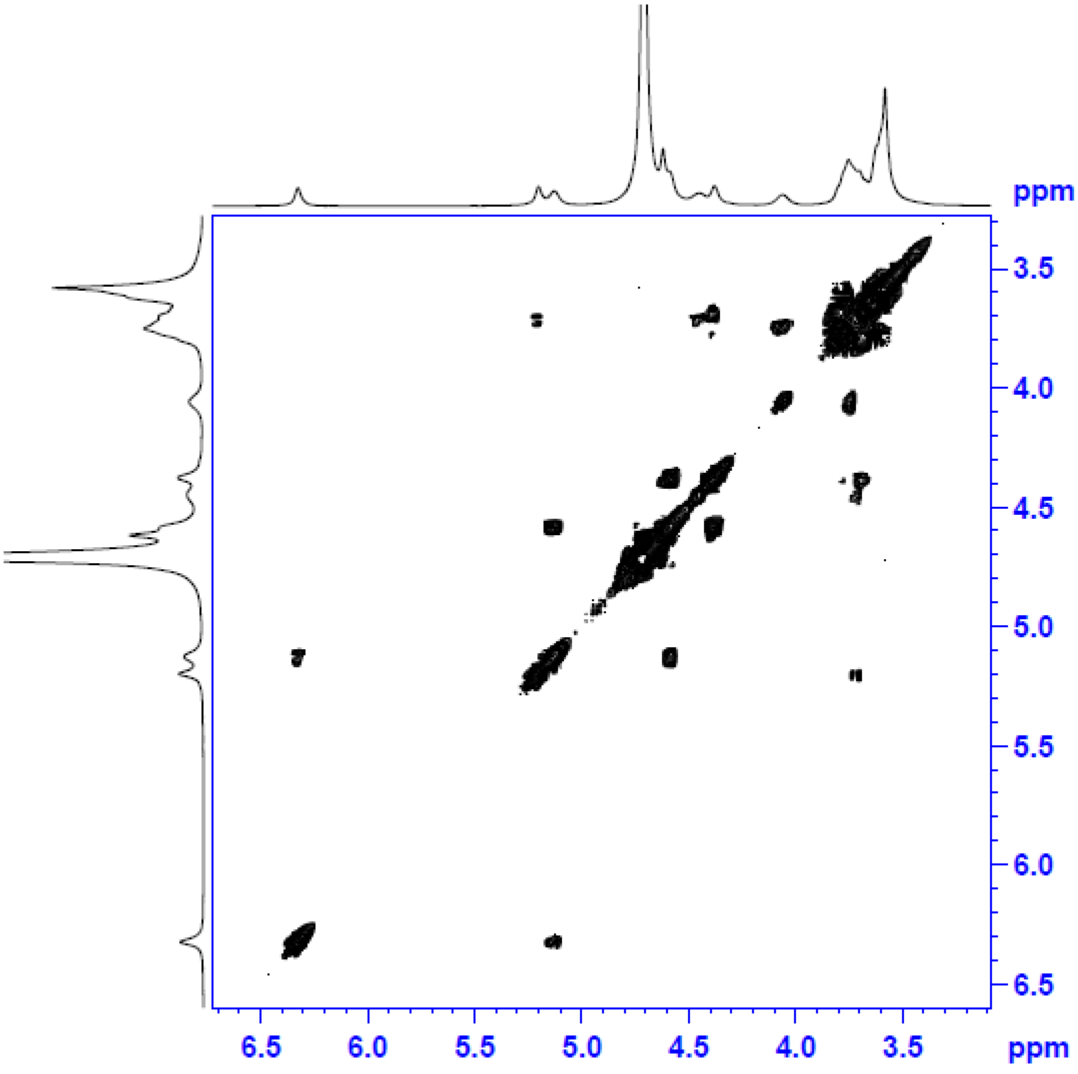

^{13}C NMR of 12c in D_2O

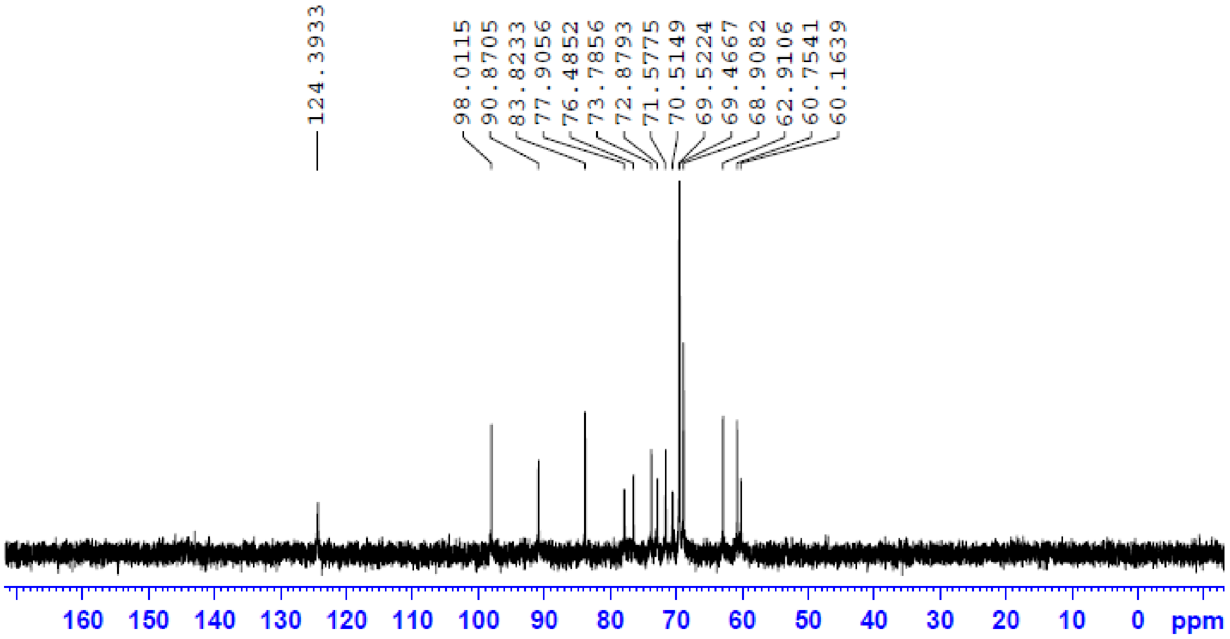

zoom

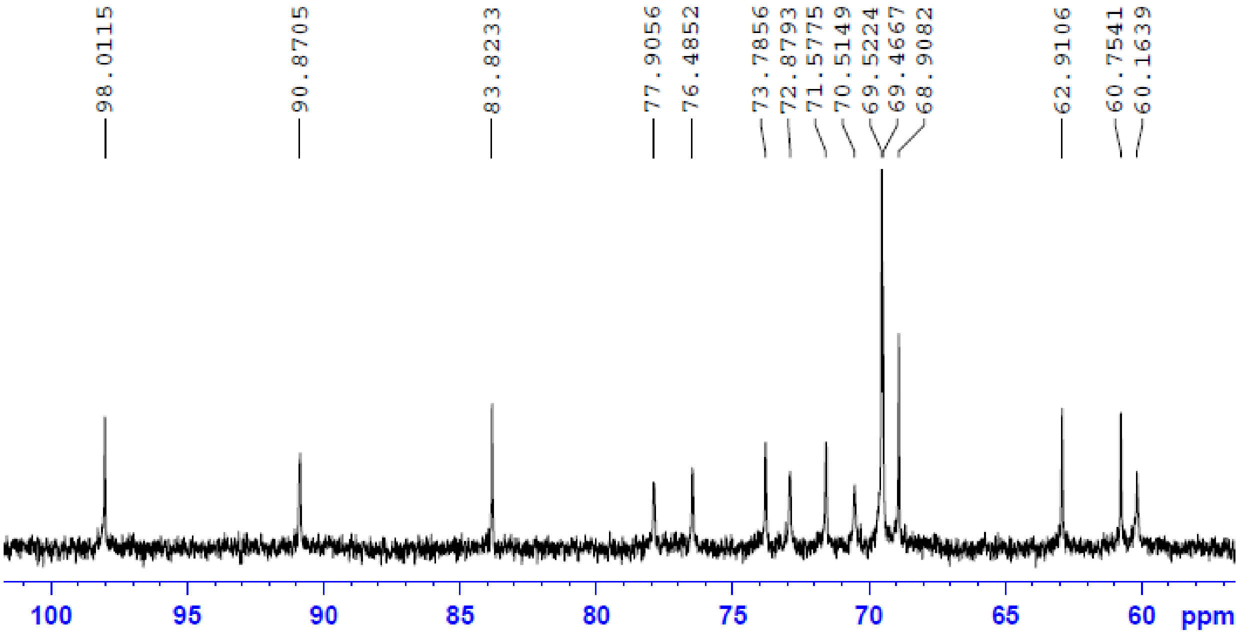

DEPT NMR of 12c in D_2O

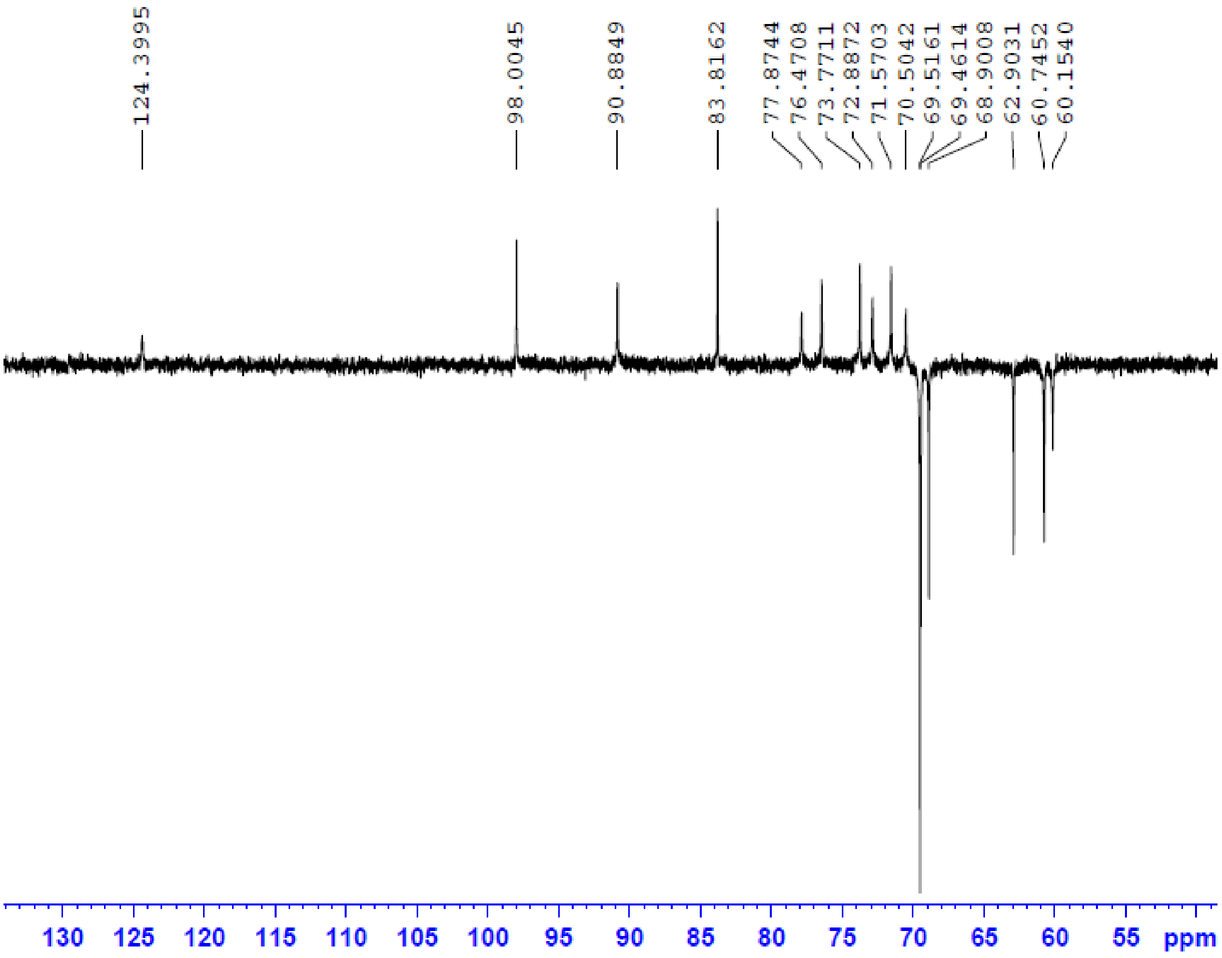

HMQC NMR of 12c in D_2O

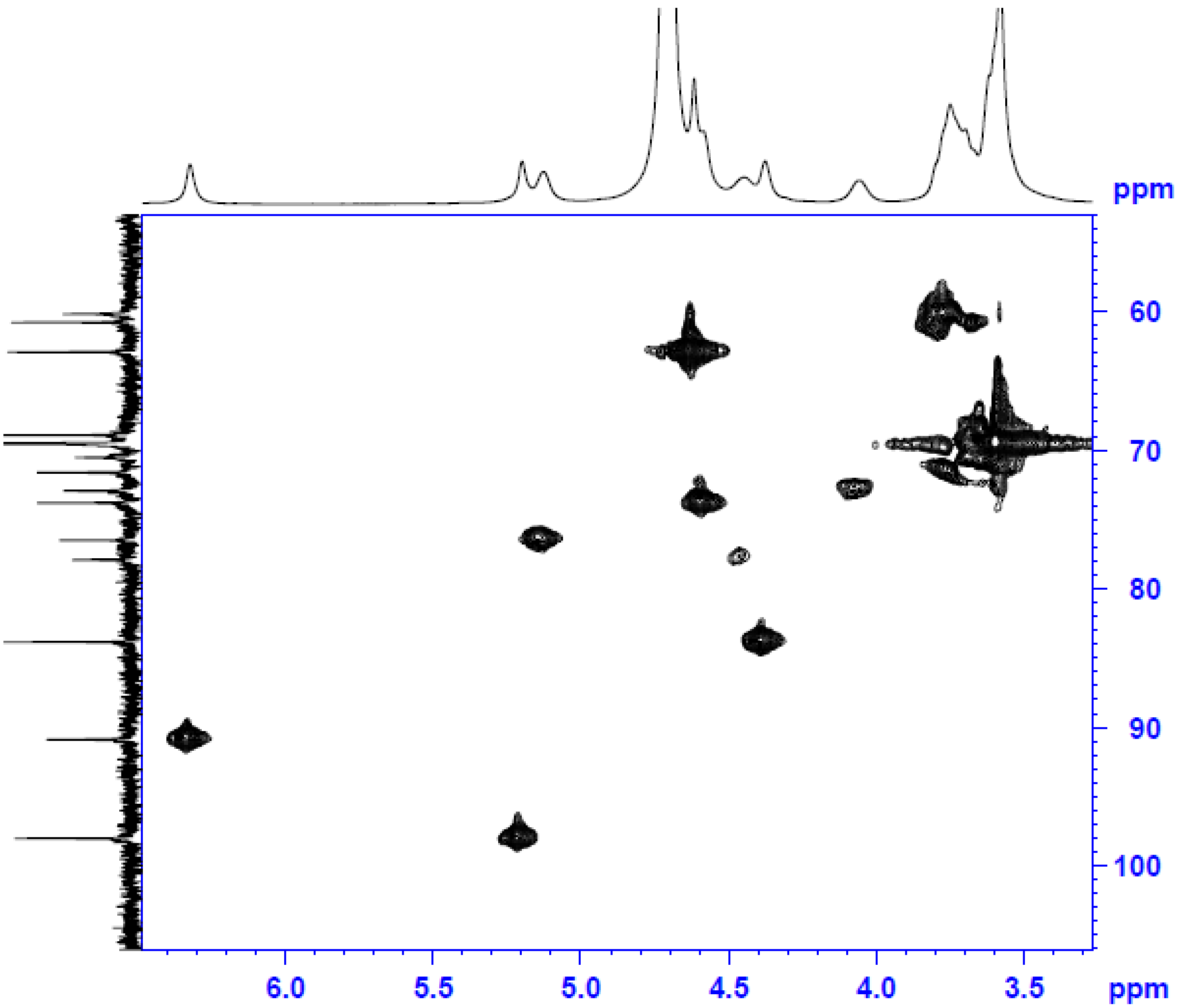

^{1}H NMR of 12d in D_2O

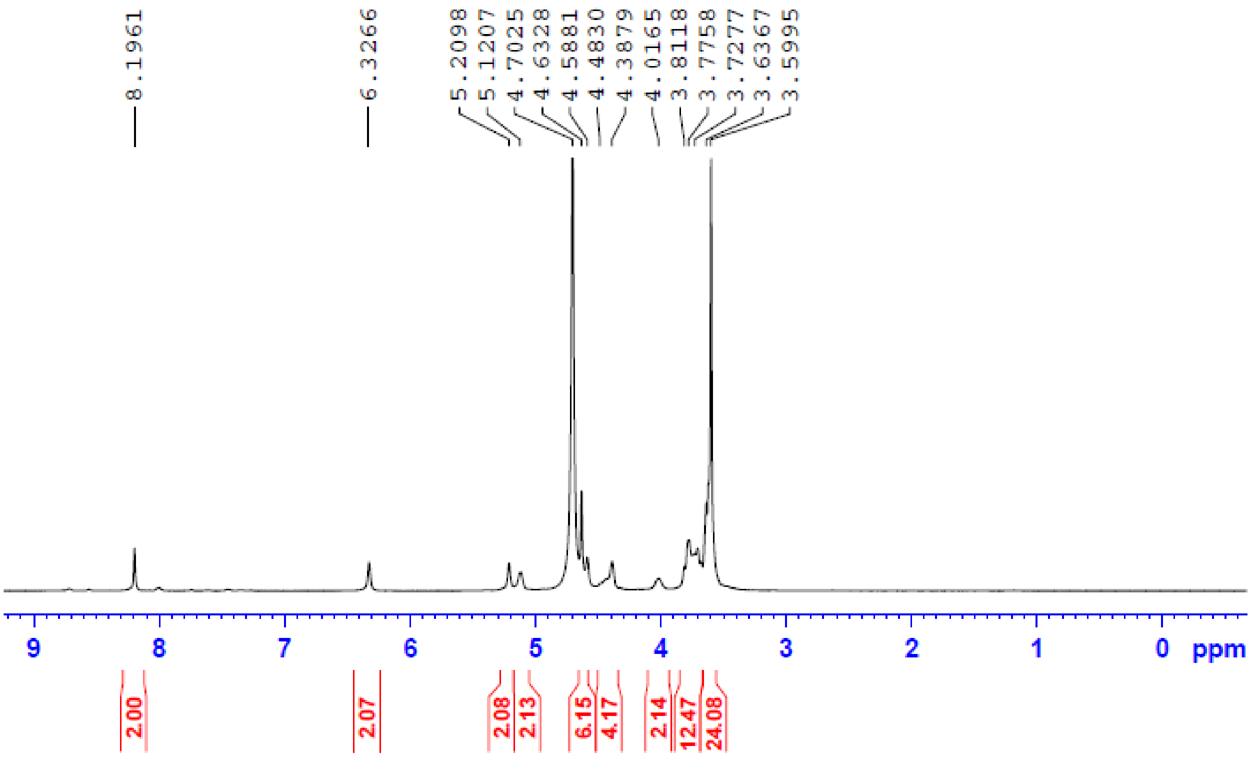

zoom

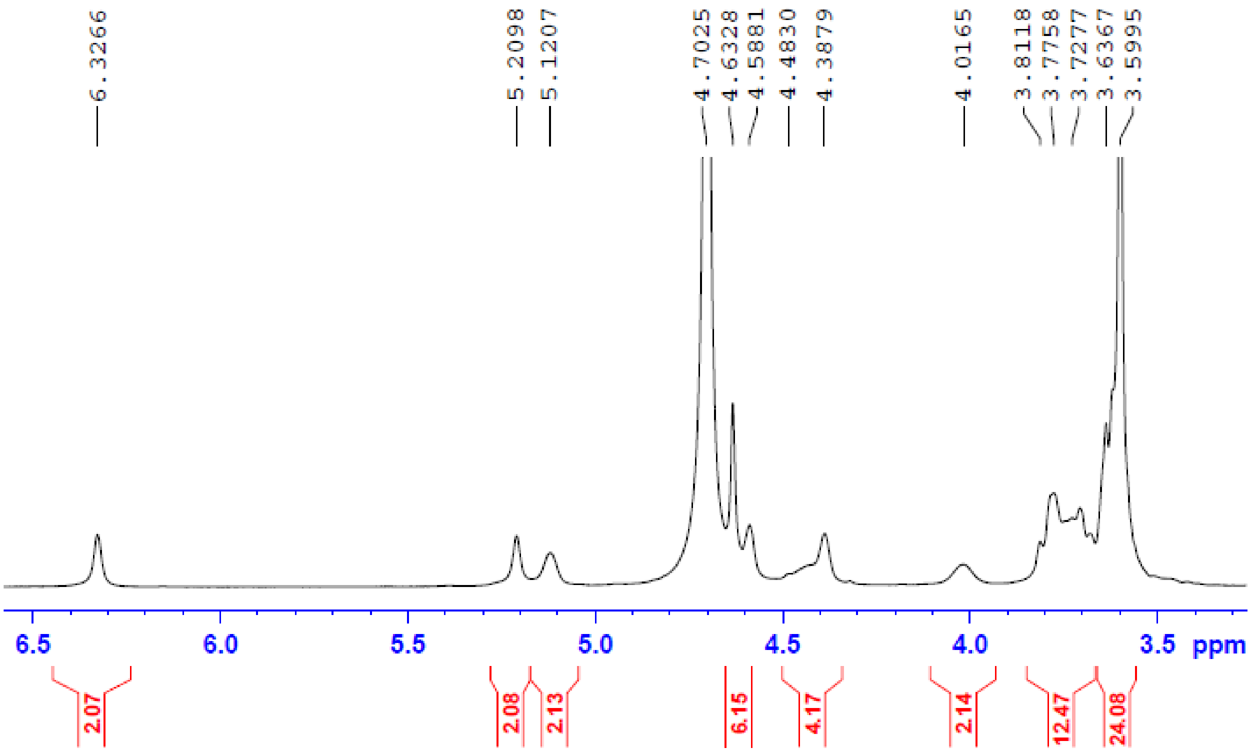

COSY NMR of 12d in D_2O

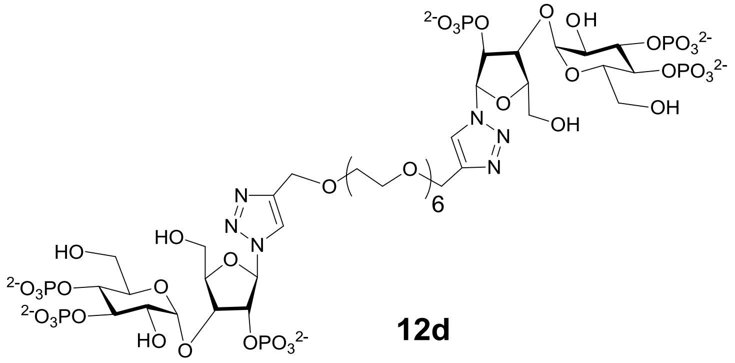

12d

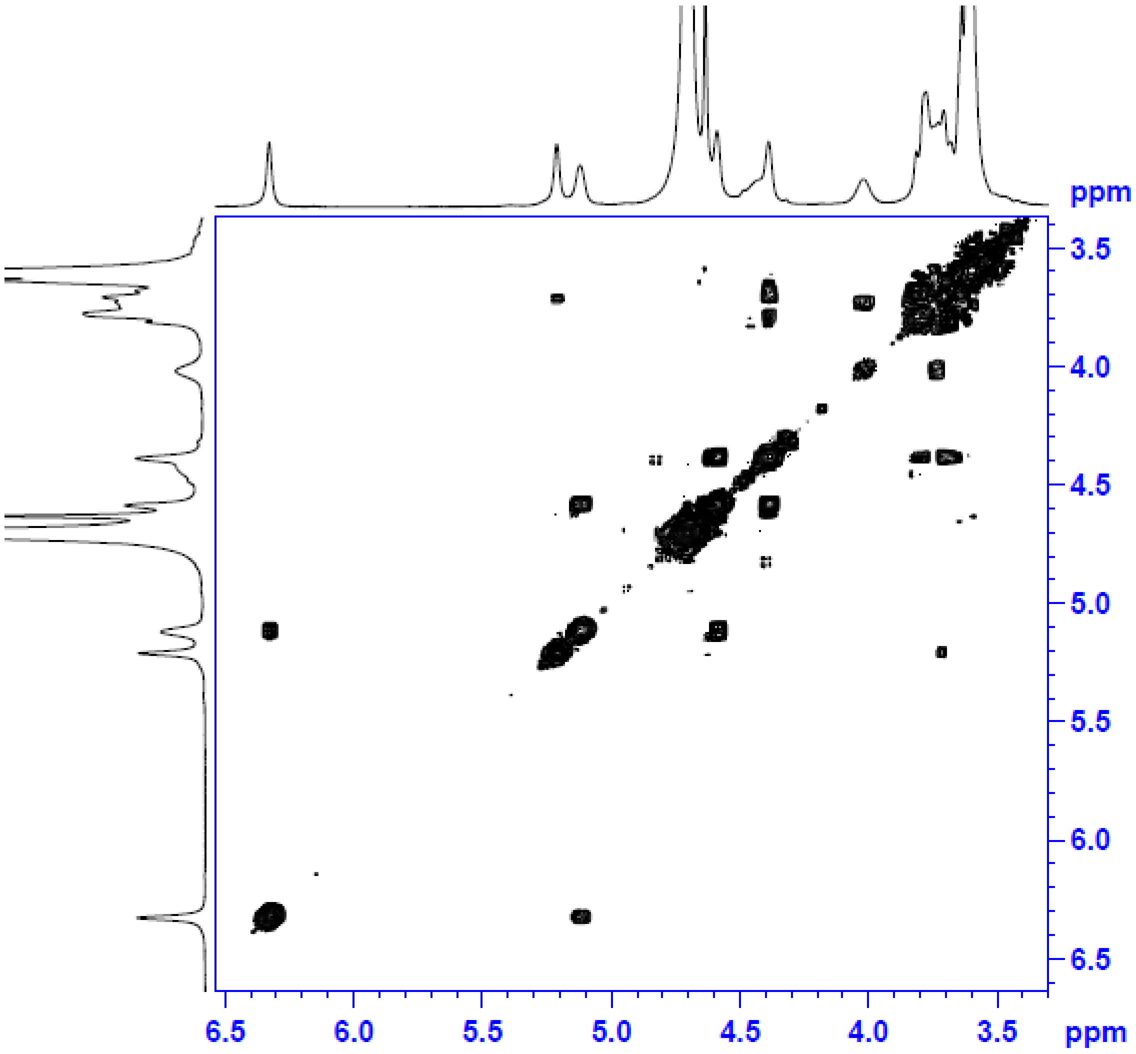

^{13}C NMR of 12d in D_2O

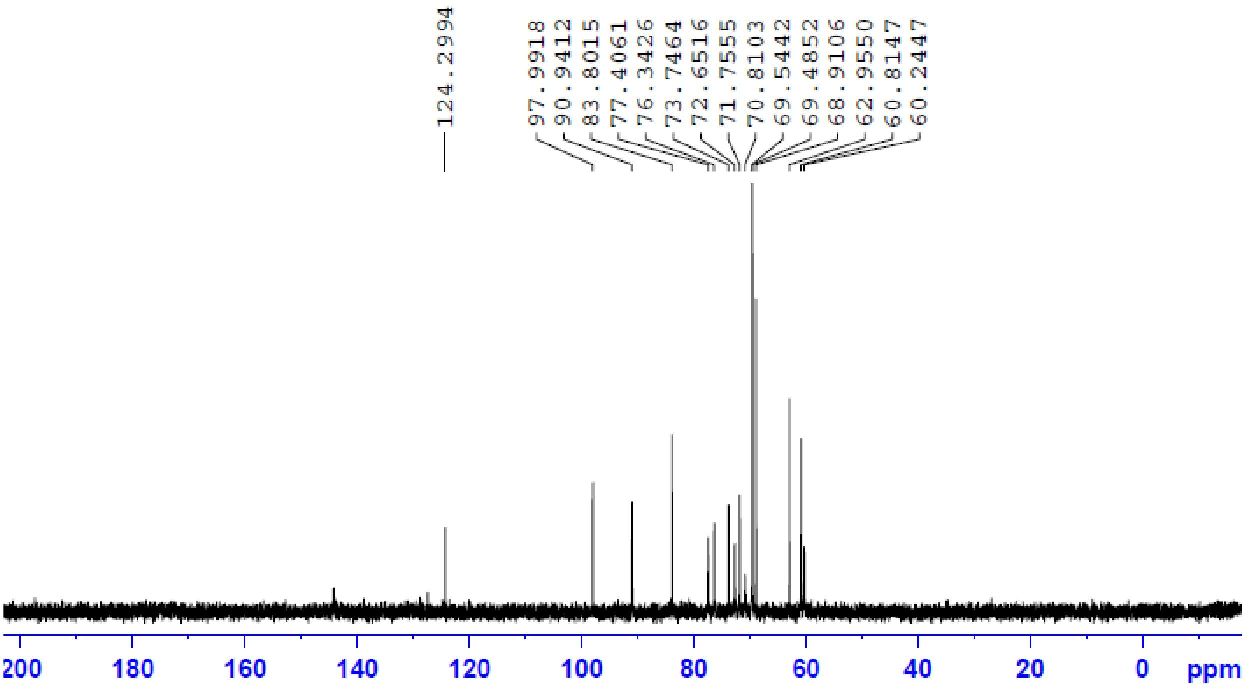

zoom

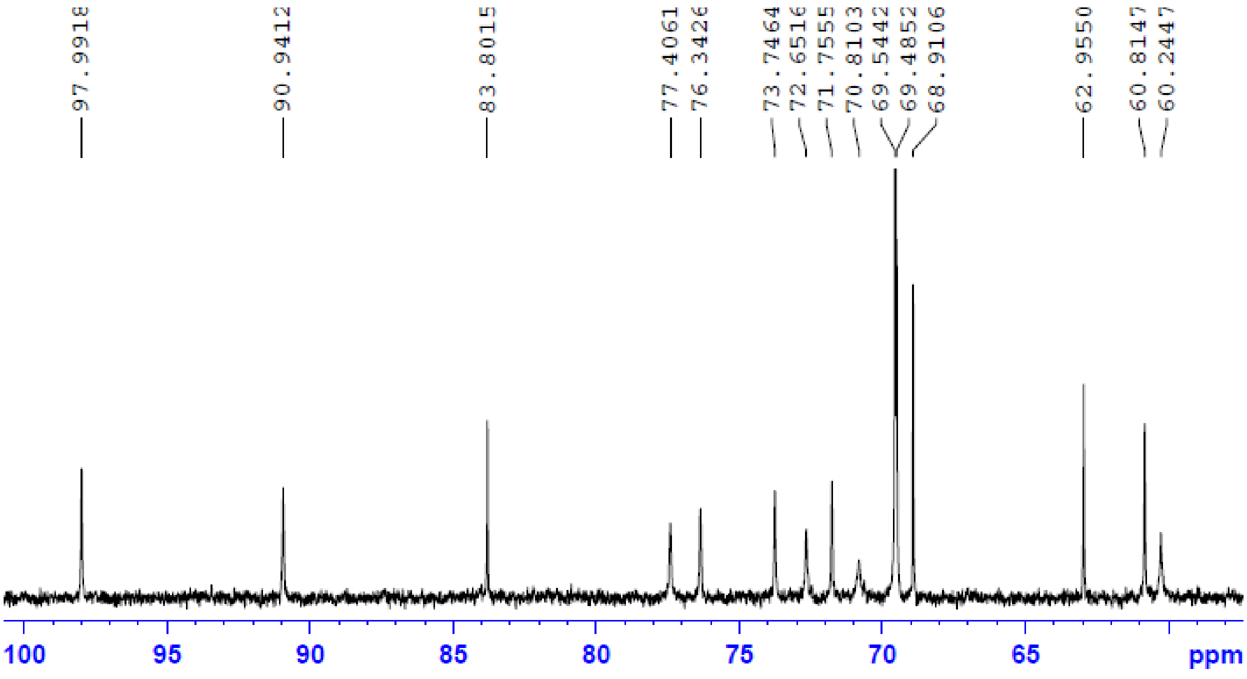

DEPT NMR of 12d in D_2O

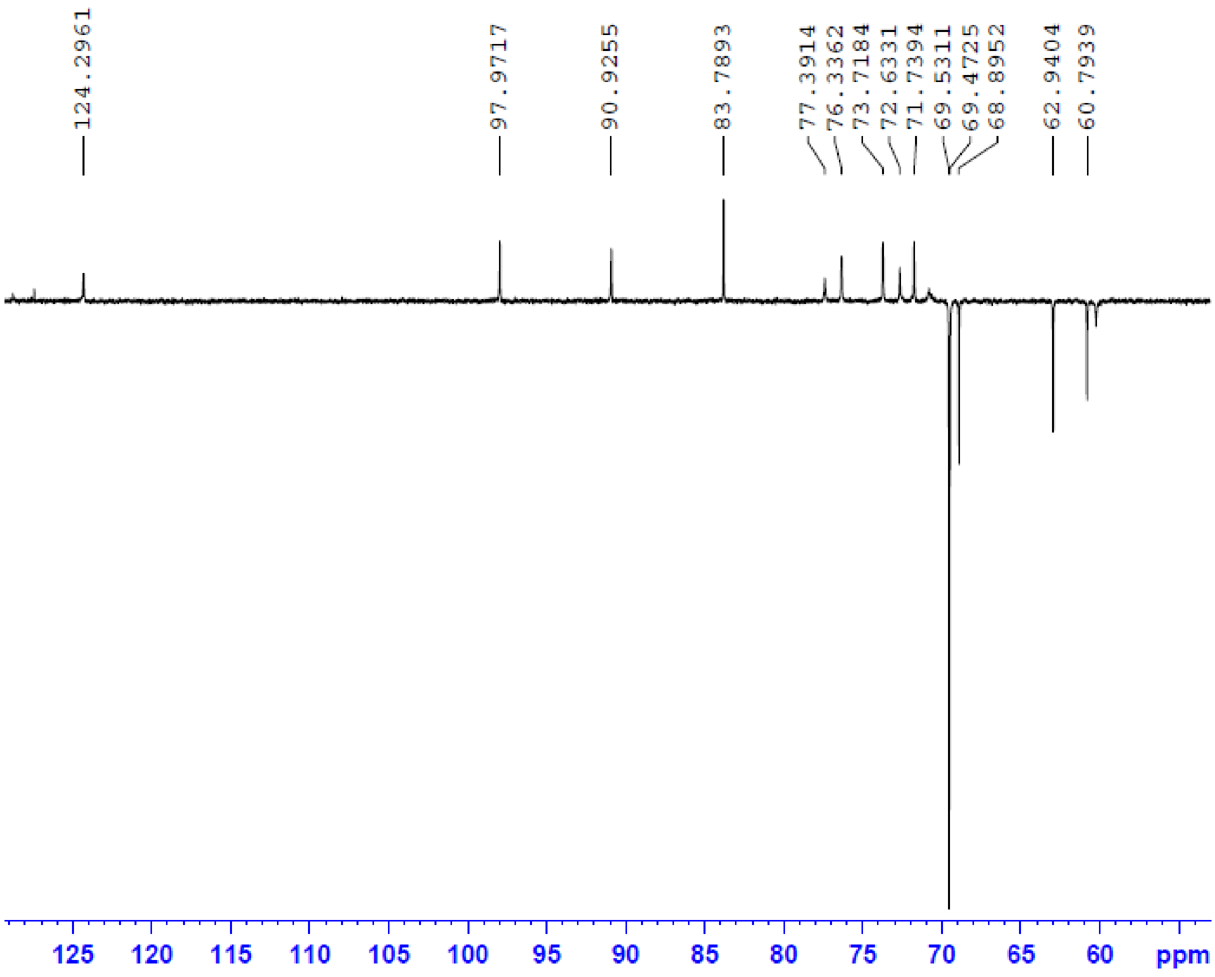

^{31}P NMR of 12d in D_2O

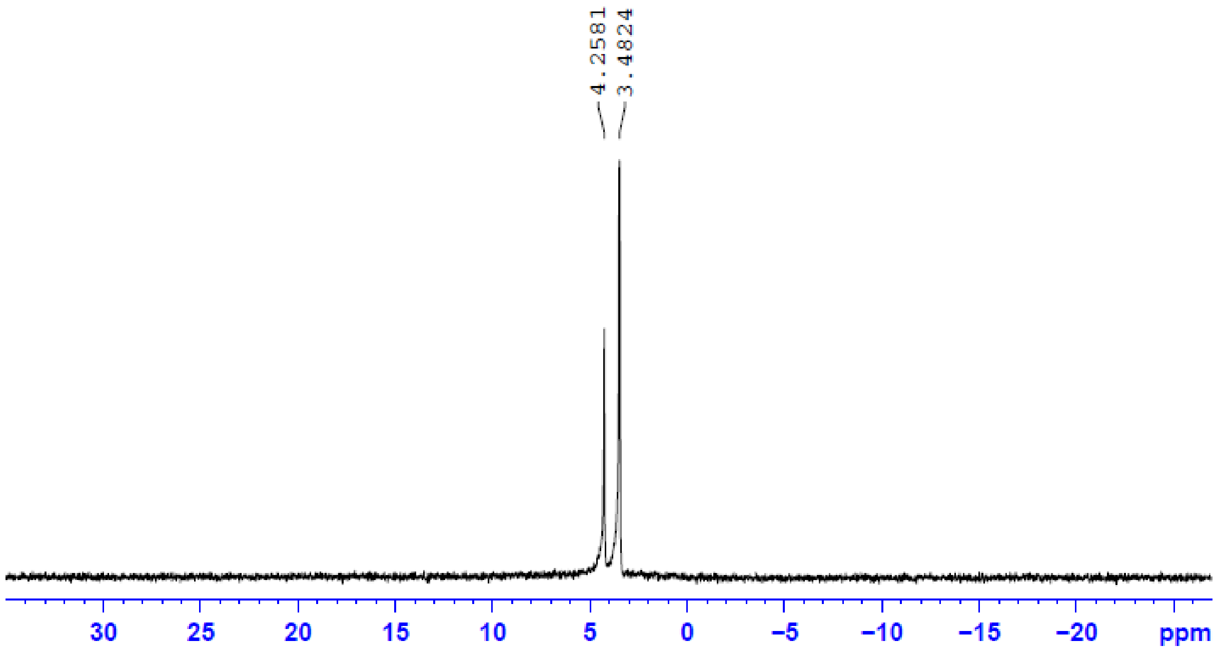

HMQC NMR of 12d in D_2O

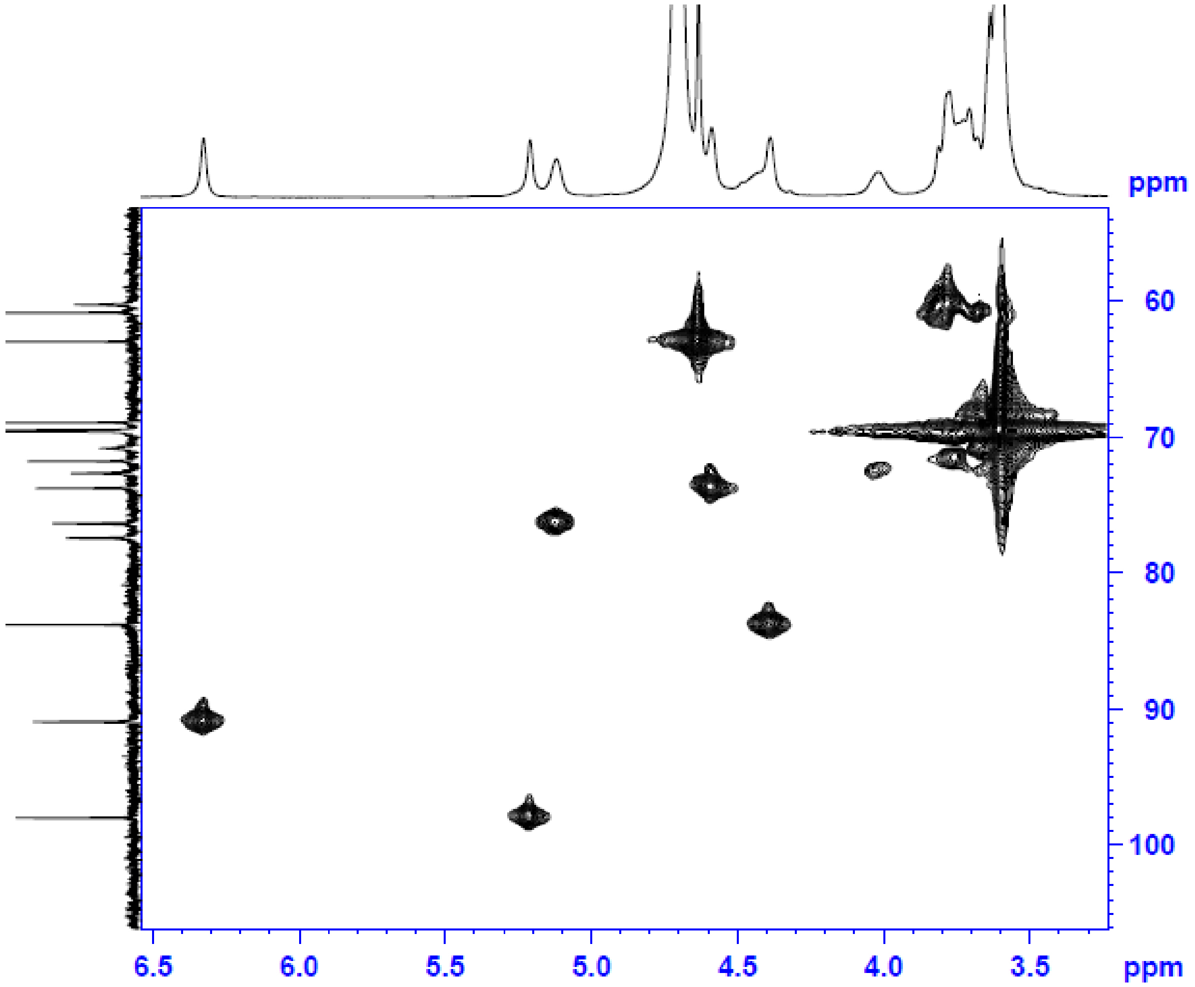

Supplement: Supplementary file 1 [file RA-006-C6RA19413C-s001.pdf]
